# Aquaculture for improving productivity, income, nutrition and women's empowerment in low‐ and middle‐income countries: A systematic review and meta‐analysis

**DOI:** 10.1002/cl2.1195

**Published:** 2021-09-30

**Authors:** Constanza Gonzalez Parrao, Shannon Shisler, Marta Moratti, Cem Yavuz, Arnab Acharya, John Eyers, Birte Snilstveit

**Affiliations:** ^1^ International Initiative for Impact Evaluation London UK; ^2^ Independent Consultant London UK

## Abstract

**Background:**

A steady increase in the international production and consumption of fish has positioned aquaculture as a development option. Previous literature has highlighted the potential of aquaculture to improve economic, nutritional and gender equality outcomes, however, the evidence on the effectiveness of these programmes remains unclear.

**Objectives:**

The review assessed whether aquaculture interventions increase the productivity, income, nutrition, and women's empowerment of individuals. We additionally aimed to identify barriers and facilitators that could affect the effectiveness of these interventions, and the cost‐effectiveness of such programmes.

**Methods:**

We searched for experimental and quasi‐experimental studies focused on low‐ and middle‐income countries. We used standard methodological procedures expected by The Campbell Collaboration for the data collection and analysis.

**Results:**

We identified 21 impact evaluations assessing the effect of 13 aquaculture interventions in low‐ and lower‐middle income countries. Twelve of these studies have a high risk of bias. Aquaculture interventions lead to a small increase in the production value, income, total expenditures and food consumption of participants. The limited availability of evidence prevented us from assessing other nutritional and women's empowerment outcomes. We identified barriers and facilitators affecting the programmes' set up, the participation of beneficiaries, and the level of productive activities. Insufficient cost data hindered full comparisons across programmes.

**Conclusions:**

The review suggests a lack of rigorous evidence assessing the effectiveness of aquaculture programmes. Future research could focus on evaluating nutrition and women's empowerment impacts, promoting reporting standards, and the use of cost data to continue building quality evidence around aquaculture interventions.

## 1 PLAIN LANGUAGE SUMMARY

### 1.1 Aquaculture improves production value, income and nutrition in low‐ and lower‐middle‐income countries

Aquaculture interventions improve the production value, income, total expenditure, and food consumption of beneficiaries. There is insufficient evidence to assess the effectiveness of aquaculture programmes on other nutrition and women's empowerment measures.

### 1.2 What is this review about?

Aquaculture is the farming of aquatic animals and plants in inland and coastal areas. The steady increase in the international production and consumption of fish has encouraged investment in aquaculture as an option for development. While aquaculture is promoted as a pro‐poor activity with the potential to stimulate the economy, increase the consumption of nutritious food, and drive gender equality, there is still limited rigorous evidence regarding its impact.

We defined “aquaculture interventions” as any project, programme or policy aiming to provide new and/or improved activities at any stage of the aquaculture value chain. No further restrictions were defined a priori for identifying relevant interventions.

The review assessed whether aquaculture interventions increase the productivity, income, nutrition and women's empowerment of individuals in low‐ and middle‐income countries. We also aimed to identify barriers and facilitators that could affect the effectiveness of these interventions, and the cost‐effectiveness of such programmes.

**What is the aim of this systematic review?**

The aim of this review is to assess whether aquaculture interventions increase productivity, income, nutrition and women's empowerment. It also identifies barriers and facilitators that could affect the effectiveness of these interventions, and the cost‐effectiveness of such programmes.

### 1.3 What studies are included?

The review includes studies with an experimental or quasi‐experimental design that estimate the effect of aquaculture interventions on relevant outcomes.

We identified 21 studies covering 13 aquaculture programmes in low‐income and lower‐middle‐income countries, with the majority focusing on Bangladesh. We did not identify relevant studies implemented in middle‐income countries.

### 1.4 What are the main findings of this review?

#### 1.4.1 Do aquaculture interventions increase the productivity, income, nutrition and empowerment of individuals?

There is a small increase in the production value, income, total expenditure and food consumption of participants as a result of their involvement in aquaculture interventions.

These findings should be interpreted with caution given the substantial heterogeneity and potential for risk of bias in the included studies. There is not enough evidence available to synthesise other nutrition outcomes—such as anthropometrics, food security, or quality of diets—or women's empowerment measures. Moreover, there is insufficient data to assess spillover effects, or if the effect of aquaculture interventions differs by gender.

#### 1.4.2 What are the potential barriers and facilitating factors that affect the effectiveness of aquaculture interventions?

First, barriers affecting programme set up are low funding, participants not being able to choose the intervention package, unclear roles of partners, and project plans that were never implemented. Second, we find barriers and facilitators affecting the participation of beneficiaries, including social and cultural norms, the level of income generated from aquaculture activities, programme delivery aspects, and access to natural capital. Third, we identify factors affecting the level of productive activities, involving access to inputs and funding, general economy settings and infrastructure and environmental issues.

#### 1.4.3 What is the cost‐effectiveness of aquaculture interventions?

There is insufficient data to make full comparisons across programmes. For interventions in Bangladesh, the maximum yearly cost per household is US$300, while the maximum benefits are US$900. The lowest cost for reaching a household is US$19 per annum.

### 1.5 What do the findings of this review mean?

The results of the review suggest that, while several aquaculture programmes can be identified, there is a paucity of rigorous evidence assessing their effectiveness. This opens an opportunity for the aquaculture programming sector to align investments with evaluation frameworks that inform what works, for whom, and why.

Future research could emphasise three areas to continue building quality evidence:

1. Establish ways to evaluate the effect of aquaculture interventions on intermediate and main nutrition outcomes and women's empowerment measures;
2. Promote reporting standards to reflect that relevant studies are free from confounding issues; and
3. Encourage the collection and publication of cost data to allow for cost‐effectiveness analyses across the sector.

### 1.6 How up‐to‐date is this review?

The review authors searched for relevant studies in November 2020.

## 2 BACKGROUND

### 2.1 The problem, condition or issue

In 2018, global fish production reached a record high of about 179 million tonnes, of which 82 million tonnes, valued at USD 250 billion, came from aquaculture production, which is the farming of aquatic organisms including fish, molluscs, crustaceans, and aquatic plants in inland and coastal areas (FAO, 2020a). While global fish production has seen important increases across all continents in the last 20 years, it has almost doubled in Africa and Asia. Over 20 million people are estimated to be engaged on a full‐time, part‐time or occasional basis in aquaculture, making this sector an important source of employment and income across the world. Women account for 19% of this workforce and play a crucial role throughout the aquaculture value chain, providing labour in both commercial and artisanal fisheries (FAO, 2020b).

The growth in aquaculture production has also brought substantial changes in the production systems, raising concerns about the environmental impact of aquaculture and the sustainability of the sector. These detrimental effects include, among others, poor site selection; the use of chemicals and antimicrobials; the impact of escapees on wild stocks; inefficient or unsustainable production of fishmeal and fish oil; or eutrophication (FAO, 2020b; Henriksson et al., 2017). Similarly, the increase and intensification of aquaculture activities can pose a major pressure on land and its use whenever they require converting the use of land into ponds for farming purposes. For example, the shrimp aquaculture sector, successfully established in the 1970–1980s, has been the major cause of mangrove deforestation in Southeast Asia over the last few decades (Richards & Friess, 2016; Valiela et al., 2001). This has been especially controversial since mangroves are an important carbon sink, they support fisheries, provide coastal protection, and their loss and degradation reduce coastal resilience (Barbier et al., 2011; Koh et al., 2018; Mcleod et al., 2011).

To offset these adverse effects and improve governance of the aquaculture sector, the Food and Agriculture Organization of the United Nations (FAO) has championed the Blue Growth Initiative as a framework for a sustainable, economic and social development of fisheries and aquaculture (FAO, 2014a). Examples of practices following this framework include conservation‐oriented management interventions to achieve sustainable coastal aquaculture, implementing protected areas and land zoning to regulate the development of commercial aquaculture, and introducing sectoral innovations, from government support to farmer training and better feeds, to help reduce the environmental footprint of aquaculture (Akber et al., 2020; Henriksson et al., 2017).

Despite the environmental challenges that have arisen from increased production in the sector, aquaculture seems to have great potential to address poverty and nutrition issues, considering that 80% of the world production comes from developing countries (Phillips et al., 2016) and that over 80% of the global aquaculture production is from small‐scale farms that are commonly owned and managed by families (FAO, 2014b). Therefore, in a world of limited resources, aquaculture may have the ability to improve livelihoods and health in developing countries and to contribute to the progress towards a number of inter‐related Sustainable Development Goals (SDGs).

For example, aquaculture could help reduce hunger (SDG 2) and poverty (SDG 1) by making fish available and affordable to combat malnutrition and alleviate nutritional deficiencies (SDG 3: Good health and well‐being). By engaging women into its workforce, aquaculture also has the potential to promote greater equity in access to, and benefits from, economic resources (SDG 5: Gender equality). Finally, aquaculture can contribute to more sustainable development (SDG 14: Conserve and sustainably use the oceans, seas and marine resources for sustainable development) by supporting the production of low carbon footprints among animal source foods (Reale & Phillips, 2020). Thus, well‐planned aquaculture operations could be a key component in sustainable food systems, capable of providing needed animal‐source foods to an increasingly growing population.

Aquaculture is often promoted as a pro‐poor economic activity by acting as a source of income to secure livelihoods for rural populations in low‐ and middle‐income countries (Dey & Ahmed, 2005; Mohamed & Dodson, 1998; Olaganathan & Kar Mun, 2017). However, the scarce empirical evidence around this topic shows a more nuanced picture, in which the impact depends on local production and consumption characteristics of the sector. Recent studies in Ghana (Kassam & Dorward, 2017) and Bangladesh (Rashid et al., 2019) have suggested that aquaculture can have a positive impact on economic growth and poverty reduction at a national level. However, evidence has also highlighted that promoting aquaculture could benefit primarily larger and better‐off farms, thus increasing inequality (Ahmed et al., 1995; Kassam & Dorward, 2017).

The global increase in fish production seems to correspond with a general expansion in fish consumption. The consumption of fish products has increased at an average annual rate of around 3% from the 1960s, a rate higher than all other animal protein foods, and this growth has been observed in both developed and developing countries (FAO, 2020b). Thus, aquaculture has the potential to increase the supply and accessibility of nutritious food that could translate into more nutritious and diverse food diets. Relevant studies have found that agriculture interventions often lead to an increase in food consumption, particularly for the food item targeted by the intervention. Yet the impact of aquaculture on diet quality is more unclear, with evidence being scarce and mixed, often due to the lack of high‐quality studies and data (Bird et al., 2019; Kawarazuka, 2010; Masset et al., 2012).

Likewise, very little is known about the impact of aquaculture activities on the income, livelihood, nutritional status and health of the women engaged in the sector, and whether aquaculture interventions can promote gender equality and women's empowerment. Women still face significant economic, social and cultural barriers that affect their participation in aquaculture, their access to, and control over assets and resources, and the income and benefits derived from these activities (Johnson et al., 2016; Kruijssen et al., 2018; Morgan et al., 2017; Phillips et al., 2016; Ramírez & Ruben, 2015). The lack of disaggregated data from aquaculture interventions and their evaluations have prevented researchers from capturing important learning for policy and practice, including the ability to assess whether cultural norms reduce or prevent women from reaping the benefits of aquaculture or the circumstances in which the design and implementation of aquaculture interventions can have positive impacts around women's empowerment.

Aquaculture is a sector with potential in several areas of international development, and while there is still limited evidence regarding its impact, synthesising the literature available becomes an increasingly relevant task for programme and policy making. With this review we aimed to fill this gap by bringing together existing evidence and exploring, with a gender lens, the impact of aquaculture on productivity, income, nutrition and women's empowerment.^1^

### 2.2 The intervention

The strategic rationale for promoting aquaculture is underpinned by the realisation of expected direct and indirect improvements in development outcomes for individuals, households and communities. Within the review, we have explored aquaculture interventions in low‐ and middle‐income countries that aim to increase productivity, income, nutrition and women's empowerment. We adopted a broad definition of aquaculture, including all types and scales of aquaculture activities to explore its impact along the value chain. We have explored the impact of aquaculture interventions on four broad components: productivity, income, nutrition and women's empowerment.

We follow FAO and refer to aquaculture as the “farming of aquatic organisms including fish, molluscs, crustaceans and aquatic plants in inland and coastal areas. Farming implies some form of intervention in the rearing process to enhance production, such as regular stocking, feeding and protection from predators. Farming also implies the individual or corporate ownership of the stock being cultivated” (FAO, 2020a, p. 23).

In this review, we defined “aquaculture interventions” as any project, programme, or policy aiming to provide new and/or improved activities at any stage of the aquaculture value chain. Therefore, we included interventions in all types of aquaculture operations regardless of their scale: from small‐ to medium‐ and large‐scale with respect to land size, use of hired labour, capital investment, and level of technological sophistication. In this, we follow Philips et al. (2016), and acknowledge that definitions based on the scale of the operations are not agreed upon and may have different meanings in different countries and regional contexts. For example, a portion of the literature refers to “small‐scale aquaculture”, referring generally to farming that uses low‐input methods and where a large percentage of farm labour is provided by household members. Hence, while we discuss and analyse definitions and scales of aquaculture operations whenever possible, we aimed to map the evidence around the whole sector.

For the review, we covered different types of aquaculture systems. A key difference exists, for example, between land‐ and water‐based aquaculture. Both systems require access to either land or water bodies, which might represent a barrier to engaging in aquaculture activities, especially when ownership or access is not free, or is regulated or precluded to some individuals based on their socioeconomic status. Land‐based systems are more common and usually stock fish in rice fields and ponds on dry land. Water‐based systems involve stocking fish in pens or cages directly in enclosures or attaching them to substrates in coastal or inland waters such as rivers or bays (Halwart et al., 2000). Land‐based aquaculture requires ownership or access to land, while water‐based aquaculture require access to water bodies, which might or might not be free or regulated. When water is accessible, this is often the only aquaculture option for households or individuals with no land or no access to it. Therefore, when access is provided or free, water‐based systems may provide an entry point for landless people and poor fishers to farm fish (Edwards, 2000).

We included interventions that affect aquaculture along its value chain, covering activities related to input supplies and services, production and postproduction activities, such as processing, trading and marketing.^2^ These interventions are generally productivity‐focused, aiming to improve the quantity and quality of aquaculture production, with the ultimate goal of increasing the income generated from aquaculture activities. However, we considered aquaculture interventions that improve the efficiency of the sector as a whole and have either a productivity, income or market‐enabling focus. This could involve, for example, providing training or better access to inputs (such as feed, seed and fertilisers), or improving the use and uptake of technology and management practices.

At times, aquaculture interventions aim to combine better aquaculture production and practices with other social and cultural objectives. For example, interventions could also aim to improve community‐based support to aquaculture activities, while others could have additional objectives on nutrition knowledge and practices, or have a deliberate focus on gender equality and empowerment to promote a more equal participation of women in aquaculture and in society. In this review, we included all types of interventions and highlighted when they have any additional social or cultural components. Whenever possible, we included and looked at the impact of aquaculture interventions on productivity, income, nutrition and women's empowerment, as well as the potential additional impact of adding other intervention components on these outcomes. For this purpose, we expected extra components to mostly fit into these two categories:

- Nutrition and behavioural change interventions, which aim to improve awareness and knowledge of the nutritional benefits of healthy diets; for example, emphasising the importance of including fish and other aquatic organisms in diets, especially among pregnant women and children.
- Gender equality and women's empowerment interventions that aim to support and promote women's equal access and participation in the sector.

### 2.3 How the intervention might work

Aquaculture can be a vehicle for improving livelihood and nutrition in low‐ and middle‐income countries. Aquaculture interventions can play a key role in enhancing or accelerating its impact and to ensure the equal distribution of benefits. In this section, we explore four impact pathways through which aquaculture interventions could help deliver benefits along the aquaculture value chains, in terms of productivity, income, nutrition and women's empowerment.

**Figure 1:**
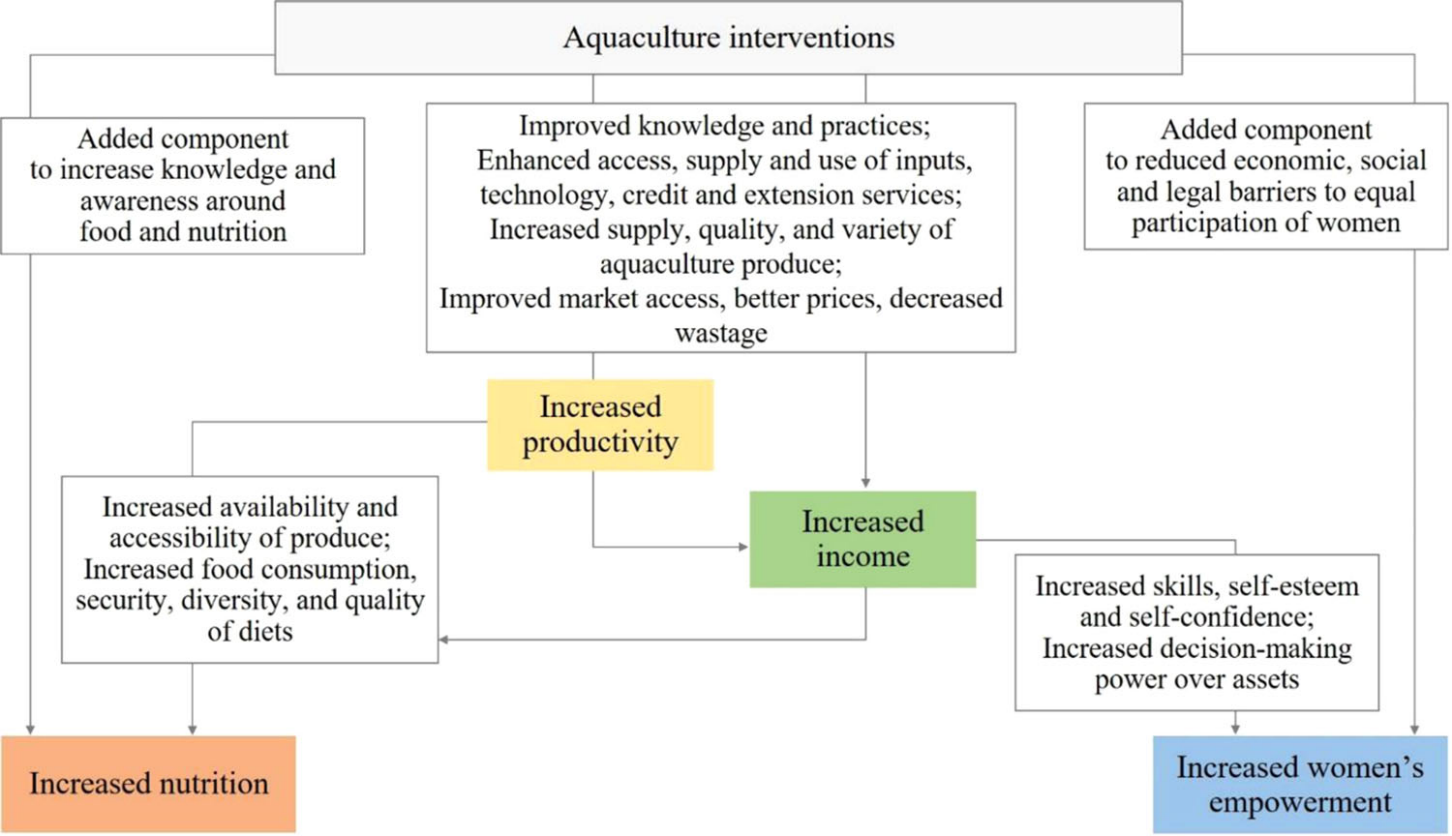
Theory of change

For this review, we used a theory of change that captures the outcomes and mechanisms that apply to a number of generic aquaculture interventions to maintain a clear focus on the key domains: productivity, income, nutrition and empowerment. Figure 1 shows a graphical representation of the theory of change, which distinguishes between main outcomes and intermediary outcomes for these four domains. This section provides a narrative description of the expected pathways to impact, followed by a review of the existing literature on each of them.

Existing literature suggests that aquaculture interventions would have an impact on key domains of productivity and/or income. Based on Dey and Ahmed (2005), aquaculture production can be increased through at least four pathways: more efficient use of farmers' resources and of existing inputs and technology, the development of new technologies and the transfer of these to farmers, an increase in the use of inputs, and an increase in the area dedicated to fish production. The local environmental and socioeconomic constraints will determine which options are more feasible or likely to be more effective in a specific context, and different aquaculture interventions might therefore focus on one or a combination of the above. Moreover, while interventions might have additional social objectives, we expected the main objective of an aquaculture intervention to be to improve production and productivity within the sector so as to generate and ensure a new or higher source of income and more sustainable livelihood. If this was met, we then also expected aquaculture to generate positive effects on other domains, such as nutrition and women's empowerment. For example, if productivity of a small fish farmer increases, the farmer can get a higher income by selling more fish to the market or by producing food that ensures better diets for his/her family. When the fish farmer is a woman, and aquaculture generates new or extra skills and income, this can potentially have a positive effect on her self‐esteem, self‐confidence, and her role within the household and beyond.

Depending on the specificity of the intervention, productivity and/or income outcomes can be achieved through an increase in some of the following intermediate outcomes: improved access, supply, and use of inputs, technology, credit and extension services or improved aquaculture knowledge and practices, such as better pond management or marketing practices. These may also lead to an increase in the quantity produced, less waste, or an increase in the variety or quality of the aquaculture production. Overall, while interventions might affect these outcomes to a different extent, the ultimate impact will be a more efficient market system, more production, higher productivity and overall a higher return from engaging in aquaculture. This higher return can take different forms: more aquaculture products to be consumed at home, more income derived from selling aquaculture produce, or more employment opportunities and therefore higher wages in the sector.

The next domain of interest is related to nutrition, addressing how more productivity or income in aquaculture affects the nutrition and health of those involved in aquaculture, and if interventions designed with an explicit nutritional component generate a higher impact on nutrition than productivity‐ or income‐focused aquaculture interventions. Through increasing production, productivity or income, aquaculture interventions may make fish and aquaculture more accessible and affordable. This alone could have an impact on food security and on the quantity and quality of nutritious food that household members could enjoy, which in turn, could improve their general health status. This impact would be amplified if the interventions come with additional activities that effectively raise the level of knowledge and awareness on the importance of food and nutrition for health. Whenever behaviour and educational components are incorporated and carried out as part of the intervention package, the impact on nutrition outcomes and on other related outcomes such as nutrition knowledge and awareness may be amplified.

Similarly, if aquaculture interventions affect the level of production, productivity or income of female individuals engaged in the sector, this may have a positive impact on a number of outcomes related to women's participation and benefits from aquaculture activities, with a potentially positive contribution towards empowerment. Social and cultural norms tend to act as barriers for women and reduce their participation in aquaculture production activities and eventually the return they get from it. When aquaculture interventions are designed and carried out with a gender equality lens, it may help improve the way in which women participate in the sector, the return they get from their participation, and the skills they experience and develop. More opportunities to gain skills and income is more likely to translate into having more productive resources that can help put women more in control of their decisions, thus improving their roles in their household and beyond. While the ultimate outcome is women's empowerment we appreciate that empowerment is a process as much as an outcome.

#### 2.3.1 Productivity and income

Conceptually, aquaculture interventions that aim to increase production and productivity of aquaculture activities, have both direct and indirect benefits on income, livelihood and poverty. The linkages and pathways are similar to the ones developed in agriculture economics and are discussed extensively for the aquaculture sector (see Ahmed & Lorica, 2002; Rashid et al., 2019; Toufique & Belton, 2014). For example, Toufique and Belton (2014) define the following four linkages: direct consumption links (increased consumption from own production), indirect consumption links (increased availability and accessibility of fish), direct income links (increased income for aquaculture producers), and indirect income links (employment in the fish value chain and consumption linkages).

The income linkage is based on the assumption that aquaculture interventions, by improving efficiency along the value chain, can generate higher returns and therefore higher incomes for the farmers involved. Some interventions would affect more specifically the productivity side of aquaculture operations, while others would focus on the aquaculture market. We expected most interventions to be productivity‐focused and affect income via an increase in production and productivity; however, some market‐oriented interventions may also affect revenues and income directly, not necessarily via productivity, and we specifically allowed for this pathway in our theory of change. Either way, there can be an impact on individuals and households involved, and if aquaculture engages poor households, this could have a direct impact on their incomes and on their poverty status. Moreover, aquaculture growth can have an impact on employment opportunities, and more generally on economic growth, thus benefiting communities beyond the individuals engaged in aquaculture.

From a consumption side, increase in availability and accessibility of aquaculture produce might have an impact on prices, which would affect the consumers' ability to buy fish and other aquaculture produce (whether they are producers or not) and, thus, increase real incomes. The overall impact on the economy and poverty would be an empirical matter and would depend on who are the aquaculture producers (poor vs. nonpoor), who consumes fish and how consumption responds to possible changes in prices, and to the overall magnitude of the direct and indirect effects on the economy and poverty.

Studies highlight how the distributional impact of aquaculture could even be negative if the poor cannot reap the benefits of aquaculture or if the benefits are mostly concentrated in the hands of a few large better‐off producers. For example, whenever aquaculture requires a minimum level of access to land, technology and resources, the poorest, often landless households, will not be likely to benefit from it (Ahmed et al., 1995; Kassam & Dorward, 2017).

Empirical studies that help quantify the specific linkages and provide an overall impact of aquaculture interventions on income and poverty of different types of households are still quite limited. While studies have often found correlations between aquaculture activities and poverty, it is harder to make attribution claims if studies are not designed with the specific objective of assessing the impact of aquaculture on the overall consumption and welfare status.

Kassam and Dorward (2017), investigated the poverty impacts of pond and cage aquaculture in Ghana, and Rashid et al. (2019), analysed aquaculture production and its impact on prices, consumption, income for different types of households in Bangladesh. Both studies found that aquaculture had a positive impact on the economy and contributed to a reduction in poverty levels in their countries. Kassam and Dorward (2017) found that the overall impact occurred mostly via the indirect effects on economic growth of nonpoor farmers, while Rashid et al. (2019) found that an increase in production benefited all producers (who are both poor and nonpoor) and that the reduction in prices benefited all populations, in particular poorer households, thus generating a substantial positive impact on the country's poverty level.

On one hand, Kassam and Dorward (2017) aimed to assess the poverty impacts of small‐scale pond aquaculture and small‐medium enterprises (SME) cage aquaculture in Ghana, and to compare the relative significance of the direct impacts on poor small‐scale fish farmers and the indirect impacts on economic growth and employment from SMEs. They found that nonpoor small‐scale pond fish farmers who have been trained and/or use better management practices hold the most potential to impact poverty indirectly through generating economic growth. These indirect impacts are higher than the direct impacts on poor small‐scale fish farmers and the indirect impacts from SMEs. In turn, Rashid et al. (2019) found that the impacts of aquaculture growth on income distribution and poverty reduction in Bangladesh have been substantial, with aquaculture explaining almost 10% of the overall poverty reduction in Bangladesh during the first decade of the 21st century. Bangladesh experienced a rapid growth in the demand of aquaculture fish since 1980s, but its supply increased even more rapidly, resulting in a decline in real price. The growth in production led to higher incomes for producers but also lower prices for consumers, which includes to some degree the producers as they also consume fish. This in turn translated into increased consumption for all types of households, in particular for the bottom two income quintiles, income gains for all households, particularly in aquaculture producers, and an overall substantial reduction in the proportion of households below the poverty lines.

Overall, while the literature suggests that aquaculture has the potential to positively impact the poorest households, the empirical evidence is quite scarce and nuanced to inform the contexts in which we can expect this impact. More quality studies and evaluations of aquaculture interventions are needed to help inform how the income and poverty impact can be promoted effectively and equitably.

In this systematic review, we brought together studies that explore how aquaculture interventions affect production, productivity, income, market and prices. We explored how effective aquaculture interventions are, and for whom they work best.

#### 2.3.2 Nutrition, health and food security

Whenever aquaculture interventions succeed to promote greater quantity or higher quality aquaculture production that translates into better quality consumption, it follows that there may also be an impact on nutrition and food security among individuals engaged in aquaculture and, more generally, for the entire country. It is important to acknowledge that nutrition is a long‐term and complex phenomenon determined by many factors, which are often beyond the control of a specific intervention. When designing and evaluating an intervention, it is thus important to measure outcomes that are realistic and proportionate. While anthropometric measures tend to be long‐term objectives, changes in food consumption and improving quality of diets are considered important and achievable outcomes, whether or not they later translate into impacts on other nutrition measures.

Conceptually, the impact pathways on nutrition can occur via two main mechanisms. First, an increase in quality of diets can occur due to an increase in their own consumption when aquaculture farmers produce more quantity and quality of nutritious food and keep some of it for their personal consumption. Second, an increase in the consumption of nutritious food from aquaculture could occur as a result of an increase in real incomes. Higher incomes from aquaculture could lead to more resources to buy more or better food at the market and, therefore, have an impact on nutrition and quality of diets.

The impact on nutrition via the second mechanism affects all households in a community, whether they are involved in aquaculture or not. If aquaculture interventions lead to more accessible aquaculture produce in the economy, real incomes increase even for households not engaged in aquaculture. Hence, all consumers could afford a more nutritious food basket and receive the associated dietary benefits.

The link between higher income and nutrition is well‐established in the literature and earlier studies on agriculture identified that increasing household income is a particularly important factor to improve dietary intake, as the consumption of nonstaple foods is positively related to increases in income (Hawkes & Ruel, 2006; Leroy & Frongillo, 2007; World Bank, 2007). Though there is a paucity of research on the impact of aquaculture on nutrition, useful insights can be drawn from the broader agriculture literature, which sometimes also includes aquaculture interventions. Studies tend not to be able to separate out the two mechanisms and tend to measure the overall effect on the consumption.^3^

Relevant studies on nutrition have found that agriculture can lead to an increase in consumption, in particular, for the food item targeted by the intervention, but the impact on nutrition is more unclear. Ruel and Alderman (2013) used a similar framework to our review when examining the literature on home gardens and homestead food production systems. The authors found that there is little evidence of effectiveness of homestead food production programmes on maternal or child nutrition status (i.e., anthropometry or micronutrient status), with the possible exception of vitamin A status. Moreover, they found that the nutritional effect is more likely when agriculture interventions target women and include women's empowerment activities, such as improving their knowledge and skills through behaviour‐change communications or promoting their increased control over income from the sale of targeted commodities. An update of this review (Ruel et al., 2018) looked at more recent literature and found that nutrition‐sensitive agricultural programmes showed positive impacts on dietary diversity, food consumption targeted by the programmes, and micronutrient intake. Unlike the first review, these findings were consistent across different contexts and types of interventions. In addition, a review by Masset et al. (2012) of the impact of agriculture interventions (mostly home gardens) on nutrition found that most studies reported a positive effect on food consumption. Depending on the interventions, they found an increase in the consumption of the food item targeted by the intervention (more fish consumption for aquaculture interventions, more dairy products for dairy interventions, and so forth) but little evidence was available on changes in the diet, micronutrients' intake, and children's nutritional status. Similarly, Bird et al. (2019) reviewed the impacts of agriculture interventions on nutritional outcomes in South Asia and found no convincing evidence of an impact of agricultural interventions on child anthropometric measurements. One study included in the review (Pant et al., 2014) looked specifically at the impact of aquaculture interventions on nutrition in Bangladesh. The authors found that, compared to baseline, households increased their monthly consumption of fish, meat and eggs, and increased annual household income. Similar increases in consumption were found by Kawarazuka (2010), who looked specifically at the impact of pond‐based aquaculture on dietary intake/nutritional status.

Taken together, these studies suggest that agriculture interventions can lead to more consumption, especially for the food item targeted by the interventions. However, this increased consumption might or might not translate into a measurable impact on nutrition. Masset et al. (2012) attributed the lack of evidence on nutritional status to the methodological weaknesses of the studies reviewed, rather than to a lack of impact, and called for more research on the topic. These studies also highlight the importance of measuring nutrition outcomes such as diversity and quality of diets. These are identified as key outcomes of interest when assessing the impact of interventions given that nutrition and under‐nutrition are complex phenomena determined by multiple causes and often beyond the household or intervention's control.

With this review, we brought together and analysed the studies that look specifically at aquaculture with the aim to shed some light on whether and how aquaculture interventions can be effective at promoting better quality food consumption that translates into better nutrition and health.

#### 2.3.3 Aquaculture and women's empowerment

SDG5 puts gender equality and empowerment of women and girls on top of the development agenda. Women should be able to enjoy effective participation, equal opportunities in political, economic, and public life decision‐making, and equal rights to benefit from economic resources.

The extent to which aquaculture interventions contribute to empower women and girls is unclear.^4^ Conceptually, to the extent that aquaculture engages women in new and/or more productive economic activities, aquaculture has the potential to expand their choice, strengthen their voice and increase the importance and role of women within the household and the community. Aquaculture could provide a means for women to generate more income for themselves and their families, as well as acquire and develop knowledge and skills. This could lead to having more voice, respect and control over her and her household decisions.

Johnson et al. (2018) provide a useful framework to distinguish between impacts of interventions on female empowerment and identify three main approaches: reaching women, benefitting women and empowering women. An intervention focusing on reaching women emphasises engaging women in project activities and tracks progress in terms of participation, for example, measuring the number of women who attend meetings or receive training. In an intervention focused on benefitting women, the focus is on ensuring that the outcomes the project is seeking—for example, reduced hunger, increased income, or greater resilience—are captured by women. Empowering women involves strengthening their ability to make strategic life choices and to put those into action.

Evidence from agriculture show that even when interventions lead to improvements in women's agricultural production, income or nutritional status, they rarely succeed in reducing underlying inequities between men and women (Johnson et al., 2016, 2018; Quisumbing et al., 2013; Santos et al., 2014). Following Johnson et al.'s (2018) framework, while increasing the income that women earn would be considered “benefiting” women, if women do not have increased control over how this income is managed or used, an intervention would not be “empowering” women.

Despite the importance of the sector, and the interest around what works to promote women's empowerment, the literature on aquaculture and gender is scarce. Evidence is limited on the quality of female participation and the economic returns from aquaculture. Additionally, the lack of sex‐disaggregated data is an issue often highlighted in the literature as it reduces the potential for gender analysis of the sector, which is the basis for the development of gender sensitive policies and planning (FAO, 2014a, 2020b; Harper et al., 2013; Kruijssen et al., 2018; Weeratunge et al., 2010).

Economic, social and cultural barriers affect the participation of women to the sector, their access and control over assets and resources, and the income and benefits they derive from the activities they perform (Johnson et al., 2016; Kruijssen et al., 2018; Morgan et al., 2017; Ramírez & Ruben, 2015). Below we discuss some of these barriers and, more generally, the social norms and cultural dynamics that affect women's position in the sector.

Kruijssen et al. (2018) put together the most comprehensive review on aquaculture and gender to date and find gendered imbalances along different dimensions (including division of labour, distribution of benefits, access and control over assets and resources, gender and social norms, power relations and governance), arguing that these formal and informal barriers, including gender norms, would limit women's equal engagement and returns. In addition, women face unequal access to aquaculture as they tend to have less access and control over assets, including a disadvantage in ownership and control of land or ponds (Ndanga et al., 2013; Veliu et al., 2009). For example, female farm ownership is 2%–3% in Vietnam (Veliu et al., 2009), female pond ownership is <1% in Bangladesh (Khondker et al., 2010), and women tend to have less access and control over capital (Ndanga et al., 2013), skills, technologies and extension services (Morgan et al., 2017).

When women participate in aquaculture labour activities, their roles vary significantly across countries and production nodes, so it is not appropriate to generalise; however, the benefits they get are often less than their male counterparts. Nevertheless, FAO (2020b) highlights that women play an important role throughout the value chain, providing labour in both commercial and artisanal fisheries and identifies small‐scale production, postharvest industrial and artisanal processing, value addition, marketing and sales as the most common roles for women in aquaculture. Evidence suggests that women tend to receive lower returns and are disproportionately represented in less‐profitable nodes of aquaculture value chains (Kruijssen et al., 2013) or where jobs are regarded as especially insecure (Kruijssen et al., 2018; Veliu et al., 2009). For example, a case study on Cameroon found that women find it challenging to combine domestic workload with aquaculture activities and prefer activities that could be undertaken in evenings or in spare moments over those that required dedicated, daily supervision (Brummett et al., 2011). In Kenya, when fish processing became profitable, men replaced women who first had those jobs (Ndanga et al., 2013). Lastly, a study from Chile showed that women faced no cultural barriers to their entry in the growing aquaculture job market; however, access to jobs in the sector did not come with equal returns and the study found salary differences in favour of men, as a result of gender discrimination (Ramírez & Ruben, 2015).

Overall, evidence suggests that social norms and cultural dynamics significantly affect and shape women's participation and return from aquaculture (Morgan et al., 2017; Ramírez & Ruben, 2015), affecting women's capacity to adopt and retain aquaculture technologies (Morgan et al., 2017) or to translate economic returns into more empowerment (Sari et al., 2017). In Bangladesh, one study found key gender differences in the division of labour, in the levels of decision‐making power, and in access to and control over resources and benefits from aquaculture, identifying that these differences are rooted in and perpetuated by social and gender norms and relations (Kruijssen et al., 2016).

In order for aquaculture interventions to have an effect on improving gender equity or promoting empowerment, they need to take into account the specific social norms of the context they operate in and the barriers they create for women. Interventions need to be targeted and realise the importance of addressing underlying social and gender norms. While addressing underlying social and gender norms is likely to be beyond the aim of any individual aquaculture intervention, positive contributions in this direction can be made through awareness training and community support, giving explicit attention to gender‐based constraints, access and control over resources, decision‐making power, and gender norms (Kruijssen et al., 2016; USAID, 2013).

### 2.4 Why it is important to do this review

There has been an advocacy for aquaculture research and production guidelines for decades (Pullin & Shehadeh, 1980). Aquaculture production has continued to develop since the 1980s, reaching a record high in 2018 after having doubled in the past 20 years in Asia and Africa. More importantly, aquaculture is projected to supply more than half of the world's fish‐based food by 2030, and then take over future fish sourcing (World Bank, 2013).

This steady increase in production has been in line with investment and research efforts from government agencies, international organisations and academic centres, which have continued to promote aquaculture as a sustainable option to feed the world's growing population. The following are examples of recent aquaculture programmes that reflect the extent of these efforts.

The Global Environment Facility (GEP) provides funding to developing countries and countries with economies in transition to help them meet the objectives of international environmental conventions. In the last 5 years, GEP has supported government programmes in Bangladesh, Chile, Malawi, Myanmar and Timor Leste to make their aquaculture activities more climate change resilient, adding up to almost USD 23 million (GEP, n.d.).

In 2012, the Aquaculture for Food Security, Poverty Alleviation and Nutrition (AFSPAN), an EU‐funded, 3‐year project coordinated by FAO was created to understand the link between aquaculture and food security. With a EUR one million budget, the project was implemented in 11 developing and low‐income, food‐deficit countries. AFSPAN concluded that aquaculture contributes significantly to food security and nutrition, as well as to other outcomes such as job creation, income generation, and women's empowerment (CORDIS, 2015).

Under the Feed the Future multiyear strategy, the United States Agency for International Development has supported two aquaculture programmes in Bangladesh. The first project, Aquaculture for Income and Nutrition (AIN), was implemented by WorldFish between 2011 and 2016 with a USD 25 million budget. AIN aimed to increase aquaculture quality production, improve the nutrition and income status of farm households, promote commercial aquaculture, and support capacity building of the public and private sector (Keus et al., 2017). Building on the success of AIN, a second programme is being implemented, the Bangladesh Aquaculture and Nutrition Activity. Starting in 2018, this 5‐year and USD 24.5 million project intends to develop a more inclusive sector by strengthening the aquaculture market systems and a nutrition‐based behaviour with special focus on women and youth (WorldFish, n.d.).

The increase in aquaculture production and fish‐based food consumption, coupled with the challenges that climate change is posing to the sustainability of our diets, to which aquaculture might represent a solution, provide a timely backdrop for an up‐to‐date review of the impact of aquaculture interventions on productivity, income, nutrition, and women's empowerment to contribute to policy and programming in the sector. While there is some relevant literature on agriculture and its impact on nutrition, few quality studies exist specifically on aquaculture. Moreover, despite the increasing importance of aquaculture, to our knowledge no effort has been made to draw insights from how best to design and implement aquaculture interventions when income, nutrition and women's empowerment are the key objectives.

While there are a number of relevant existing reviews, our review differs in two key ways. First, it is the first review with a specific focus on aquaculture interventions. Second, we explored the literature from a gender lens. Previous reviews, detailed below, looked at either the broader agricultural sector, which included none or only few aquaculture interventions (Bird et al., 2019; Masset et al., 2012; Ruel et al., 2018) or covered aquaculture under a narrow scope (Gambelli et al., 2019; d'Armengol et al., 2018).

The systematic review led by Bird et al. (2019) looked at peer‐reviewed studies published between 2012 and 2017, detailing impacts of household‐ or farm‐level agricultural interventions on nutritional outcomes in South Asia. The authors identified six intervention studies and found mixed evidence of impact. Interventions had a positive impact on intermediate outcomes on the pathway from agricultural intervention to nutritional or health status, including dietary quality and dietary diversity of households and individuals. The evidence on the impact on final nutritional outcomes was mixed: one paper reported that home gardens with poultry reduced the odds of anaemia, but there was no convincing evidence of an impact of agricultural interventions on child anthropometric measurement, as reported in four papers.

Masset et al. (2012) conducted a systematic review of the evidence around effectiveness of agricultural interventions (including biofortification, home gardens, small scale fisheries and aquaculture, dairy development, and animal husbandry and poultry development) aiming at improving the nutritional status of children. The review included 23 studies, mostly evaluating home garden interventions. The authors found that the interventions had a positive effect on the production of the agricultural goods promoted, but not on households' total income. The interventions were successful in promoting the consumption of food rich in protein and micronutrients, but the effect on the overall diet of poor people remains unclear. The evidence reviewed showed no effect of these interventions on nutritional status of children, but methodological weaknesses of these studies cast serious doubts on the validity of the results. The authors attribute this to the lack of statistical power of the studies reviewed rather than to the lack of effectiveness of the interventions.

Ruel et al. (2018) reviewed the evidence related to nutrition‐sensitive agriculture programmes from 2014 onwards, including 16 impact evaluations and 28 observational studies. The authors found that all programmes were highly successful at both meeting their production and consumption targets, and at providing households with access to nutrition‐rich foods. However, none of the impact evaluations identified in the review covered aquaculture interventions.

On the other end of the spectrum, some reviews had a narrow scope that shed lights on specific aspects of the aquaculture sector. d'Armengol et al. (2018) focused particularly on small‐scale fisheries with a comanagement structure and component. The authors included 70 studies and found that comanagement delivers both ecological and social benefits, as it increases the abundance and habitat of species, fish catches, actors' participation, and the fishery's adaptive capacity, as well as induces processes of social learning. In turn, Gambelli et al. (2019) brought together studies in the field of the economic dimension of organic aquaculture. The authors found that profitability in organic aquaculture is not guaranteed for all aquaculture species, and that the feed and other fixed costs can be an issue if these are not balanced by adequate price premiums.

Moreover, while none of the existing reviews explored the impact on aquaculture from a specific gender perspective, one review focused on gender issues in aquaculture. Kruijssen et al. (2018) reviewed the evidence on gender relations in aquaculture value chains by looking at the gender division of labour, distribution of benefits, access and control over assets and resources, gender and social norms, and the power relationships within and outside the chain. The review showed that there is limited high quality sex‐disaggregated data regarding aquaculture value chains. Existing evidence, however, indicates gendered imbalances in all the dimensions assessed, with women's equal engagement and returns being limited by formal and informal barriers.

With the present review, we intended to provide an up‐to‐date review of existing evaluation studies that explore the impact of aquaculture interventions on productivity, income, nutrition, and women's empowerment to fill the existing gaps on the impact of aquaculture and its gender dynamics.

## 3 OBJECTIVES

This review examined and synthesised the state of the evidence around what works to improve productivity, income, nutrition, and women's empowerment outcomes of households involved in aquaculture in low‐ and middle‐income countries.

We were particularly interested in addressing the following research questions:

1. Do aquaculture interventions increase the productivity, income, nutrition and empowerment of individuals engaged in aquaculture and their households in low‐ and middle‐income countries?
2. Do aquaculture interventions generate income and nutrition spillover effects beyond the farmers' households?
3. To what extent do the effects of aquaculture interventions vary by intervention type, population group and location? In particular, to what extent do effects vary by gender?
4. What are the potential barriers and facilitating factors that impact the effectiveness of aquaculture interventions?
5. What is the cost‐effectiveness of different aquaculture interventions focused on productivity, income, nutrition and empowerment outcomes?

## 4 METHODS

As planned in the protocol for this review, we have followed the Methodological Expectations of Campbell Collaboration Intervention Reviews (MECCIR) Conduct and Reporting Standards (2019a, 2019b) and our process was based on recognised guidelines for systematic reviews of effectiveness in international development (Waddington et al., 2012).

To address research questions 1–3, we synthesised evidence provided in impact evaluation studies and, whenever possible, analysed its corresponding effect size data. This allowed us to provide estimates of average effects and heterogeneity of reported changes in outcomes measured within the pathways described in the theory of change.

To capture evidence on the context, implementation and underlying mechanisms, we also adopted a mixed‐methods, theory‐based approach to address research question 4. Under the “effectiveness+” framework (Snilstveit, 2012), we searched for and synthesised supplementary evidence, including information derived from intervention documents, process evaluations, formative assessments or similar documentation.

Finally, to address research question 5, we have searched and synthesised cost data for the interventions of interest drawing on standard approaches to synthesise economic appraisal evidence (Shemilt et al., 2008).

### 4.1 Criteria for considering studies for this review

#### 4.1.1 Types of studies

To address research questions 1–3, we included evaluations that use an experimental or quasi‐experimental design (QED) to robustly measure a change in outcomes that is attributed to an intervention as is compared to an appropriate counterfactual. We have included randomised studies and nonrandomised studies as described below.

*Randomised controlled trials (RCTs)*

- RCTs, with assignment at individual, household, community or other cluster level, and quasi‐RCTs using prospective methods of assignment such as alternation.

*Nonrandomised studies*

- Regression discontinuity designs, where assignment is done on a threshold measured at pretest, and the study uses prospective or retrospective approaches of analysis to control for unobservable confounding.
- Studies using design or analytical methods to control for unobservable confounding, such as natural experiments with clearly defined intervention and comparison groups, which exploit natural randomness in implementation assignment by decision makers (e.g., public lottery or random errors in implementation), and instrumental variables estimation.
- Studies with pre‐ and postintervention outcome data in intervention and comparisons groups, where data are individual level panel or pseudo‐panels (repeated cross‐sections), which use the following methods to control for confounding:
  – Studies controlling for time‐invariant unobservable confounding, including difference‐in‐differences, or fixed‐ or random‐effects models with an interaction term between time and intervention for pre‐ and postintervention observations.
  – Studies assessing changes in trends in outcomes over a series of time points (e.g., interrupted time series [ITS]), with or without contemporaneous comparison (e.g., controlled ITS), with sufficient observations to establish a trend and control for effects on outcomes due to factors other than the intervention.
  – Studies which control for observable confounding, including nonparametric and parametric approaches:
    o Nonparametric approaches, for example, statistical matching, covariate matching, coarsening, propensity score matching.
    o Parametric approaches, for example, propensity‐weighted multiple regression analysis.

While we also considered evaluations of pilot studies aimed to be scaled up, efficacy studies, feasibility studies, acceptability studies, literature reviews and systematic reviews were not included as primary studies.

To address research question 4, we included a broad range of evidence, sourced from searching for additional documentation on the programmes covered by the included papers, such as design documents, monitoring and evaluation reports, primary research, and other documentation related to the implementation of these interventions.

To assess the relative cost‐effectiveness of interventions from included studies, as stated in research question 5, we have considered relevant documentation on these economic evaluations. This included evidence on unit or total costs to implementers, participants and nonparticipants as relevant, with the aim to compare data across interventions.

#### 4.1.2 Types of participants

The unit of analysis considered for this review included individuals, households, villages, municipalities, or community‐based organisations. The study samples were based in low‐ and middle‐income countries in accordance with widely used international classifications (World Bank, n.d.). We anticipated that studies would mainly focus on people living in rural areas; however, studies in which participants live in peri‐urban or urban areas were also eligible. Participants considered could be of any age, and there were no restrictions based upon any other demographic characteristics.

#### 4.1.3 Types of interventions

To understand potential differences between aquaculture interventions and to capture the role of women across these activities, we applied a broad definition of interventions. We included any project, programme, or policy seeking to provide new and/or improved aquaculture activities in any of the various stages of its value chain, including input supplies and services, production, processing, trading or marketing. For example, this could include activities related to farming fish and other aquatic organisms (e.g., seaweed), based on ponds, cages, and other aquaculture systems, involving land‐ and water‐based aquaculture for which there is relevant evidence.

The majority of aquaculture production activities are conducted by small scale farms, owned or managed by families (FAO, 2014b). Hence, we anticipated that included studies would focus on smallholder farming interventions. However, we did not exclude studies if their focus was on larger scale aquaculture activities.

Finally, for the review we have included any type of programme that promotes aquaculture in low‐ and middle‐income countries, which might also include one or a combination of aquaculture efficiency‐focused interventions, behavioural change interventions, capacity and skill development interventions, and gender equality and women's empowerment interventions.

#### 4.1.4 Types of outcome measures

##### Primary outcomes

To address research questions 1–3, we have focused on four groups of primary outcomes: productivity, income, nutrition and women's empowerment. Because the scope for this review was rather broad, we were open to map any measure related to these main groups, including some examples presented below.

The first group of outcomes related to the production, productivity, and market aspects of aquaculture activities. Examples of outcomes of interest for this group included prices of aquaculture production, measures of supply, accessibility and quality of inputs (such as seeds or fertiliser), access to markets, use of technology, or management practice.

The second group of primary outcomes related to the income of individuals engaged in aquaculture and their households. We were interested in, for example, the amount of income derived from aquaculture activities, the ratio of income derived from aquaculture on the total income, and expenditure measured at the individual or household level. Other relevant welfare outcomes referred to poverty (using income or consumption poverty measures) or other multidimensional poverty or livelihood measures.

The third group, nutrition outcomes, related to quantity, quality and diversity of the diet and health status of the participants and their households. Following the literature, we anticipated measures of these outcomes using food consumption levels or, to better capture quality, food security or food diversity scores, such as the Household Dietary Diversity Score (Swindale & Bilinksy, 2006). As nutrition measures, we included anthropometric measures, such as body mass index (BMI) for adults and weight‐for‐height, height‐for‐age and weight‐for‐age for children. Additionally, we were also interested in changes in knowledge and awareness on nutrition and quality of diets, and other health related indicators.

The fourth group of primary outcomes was related to the empowerment of women engaged in aquaculture activities. These measures generally look at whether and to what extent women have control over a number of dimensions as a proxy for their empowerment and control over their lives, including income from aquaculture (from an involvement in any of the stages of its value chain), household consumption and spending decisions. Outcomes of interest for this group included measures of confidence and trust in the community, equal participation along the aquaculture value chain, reduced wage gap, changes in attitude towards women, or established tools such as the Women's Empowerment in Agriculture Index (IFPRI, 2012).

##### Secondary outcomes

Reported outcomes from included studies that did not fall under any of the four main groups of outcomes of interest but were measured under relevant designs, were not excluded from the review. We coded and reported all relevant secondary outcomes (i.e., those that fall within the included population, intervention, and study design criteria) with the purpose of mapping the evidence around aquaculture interventions, including outcomes that we did not expect.

#### 4.1.5 Additional criteria

We have searched for relevant studies using the following additional criteria. We included studies published in any language, although we have developed search terms in English. Considering the intervention types and study designs defined for the review, we did not expect to identify relevant studies before 1980; hence, we have included studies with publication dates of 1980 or after. To minimise the potential of publication bias, we included studies regardless of their publication status; this covers studies identified in academic journals, books, institutional reports, conference proceedings, theses and dissertations, or organisational websites. We have also included studies with any length of follow‐up periods. Finally, we only included studies focused on low‐ and middle‐income countries; without having imposed any additional location restrictions for our review.

### 4.2 Search methods for identification of studies

#### 4.2.1 Electronic searches

We have searched for relevant studies on the following academic databases, organisational repositories, and agencies websites. To reduce the risk of publication bias, these information sources were selected to cover a range of publication types, including journal articles, working and discussion papers, conference proceedings, thesis and dissertations, and institutional reports. The review team documented the literature search process, including the search strategies adapted for each source.

*Academic databases*

- 3ie Development Evidence Portal: https://developmentevidence.3ieimpact.org
- British Library for Development Studies: https://guides.lib.sussex.ac.uk/c.php?g=655545%26p=4613793
- EBSCO (Agricola, AGRIS, CAB Abstracts^5^, Gender Studies Database, GreenFILE, IDEAS‐Repec, World Bank eLibrary): www.ebsco.com
- Econlit (Ovid): www.ovid.com/site/catalog/databases/52.jsp
- Scopus: www.scopus.com

*Grey literature sources*

- African Development Bank Group (AfDB): www.afdb.org/en/documents/publications
- Asian Development Bank: www.adb.org/what-we-do/data/publications
- CARE International: www.careevaluations.org
- Consultative Group on International Agricultural Research (CGIAR): https://cgspace.cgiar.org/handle/10568/83389
- ELDIS, Institute of Development Studies: www.eldis.org
- Food and Agricultural Organisations of the United Nations (FAO)—Fisheries and Aquaculture Department: www.fao.org/fishery/publications/search/en
- Foreign, Commonwealth and Development Office (FCDO): www.gov.uk/research-for-development-outputs
- Global Environmental Facility (GEF): www.gefieo.org/evaluations/all?f%5b0%5d=field_ieo_grouping%3A312
- Innovations for Poverty Actions (IPA): www.poverty-action.org/search-studies
- Inter‐American Development Bank (IDB): https://publications.iadb.org/en
- International Food Policy Research Institute (IFPRI): www.ifpri.org/publications
- International Fund for Agricultural Development (IFAD): www.ifad.org/en/web/ioe/evaluations
- J‐Poverty Action Lab (J‐PAL): www.povertyactionlab.org/evaluations
- OXFAM International: https://policy-practice.oxfam.org.uk/publications
- Overseas Development Institute (ODI): www.odi.org/publications
- Registry for International Development Impact Evaluations (RIDIE): https://ridie.3ieimpact.org
- Search4DEV: www.bibalex.org/Search4Dev/Category/subject
- United States Agency for International Development (USAID): www.usaid.gov/reports-and-data
- WorldFish: www.worldfishcenter.org/search/publications
- World Food Programme (WFP): www.wfp.org/publications
- World Health Organisation (WHO): www.who.int/publications

#### 4.2.2 Searching other resources

While systematic reviews and narrative literature review were not eligible for inclusion, we screened the reference lists of relevant reviews. These were identified by the search strategy or by the research team. Likewise, we have screened the reference lists of all included studies. Lastly, using Google Scholar, we also conducted a forward citation tracking for all included studies.

Additionally, we conducted a second search of references to address research questions 4 and 5 regarding factors that hinder or facilitate the effectiveness of aquaculture interventions and a cost‐effectiveness analysis of such interventions. This search focused on information related to the interventions covered by the included studies, in the form of supplementary documents, studies or reports including contextual information, cost data, process evaluations or similar documentation.

We undertook this search based on references to relevant documents within included papers, and using Google to search for by the programme name. When an intervention was clearly implemented and/or funded by a particular organisation, the organisation's website was also searched.

Once the screening process concluded and we had the list of included studies, we contacted the review's advisory group and published an institutional blog listing our included studies to try to identify additional records, either as included studies or as contextual documents of included interventions. We made every effort to contact authors from included studies to locate further contextual information as needed.

### 4.3 Data collection and analysis

#### 4.3.1 Description of methods used in primary research

Using the inclusion criteria set out in the previous sections, we anticipated that primary studies included in this review would use experimental or quasi‐experimental study designs and/or analysis methods to examine the extent to which changes in outcomes are attributable to the intervention. To this end, we have included randomised studies as well as nonrandomised studies that are able to suitably account for selection and confounding bias (Waddington et al., 2017).

#### 4.3.2 Criteria for determination of independent findings

Complex data structures are a common occurrence in meta‐analyses of impact evaluations. There are several scenarios through which these complex structures with dependent effect sizes might occur. For instance, there could be several publications that stem from one study, or several studies based on the same data set. Some studies might have multiple treatment arms that are all compared to a single control group. Other studies may report outcome measurements from several time points, or use multiple outcome measures to assess related outcome constructs. All such cases yield a set of statistically dependent effect size estimates (Borenstein et al., 2009). The research team assessed the extent to which relationships existed across the studies included in the review. We have made every attempt to avoid double counting of identical evidence by linking papers before data analysis. Where we have several publications reporting on the exact same effect, we have used effect sizes from the most recent publication. We have also utilised information provided in studies to support these assessments, such as samples sizes, programme characteristics and key implementing and/or funding partners.

We have extracted effects reported across different outcomes or subgroups within a study, and where information is collected on the same programme for different outcomes at the same or different periods of time, we extracted information on the full range of outcomes over time. Where studies report effects from multiple model specifications, we used the author's preferred model specification. If this is not stated or is unclear, we used the specification with the most controls. Where studies report multiple outcome subgroups for the same outcome construct, we calculated a “synthetic effect size” (Borenstein et al., 2009, ch. 24). Where studies report multiple outcomes or evidence according to sub‐groups of participants, we recorded and reported data on relevant sub‐groups separately. Further information on criteria for determining independent effect sizes is presented below.

We dealt with dependent effect sizes in one of two ways, either through the use of robust variance estimation (RVE; Fisher & Tipton, 2015; Hedges et al., 2010), or through data processing and selection techniques. RVE using a small sample adjustment was the preferred analytic method when feasible. The RVE approach allows us to use all available data in our effect size estimates, even data that is statistically dependent. However, these analyses must have >4 degrees of freedom to make valid inferences. In cases where analyses do not meet this criteria, data processing and selection techniques were used to deal with dependent effect sizes.

If RVE analyses were not feasible for a meta‐analysis of any given intervention or outcome group, we utilised several criteria to select one effect estimate per study. Where we had several publications reporting on the same study, we used effect sizes from the most recent publication. For studies with outcome measures at different time points, we followed De La Rue et al. (2013) and synthesised outcomes measured immediately after the intervention (defined as 1‐6 months) and at follow‐up (longer than six months) separately. If multiple time points existed within these time periods, we used the most recent measure. We anticipated many of the interventions included in the review would be ongoing programmes and the follow‐up would, therefore, reflect duration in a programme rather than time since intervention. When such studies reported outcome measures at different time points, we identified the most common follow‐up period and included the follow up measures that match this most closely in the meta‐analysis. When studies included multiple outcome measures to assess related outcome constructs, we followed Macdonald et al. (2012) and selected the outcome that appears to most accurately reflect the construct of interest without reference to the results. If studies included multiple treatment arms with only one control group and the treatments represent separate treatment constructs, we calculated the effect size for treatment A versus control and treatment B versus control, and included these in separate meta‐analyses according to the treatment construct. If treatments A and B represented variations of the same treatment construct, we calculated the weighted mean and SD for treatment A and B before calculating the effect size for the merged group versus control group, following the procedures outlined in Borenstein et al. (2009, ch. 25). Where different studies reported on the same programme but used different samples (e.g., from different regions) we included both estimates, treating them as independent samples, provided that effect sizes were measured relative to separate control or comparison groups.

#### 4.3.3 Selection of studies

We began by importing all search results into EPPI‐Reviewer 4 (Thomas et al., 2010) and removing duplicates. In this review, we took advantage of two innovative text‐mining machine learning (ML) capabilities of EPPI‐Reviewer 4 to reduce the initial screening workload: the priority‐screening function and the inclusion/exclusion classifier (O'Mara‐Eves et al., 2015; Thomas et al., 2011).

Before beginning with the use of these functions the review team double‐screened three batches of records to train consultants on the inclusion and exclusion criteria. Once the training was completed, the priority screening function was utilised. The priority screening function was used at the title and abstract screening stage to prioritise the items most likely to be “included” based on previously included documents. The screening process was conducted by a group of consultants who double‐screened studies through the priority screening function, with reconciliations occurring when there was a disagreement on the inclusion or exclusion decision. For unanticipated reasons, the priority screening function did not produce the expected results and after screening 20% of all records, we utilised the classifier function (a fuller description of this process can be found in Appendix A.2).

Using the studies which had already been screened and coded as included/excluded, we were able to use the classifier function to order the remaining records into probabilities of inclusion. As originally planned, we double‐screened all records above 20% probability of inclusion and we screened a random set of 10% of records below 20% probability of inclusion. Although every record below 20% probability of inclusion within the random sample was excluded, we chose not to automatically exclude the rest of this group. As a precautionary measure, we decided to screen every record below 20%, which still resulted in all records being excluded.

Where a study's title and abstract did not include sufficient information to determine relevance, we included the study for review at full text. We double‐screened all studies flagged for full‐text review using two independent reviewers, resolving disagreements by discussion and the input of an additional reviewer if necessary.

#### 4.3.4 Data extraction and management

We extracted the following descriptive, methodological, qualitative and quantitative data from each included study using standardised data extraction forms, which are provided in Appendix A.5:

- Descriptive data including authors, publication date and status, as well as other information to characterise the study including country, type of intervention and outcome, population and context.
- Methodological information on study design, analysis method and type of comparison.
- Quantitative data for outcome measures, including outcome descriptive information, sample size in each intervention group, outcomes means and SDs, and test statistics (e.g., *t* test, *F* test, *p* values, 95% confidence intervals).
- Information on intervention design, including how the intervention incorporates participation, inclusion, transparency and accountability characteristics, participant adherence, contextual factors, and programme mechanisms.

We extracted descriptive, methodological, qualitative, and quantitative data using Excel. Descriptive and qualitative data was double‐coded by two consultants and checked by a review team member. Two independent core reviewers double‐coded quantitative data for outcomes analysis, and disagreement were resolved through discussion with a third.

Once all effect sizes were calculated and converted to a standardised mean difference (SMD; as described in detail below), we examined the data for outliers. We defined outliers as any effect sizes ±3.29 SDs from the mean, following the guidance of Tabachnick and Fidell (2001). Outliers were windsorised if necessary, as described by these authors, as is suggested for outliers in meta‐analysis (Lipsey & Wilson, 2001). Sensitivity to outliers was examined as discussed in the section on sensitivity analysis below.

#### 4.3.5 Assessment of risk of bias in included studies

We have assessed the risk of bias in the included studies by drawing on the signalling questions in the 3ie risk of bias tool, which covers both internal validity and statistical conclusion validity of experimental and quasi‐experimental impact evaluation designs (Hombrados & Waddington, 2012). It includes the bias domains and extensions to Cochrane's ROBINS‐I tool and RoB2.0 (Higgins et al., 2016; Sterne et al., 2016). The risk of bias assessment helps us to determine the extent to which the findings in each study are reliable. Two reviewers undertook the risk of bias assessment independently. If there were any disagreements, we resolved them by discussion and the involvement of a third reviewer, as necessary. The risk of bias tool can be found in Appendix A.6. We conducted the risk of bias at the paper level, noting any potential differences in methods and risk of bias by different outcomes.

We assessed risk of bias based on the following criteria, coding each paper as “Yes”, “Probably Yes”, “Probably No”, “No” and “Unclear” according to how they address each domain:

- Factors relating to baseline confounding and biases arising from differential selection into and out of the study (e.g., assignment mechanism).
- Factors relating to bias due to missing outcome data (e.g., assessment of attrition).
- Factors relating to biases due to deviations from intended interventions (e.g., performance bias and survey effects) and motivation bias (Hawthorne effects).
- Factors relating to biases in outcomes measurement (e.g., social desirability or courtesy bias, recall bias).
- Factors relating to biases in reporting of analysis.

We have reported the results of the appraisal for each of the assessed criteria for each study. In addition, we used the results of the risk of bias assessments to produce an overall rating for each study as either “High risk of bias”, “Some concerns” or “Low risk of bias”, drawing on the decision rules in RoB2.0 (Higgins et al., 2016), rating studies as follows:

- “High risk of bias”: if any of the bias domains were assessed as “No” or “Probably No”.
- “Some concerns”: if one or several domains were assessed as “Unclear” and none were “No” or “Probably No”.
- “Low risk of bias”: if all of the bias domains were assessed as “Yes” or “Probably Yes”.

In addition, we explored the presence of systematic differences in outcome effects between primary studies with different risk of bias. If meta‐analysis was feasible, we conducted sensitivity analysis to assess the robustness of the results to the risk of bias in included studies.

#### 4.3.6 Measures of treatment effect

An effect size expresses the magnitude (or strength) and direction of the relationship of interest (Borenstein et al., 2009; Valentine et al., 2015). We extracted data from each individual study to calculate standardised effect sizes for cross‐study comparison wherever possible. For continuous outcomes comparing group means in a treatment and control group, we calculated the SMDs, or Cohen's *d*, its variance and SE using formulae provided in Borenstein et al. (2009). A SMD is a difference in means between the treatment and control groups divided by the pooled SD of the outcome measure. Cohen's *d* can be biased in cases where sample sizes are small. Therefore, in all cases we will simply adjust *d* using Hedges' method, adjusting Cohen's *d* to Hedges' *g* using the following formula (Ellis, 2010):

$$g≅d\left(1-\frac{3}{4(n_{1}+n_{2})-9}\right).$$

We chose the appropriate formulae for effect size calculations in reference to, and dependent upon, the data provided in included studies. For example, for studies reporting means (*X*) and pooled SD for treatment (*T*) and control or comparison (*C*) at follow up only:

$$d=\frac{x_{\mathrm{Tp}+1}-x_{\mathrm{Cp}+1}}{SD}.$$

If the study did not report the pooled SD, we calculated it using the following formula:

$${SD}_{p+1}=\sqrt{\frac{(n_{\mathrm{Tp}+1}-1){SD}_{\mathrm{Tp}+1}^{2}+(n_{\mathrm{Cp}+1}-1){SD}_{\mathrm{Cp}+1}^{2}}{n_{\mathrm{Tp}+1}+n_{\mathrm{Cp}+1}-2}},$$

where the intervention was expected to change the SD of the outcome variable, we used the SD of the control group only.

For studies reporting means ($\underline{X}$) and SDs for treatment and control or comparison groups at baseline (*p*) and follow up (*p* + 1):

$$d=\frac{{∆\underline{X}}_{p+1}-{∆\underline{X}}_{p}}{{SD}_{p+1}}.$$

For studies reporting mean differences ($∆\underline{X}$) between treatment and control and SD at follow up (*p* + 1):

$$d=\frac{{∆\underline{X}}_{p+1}}{{SD}_{p+1}}=\frac{{\underline{X}}_{\mathrm{Tp}+1}-{\underline{X}}_{\mathrm{Cp}+1}}{{SD}_{p+1}}.$$

For studies reporting mean differences between treatment and control, SE and sample size (*n*):

$$d=\frac{{∆\underline{X}}_{p+1}}{SE\sqrt{n}}.$$

As primary studies have become increasingly complex, it has become commonplace for authors to extract partial effect sizes (e.g., a regression coefficient adjusted for covariates) in the context of meta‐analysis. For studies reporting regression results, we followed the approach suggested by Keef and Roberts (2004) using the regression coefficient and the pooled SD of the outcome. Where the pooled SD of the outcome was unavailable, we used regression coefficients and SEs or *t* statistics to do the following, where sample size information was available in each group:

$$d=t\sqrt{\frac{1}{n_{T}}+\frac{1}{n_{C}}},$$

where *n* denotes the sample size of treatment group and control. We used the following where only the total sample size information (*N*) was available, as suggested in Polanin et al. (2016):

$$d=\frac{2t}{\sqrt{N}}\;{\mathrm{Var}}_{d}=\frac{4}{N}+\frac{d^{2}}{4N}.$$

We calculated the *t* statistic (*t*) by dividing the coefficient by the SE. If the authors only reported confidence intervals and no SE, we calculated the SE from the confidence intervals. If the study did not report the SE, but reported *t*, we extracted and used this as reported by the authors. In cases in which significance levels were reported rather than *t* or SE (b), then *t* was imputed as follows:

$$\begin{array}{cccc} & \text{Prob}\;> & 0.1: & t=0.5,\\ 0.1 & \geq \;\text{Prob}\;> & 0.05: & t=1.8,\\ 0.05 & \geq \;\text{Prob}\;> & 0.01: & t=2.4,\\ 0.01 & \geq \;\text{Prob}: & & t=2.8, \end{array}$$

where outcomes were reported in proportions of individuals, we calculated the Cox‐transformed log odds ratio effect size (Sánchez‐Meca et al., 2003):

$$d=\frac{\ln(\mathrm{OR})}{1.65},$$

where OR is the odds ratio calculated from the two‐by‐two frequency table.

Where outcomes were reported based on proportions of events or days, we used the standardised proportion difference effect size:

$$d=\frac{p_{T}-p_{C}}{SD(p)},$$

where *p*_*t*_ is the proportion in the treatment group and *p*_*c*_ the proportion in the comparison group, and the denominator is given by:

$$SD(p)=\sqrt{p(1-p)},$$

where p is the weighted average of *p*_*c*_ and *p*_t_:

$$p=\frac{n_{T}p_{T}+n_{C}p_{C}}{n_{T}+n_{C}}.$$

An independent reviewer evaluated a random selection of 10% of effect sizes to ensure that the correct formulae were employed in effect size calculations. In all cases after synthesis, we converted pooled effect sizes to commonly used metrics such as percentage changes and mean differences in outcome metrics typically used (e.g., weight in kg) whenever feasible.

#### 4.3.7 Unit of analysis issues

Unit of analysis errors can arise when the unit of allocation of a treatment is different to the unit of analysis of effect size estimate, and this is not accounted for in the analysis (e.g., by clustering SEs at the level of allocation). We assessed studies for unit of analysis errors (The Campbell Collaboration, 2019), and where they exist, we corrected for them by adjusting the SEs according to the following formula (Hedges, 2009; Higgins et al., 2020; Waddington et al., 2012):

$$SE{\left(d\right)}^{\prime }=SE(d)*\sqrt{1+(m-1)c},$$

where *m* is the average number of observations per cluster and *c* is the intra‐cluster correlation coefficient. Where included studies used robust Huber‐White SEs to correct for clustering, we calculated the SE of *d* by dividing *d* by the *t* statistic on the coefficient of interest.

#### 4.3.8 Dealing with missing data

In cases of relevant missing or incomplete data in studies identified for inclusion, we have made every effort to contact study authors to obtain the required information. If we were unable to obtain the necessary data, we reported the characteristics of the study but state that it could not be included in the meta‐analysis or reporting of effect sizes due to missing data.

#### 4.3.9 Assessment of heterogeneity

We have assessed heterogeneity by calculating the *Q* statistic, *I* ^2^ and *τ* ^2^ to provide an estimate of the amount of variability in the distribution of the true effect sizes (Borenstein et al., 2009). We complemented this with an assessment of heterogeneity of effect sizes graphically using forest plots. Due to the data extracted from included studies, we were able to explore heterogeneity using moderator analysis in bivariate meta‐regression specifications.

#### 4.3.10 Assessment of reporting biases

To reduce the possibility of publication bias, we have searched for and included unpublished studies in the review. We also tested for the presence of publication bias through the use of contour‐enhanced funnel graphs (Peters et al., 2008) and statistical tests (Egger et al., 1997). Drawing on methodologies used in previous work, such as the COMPare Trials Project (Goldacre et al., 2016), and trying to capitalise on recent shifts towards preregistration of studies and their associated preanalysis plans, we also examined whether studies that were pre‐registered (e.g., on platforms such as ClinicalTrials.gov, the Open Science Foundation, the American Economic Association's trial registry, or the RIDIE) report on all of the outcomes that were proposed in their preanalysis plans.

#### 4.3.11 Data synthesis

We conducted meta‐analyses of studies that we assessed to be sufficiently similar. The inclusion criteria for the review were broad and we anticipated including studies reporting on a diverse set of interventions, sectors and outcomes. However, we only combined studies using meta‐analysis when we identified two or more effect sizes using a similar outcome construct and where the comparison group was judged to be similar across the two, similar to the approach taken by Wilson et al. (2011). We combined studies in the same analysis when they evaluated the same intervention type, or the same outcome type. Moderator analyses were conducted to take into account multiple interventions as moderator variables, allowing us to also examine the impact of different intervention types by outcome. Where there were too few studies, or included studies were considered too heterogeneous in terms of interventions or outcomes, we present a discussion of individual effect sizes along the causal chain. As heterogeneity exists in theory due to the variety of interventions and contexts included, we used inverse‐variance weighted, random effects meta‐analytic models (Higgins et al., 2020).

We have used the *metafor* package (Viechtbauer, 2010) and/or the *robumeta* package (Fisher & Tipton, 2015) in R software to conduct the meta‐analyses (R Core Team, 2020).

We conducted separate analyses for the major outcome categories: productivity, income, nutrition and women's empowerment. Based on an analysis of the interventions that we found, we attempted to further elaborate on the pathway of change that was outlined above to the extent possible. We also used sub‐group analysis to explore heterogeneity by different treatment subgroups (described in more detail in the section on subgroup analysis and investigation of heterogeneity). We also collected qualitative information from studies about the interventions. This information was coded quantitatively to be used in the moderator analysis. It was also used to classify intervention mechanisms in synthesis or in the further development of intervention causal chains.

#### 4.3.12 Subgroup analysis and investigation of heterogeneity

Whenever feasible, we conducted moderator analyses to investigate sources of heterogeneity. Following the PROGRESS‐PLUS approach (Oliver et al., 2017), we assessed 18 moderators falling into three broad categories of extrinsic, methodological and substantive characteristics to address inequity aspects within the aquaculture context, including:

- Extrinsic characteristics: publication type, and year of publication.
- Methodological characteristics: study design, comparison group, risk of bias, evaluation period, and the need to make assumptions when extracting effect sizes due to missing data in the reports.
- Substantive characteristics: context (continent and country of intervention, exposure to climate shocks index, World Bank country income group), and key intervention features (scale, number of beneficiaries, aquaculture and nonaquaculture components, community or individuals focus, value chain stage, and months of exposure to intervention).

Whenever possible, we have used random effects meta‐regression to investigate the association between moderator variables and heterogeneity of treatment effects (Borenstein et al., 2009) and subgroup analyses to investigate heterogeneity by treatment sub‐groups (e.g., men and women, poor, and nonpoor, and so on). In cases when there is not sufficient data and these strategies were not possible, we have discussed and explored the factors which may be driving heterogeneity of results narratively by conducting cross‐case comparisons (Miles & Huberman, 1994).

#### 4.3.13 Sensitivity analysis

We conducted sensitivity analysis to assess whether the results of the meta‐analysis are sensitive to the removal of any single study. We have done this by removing studies from the meta‐analysis one‐by one and assessing changes in results. We also assessed sensitivity of results to inclusion of high risk of bias studies by removing these studies from the meta‐analysis and comparing results to the main meta‐analysis results. Finally, when relevant, we have assessed sensitivity to outliers by comparing results with and without outliers included, as well as results when outliers are windsorised.

#### 4.3.14 Treatment of qualitative research

We used qualitative research to supplement the findings of the interventions covered by included studies. While we did not seek out all qualitative studies relating to aquaculture activities in low‐ and middle‐income countries, we searched for qualitative studies to provide additional information about the context and implementation of interventions included in the quantitative synthesis. Specifically, we used this to address research question 4, employing the aforementioned “effectiveness+” framework (Snilstveit, 2012). This included feasibility studies, stakeholder analyses, formative evaluations, process evaluations, project reports, among other documents. These sources provided inputs to our analysis of the facilitators and inhibitors of aquaculture interventions.

#### 4.3.15 Treatment of cost data

To address review question 5, we used cost data reported in the set of included studies or in additional studies identified through the second search of references. Following Shemilt et al. (2008), relevant studies included full economic evaluations (e.g., cost‐benefit, cost‐effectiveness or cost‐utility analyses), partial economic evaluations (e.g., cost analyses, cost‐comparison studies, cost‐outcome descriptions), or any other documentation reporting cost data of included interventions.

Full and partial economic evaluation studies were appraised in terms of the cost and/or effectiveness components reported and used in the analyses. In turn, general descriptions of cost information of included interventions were synthesised narratively. If there was relevant data on the costs and effects of an intervention reported separately, we extracted data on the resources, unit and/or total costs with the aim to examine both components. In these cases, we focused on comparable outcomes if possible. We also noted when included studies found statistically nonsignificant effects, however, we did not include nonsignificant impacts in the cost‐effectiveness analysis (Dhaliwal et al., 2013). If this impact is precisely measured, then there is little relevance in examining noneffective interventions; whereas if the impact is measured with less precision, there will be uncertainty around the real effectiveness of the intervention, which would have affected the analysis around its cost.

## 5 RESULTS

### 5.1 Description of studies

#### 5.1.1 Results of the search

**Figure 2:**
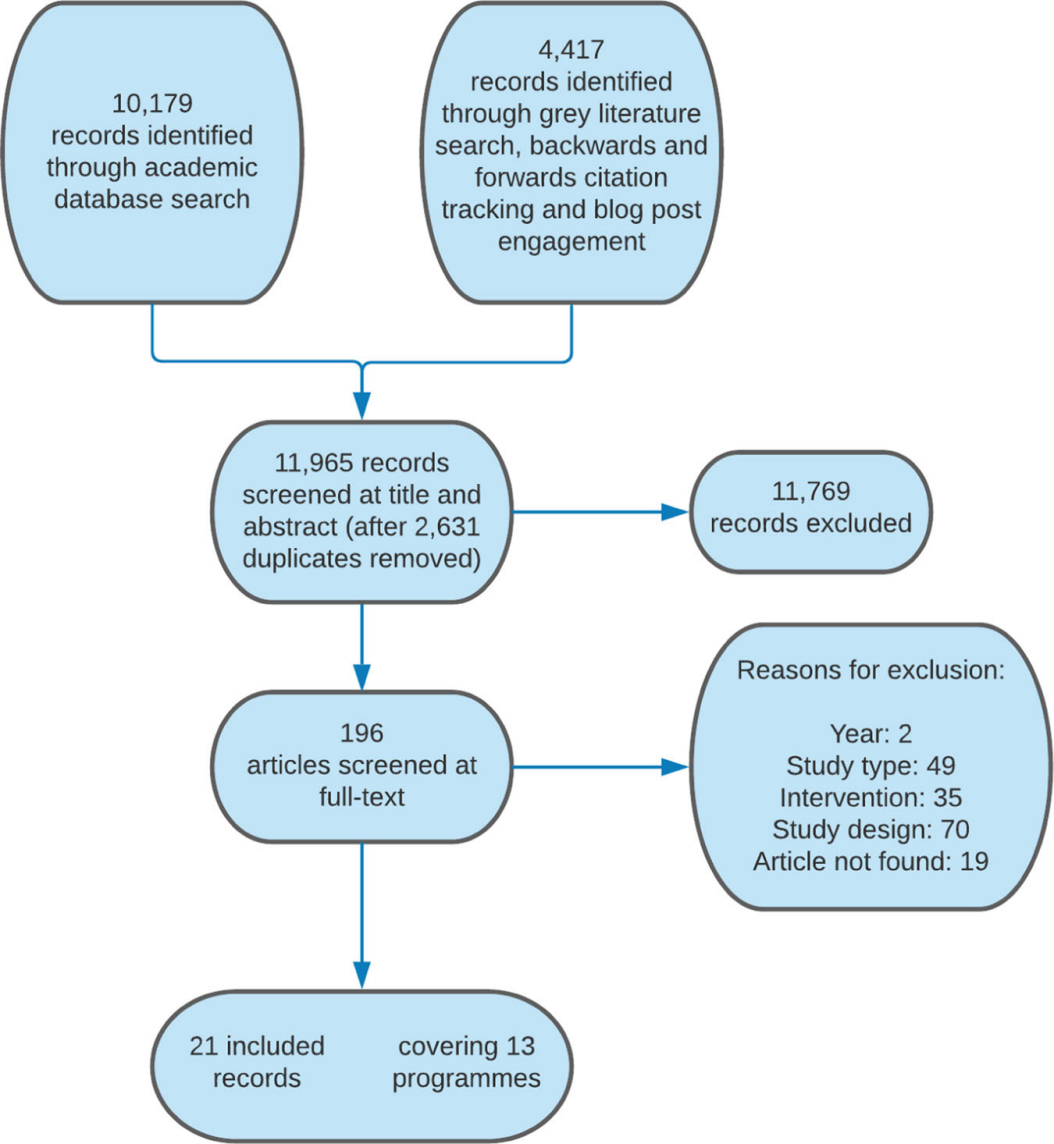
PRISMA flow diagram for the review

The PRISMA flow diagram in Figure 2 shows the results of our main search to address review questions 1–3. Appendices A.1–A.4 provide more information on the search and screening process, as well as fully explained examples of our inclusion and exclusion criteria.

The search of five academic databases led to the identification of 10,179 records. In turn, searching 21 grey literature sources and forwards and backwards citation tracking of relevant reviews and included records led to the identification of 4414 records. Three further studies were obtained after authors contacted the review team in response to an outreach activity. Of the 14,596 records identified, 2631 were marked as duplicates, leading to 11,965 records being screened at the title and abstract level. Screening at title and abstract led to the exclusion of 11,769 records, leaving 196 records for full‐text screening. As 19 records could not be retrieved, 177 papers were screened at full‐text.

The main reason for exclusion at the full‐text stage was lack of an appropriate study design (*n* = 70), meaning that the was no comparison group, the study used an inappropriate comparison group, or that the analysis did not utilise one of the methods defined for this review, as described previously. The second main reason for exclusion was incorrect study type (*n* = 49), where most often papers were not primary studies or were not attempting to evaluate the impact of an intervention. An irrelevant intervention (*n* = 35), when a paper was not focused on aquaculture, was the next most common reason for exclusion, and finally publication date pre‐1980 (*n* = 2) was the least‐used exclusion code at this stage. This process resulted in the inclusion of 21 impact evaluations covering 13 unique programmes, which were deemed to meet all eligibility criteria.

To address review questions 4 and 5, we carried out a second search for documents related to the included programmes. The details of the additional search are included in Appendix A.7. Once we had identified all included papers, the programmes were searched using Google and institutional websites whenever the programmes were clearly implemented or funded by an organisation. From this search we were able to identify 21 additional documents related to barriers and facilitators of included aquaculture programmes. Moreover, we were able to identify some form of cost data for nine of the included programmes: Adivasi Fisheries Project (AFP), Community Based Fish Culture in Seasonal Floodplains and Irrigation Systems (CBFC), Development of Sustainable Aquaculture Project (DSAP), Economic Stimulus Programme (ESP), Third National Fadama Development Project (Fadama III), Greater Noakhali Aquaculture Extension Project (GNAEP), Mymensingh Aquaculture Extension Project (MAEP), Sustainable Agriculture, Food Security and Linkages Project (SAFAL), and Sustainable Market Access through Responsible Trade of Fish (SMART‐Fish). Despite our efforts, we could not locate cost data for NGO Banchte Shekha (BS), Second National Fadama Development Project (Fadama II), Fish on Farms (FoF), and WorldFish Integrated Aquaculture‐Agriculture Dissemination (IAA).

All searches, including academic and grey literature sources, forward and backward citation tracking, and the second search for additional documentation, were conducted in the UK (Cooper et al., 2021).

#### 5.1.2 Included studies

This section provides an overview of the characteristics of the included studies. Table 1 summarises these papers, presenting a description of the programmes they cover and the design, comparison group, and outcomes identified for each included study. For the rest of the report, we will refer to these programmes using the acronyms shown in parenthesis in this table. Additionally, Appendix A.4 provides more details on the exclusion criteria used and examples of borderline inclusion decisions.

**Table 1:** Main characteristics of included studies

Programme and country	Intervention description	Papers	Study design	Comparison group	Outcomes measured
Adivasi Fisheries Project (AFP) Bangladesh	Implemented by WorldFish with different national partners, AFP aimed at increasing fish pond production, household nutrition, income and alternative employment opportunities of the Adivasi (Indigenous) people. Training on the promotion of small‐scale aquaculture and aquatic enterprise development was used to deliver these aims	Saiful Islam et al. (2015)	PSM	Business as usual	Income: household income, farm income. Nutrition: days of fish consumption, meals with fish, quantity of fish consumption
		Saiful Islam (2015)	PSM and DiD	Business as usual	Income: household expenditure, household food expenditure
Community Based Fish Culture in Seasonal Floodplains and Irrigation Systems (CBFC) Bangladesh	Jointly implemented by WorldFish, the Bangladesh Agricultural Research Council, and the Department of Fisheries, CBFC consisted of two main components: (1) the construction of enclosures for fish culture in the flood season and (2) the stocking of fingerlings and fish culture during the flood season	Haque and Dey (2017)	PSM and DiD	Business as usual	Income: fish income, nonfish income, total household income. Nutrition: per capita fish consumption
		Haque and Dey (2016)	PSM	Business as usual	Income: food expenditure, nonfood expenditure, total household expenditure
Development of Sustainable Aquaculture Project (DSAP) Bangladesh	Headed by WorldFish with funding from USAID, and implemented by 48 partner NGOs, DSAP was implemented in 42 of 64 Bangladeshi districts deemed suitable for aquaculture work. The project aimed at improving resource‐use efficiency to increase farm‐level productivity through the dissemination of low‐cost aquaculture technologies and continuous training	Khondker and Pemsl (2011)	ANOVA	Business as usual	Income: net income. Productivity: gross income, total factor productivity
Mymensingh Aquaculture Extension Project (MAEP) Bangladesh	Funded by the Danish International Development Agency, MAEP targeted both poor and better‐off households and provided training on polyculture fish technology, which had been developed by WorldFish	Rand and Tarp (2009)	PSM and DiD	Business as usual Pipeline	Productivity: fish production, pond productivity. Income: household expenditure
		DANIDA (2008)	PSM and DiD	Pipeline	Productivity: fish production. Income: household expenditure, household assets. Women's empowerment: index of freedom of movement
		Quisumbing and Kumar (2011)	PSM	Business as usual	Women's empowerment: wife and husband's assets and ownership change
		Kumar and Quisumbing (2010)	This paper is a working paper of Quisumbing and Kumar (2011); as such, all outcomes from this paper were extracted from the latest publication		
		Hallman et al. (2003)	Instrumental variable	Pipeline	Income: household income, household expenditure, crop profit, farm and nonfarm income. Nutrition: anthropometric indicators. Women's empowerment: freedom of movement and domestic violence
Greater Noakhali Aquaculture Extension Project (GNAEP) Bangladesh	Funded by DANIDA, GNAEP was developed based on the model of MAEP. An intensive training programme was used to improve techniques in carp polyculture. From 2002, the programme had a more explicit pro‐poor strategy, which was centred on the promotion of prawn polyculture	DANIDA (2008)	PSM and DiD	Business as usual	Productivity: household fish production. Income: household income. Women's empowerment: index of freedom of movement
NGO Banchte Shekha (BS) Bangladesh	BS targeted poor women and with their aquaculture intervention arranged for the lease of ponds to women's groups. As well as providing the credit to lease the ponds, the NGO arranged training on polyculture fish production	Quisumbing and Kumar (2011)	PSM	Business as usual	Women's empowerment: wife and husband's assets and ownership change
		Kumar and Quisumbing (2010)	This paper is a working paper of Quisumbing and Kumar (2011); as such, all outcomes from this paper were extracted from the latest publication		
		Hallman et al. (2003)	Instrumental variable	Pipeline	Income: household income, household expenditure, crop profit, farm and nonfarm income. Nutrition: anthropometric indicators. Women's empowerment: freedom of movement and domestic violence
Sustainable Agriculture, Food Security and Linkages Project (SAFAL) Bangladesh	SAFAL intervened in key stages of the aquaculture, horticulture and dairy value chains. The project facilitated the formation of producer groups, provided training on new farming practices, encouraged entrepreneurs to provide services to local farmers, and represented the producer groups in coordination and negotiation activities with services companies and potential buyers	Kuijpers (2020)	PSM and DiD	Business as usual	Productivity: production value. Income: farm income, farm revenue, farm profit, wage income. Nutrition: length of hungry season
		Kuijpers (2019)	PSM and DiD	Business as usual	Income: market participation
Fadama II Nigeria	Fadama II was implemented by the Nigerian government as a follow up to Fadama I. The main objective was to sustainably increase the incomes of Fadama users through expansion of farm and nonfarm activities. The main use of resources was for asset acquisition, with an emphasis to reach marginalised users and nonlandowners	Akinlade (2012)	PSM and DiD	Business as usual	Income: poverty incidence, poverty gap index, severity of poverty index
Fadama III Nigeria	Fadama III was a follow up to Fadama II with similar aims. The difference between the two projects being that with Fadama III there was a greater emphasis on growth sustainability. A Fadama User's Equity Fund was also formed to create saving bank accounts for Fadama users	Alawode and Oluwatayo (2019)	PSM	Business as usual	Productivity: production level
Fish on Farms (FoF) Cambodia	FoF was a programme targeting women and children in rural Cambodia, aiming to increase fish and vegetable consumption and in turn, nutrition outcomes of mothers and children. The randomised controlled trial had three arms: an agricultural arm supplying training and inputs to create a home garden for vegetable production, an aquaculture arm providing the same inputs as the vegetable arm plus additional inputs and training to create a home pond for fish production, and a control group	Michaux et al. (2019)	RCT	Business as usual	Nutrition: blood indicators, anthropometric measures
		Verbowski et al. (2018)	RCT	Business as usual	Nutrition: micronutrients intake indicators
		Talukder and Green (2014)	RCT	Business as usual	Income: earnings from fish sales. Nutrition: household food security score
SMART‐Fish Indonesia	SMART‐Fish tackled two sectors of aquaculture: pangasius and seaweed farming. The project had five stages: (1) identify challenges facing farmers; (2) select partners to carry out trials; (3) select farmers to test the training, technology, and dissemination strategy; (4) disseminate the trial results to other farmers; (5) replicate the trial in others areas	Cahyadi and Bahramalian (2019)	PSM	Business as usual	Productivity: productivity, weight, mortality. Income: price received, income
WorldFish Integrated Aquaculture‐Agriculture Dissemination (IAA) Malawi	IAA utilised the farm's crop residues to help increase fish production. WorldFish used a participatory approach to its dissemination activities, which involved the interaction of farmers and researchers	Dey et al. (2010)	PSM	Business as usual	Income: farm income
Economic Stimulus Programme (ESP) Kenya	In 2009 the Kenyan government created the Economic Stimulus Package. As part of this the aquaculture sector received $15 million, for its development. This money was used to construct 200 fish ponds, as well as provide the fingerlings and inputs needed to fish from these ponds	Amankwah et al. (2018)	PSM	Business as usual	Income: fish income, poverty incidence
		Amankwah (2016)	This paper is a dissertation, whereas Amankwah et al. (2018) is the journal article version; hence we extracted outcomes from the latest publication		

*Note*: ANOVA, analysis of variance; DiD, difference‐in‐difference; PSM, propensity score matching; RCT, randomised controlled trial.

Although we identified 21 records for inclusion, only 19 records were used in our synthesis. In two cases, records are linked to another included study, meaning that they presented the same analysis of the same intervention but in another publication version. Quisumbing and Kumar (2011) is the published journal article version of Kumar and Quisumbing (2010), which is a working paper. Similarly, Amankwah (2016) is a dissertation, whereas Amankwah et al. (2018) is the published journal article version of the same analysis. For these two cases, we extracted information from the most recently published paper. Similarly, Kuijpers (2019) is the working paper version of Kuijpers (2020), which is the published article. The reason both records have been included in our synthesis is that there was one additional outcome reported in the 2019 paper.

**Figure 3:**
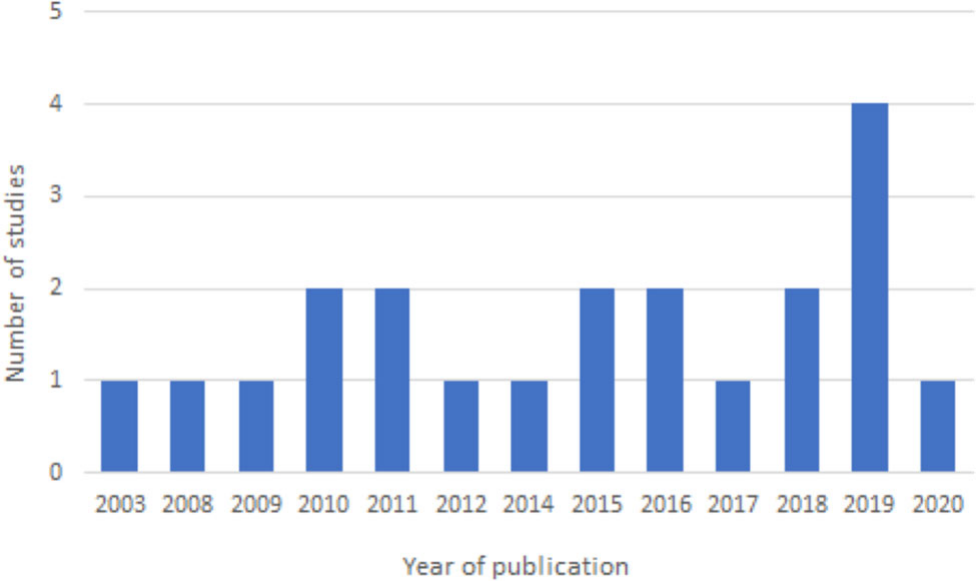
Year of publication of included studies

Of the 21 included papers, 13 are from journal articles, three are working or discussion papers, three are institutional reports, one is a dissertation, and one is a conference paper. Figure 3 shows the year of publication for the included papers, indicating that, despite searching for papers published from 1980 onwards, we were only able to identify relevant studies from 2003, half of which were published in the last 5 years.

**Figure 4:**
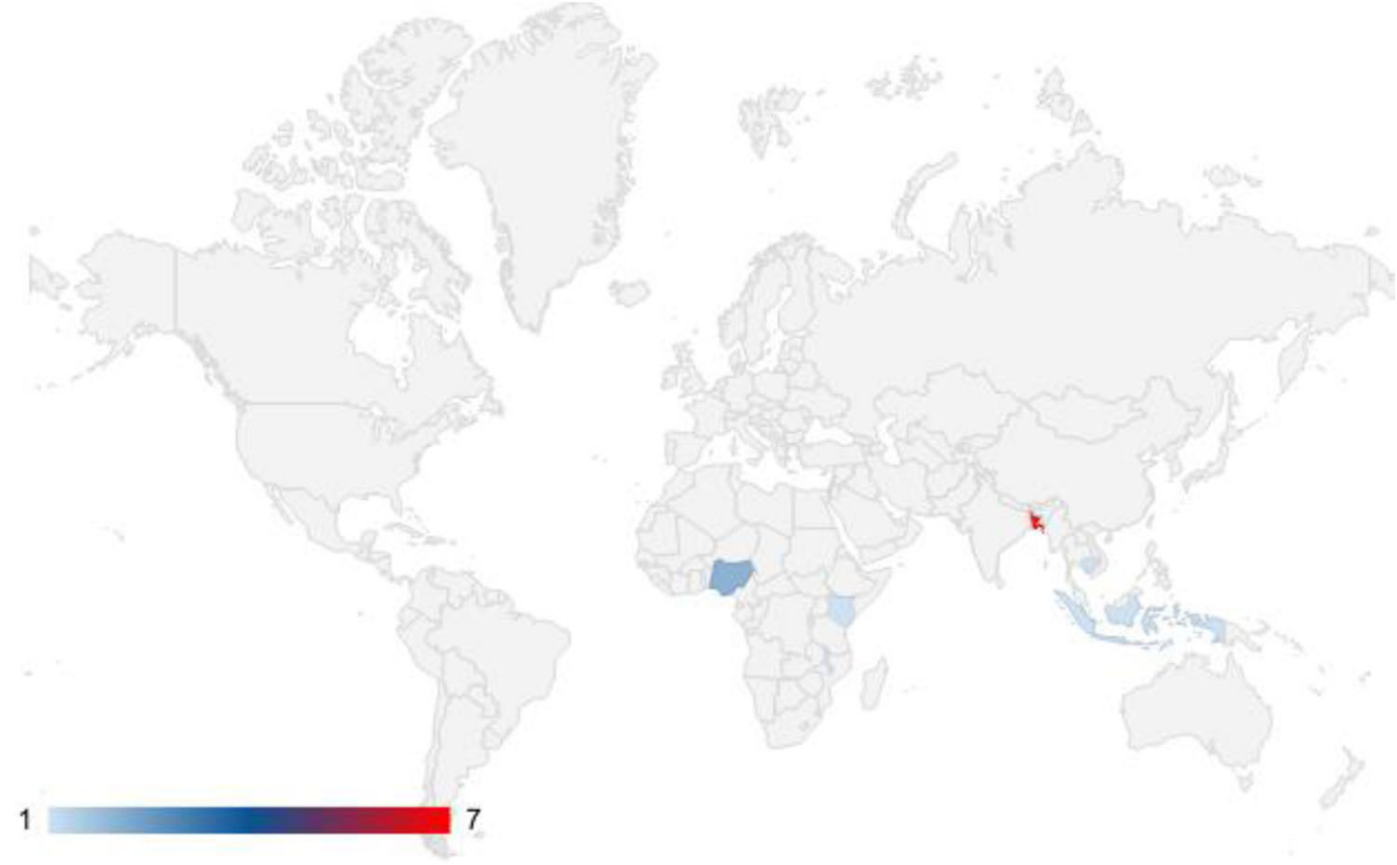
Geographical location of included interventions

The map in Figure 4 shows the geographical distribution of the 13 programmes included in our analysis. Half of these programmes (*n* = 7), took place in Bangladesh (AFP, BS, CBFC, DSAP, GNAEP, MAEP and SAFAL). This corresponds to 12 of the included records. Two other programmes were implemented in Asia, one in Cambodia (FoF) which is reported in three included papers, and one in Indonesia (SMART‐Fish), covered by one included paper. A further four programmes were implemented in Africa. Two of them were based in Nigeria (Fadama II and Fadama III), which are reported in one paper each, one programme was implemented in Kenya (ESP), covered by two papers, and one programme took place in Malawi (IAA), which corresponds to one included study.

Following the Country and Lending Group Classification (World Bank, n.d.), we classified each country's income group based on the 1st year of implementation for each programme. Ten interventions took place in low‐income countries, while three were based in lower‐middle income countries: Nigeria, Bangladesh, and Indonesia. Bangladesh and Nigeria are classified in both income groups due to the different years the programmes were initially implemented and the fact that these are countries in transition: Bangladesh transitioned from a low‐income to a lower‐middle income country in 2014, whereas Nigeria had the same change in 2008. We did not identify relevant studies implemented in middle‐income countries.

We also classified a country's exposure to climate shocks, such as floods, sea‐level rise, storms, droughts and earthquakes, as defined by the World Risk Index (BEH, n.d.) for the 1st year of a programme's implementation. If the programme began before these classifications were available, we used the earliest possible country classification. The exposure index assesses the risk of countries to face disasters as the result of extreme natural events. It is measured as a weighted score out of 100, where each quintile is categorised from “very low” to “very high”. The majority of the programmes were implemented in a country with a very high exposure to climate shocks, which includes all interventions based in Bangladesh (*n* = 7), Cambodia (*n* = 1) and Indonesia (*n* = 1). Malawi (*n* = 1) was the only country with a high exposure to climate shocks, whereas the programmes in Nigeria (*n* = 2) and Kenya (*n* = 1) were classified with a medium exposure to climate shocks. Therefore, the countries covered by our included studies have a considerable exposure to natural hazards as they are categorised in the highest quintiles of the index.

#### 5.1.3 Participants

In terms of the population that each intervention was targeting, in many cases there was a lack of information in the included studies (see Table 2). What Table 2 records is not the characteristics of the sample participants who ultimately are part of the analyses in each report; instead, we aimed to capture the profile of the population originally targeted by the programmes. For five programmes, there was an explicit statement that they took place in rural locations, whereas for the others (*n* = 8), the reports did not state their specific location. Two programmes explicitly targeted women (BS and FoF), seven programmes targeted both men and women (AFP, DSAP, Fadama II, Fadama III, GNAEP, MAEP and SAFAL), while four did not make this information clear (CBFC, ESP, IAA and SMART‐Fish). The majority of programmes (*n* = 12), provided no information on the age of the targeted beneficiaries, whereas we know Fish on Farms targeted 18–45‐year‐old mothers who had children aged 6–59 months. Six interventions identified targeted beneficiaries as being from a low‐socioeconomic background (AFP, BS, DSAP, Fadama II, FoF and GNAEP), whereas the others did not provide this information. Finally, we sought to identify the educational background of the programme's targeted participants but no papers provided this information.

**Table 2:** Main characteristics of included programmes

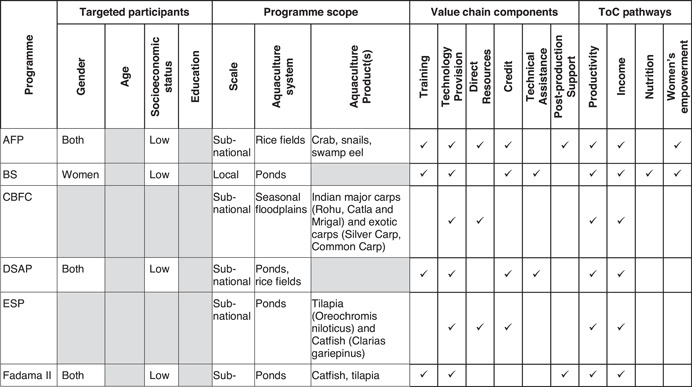
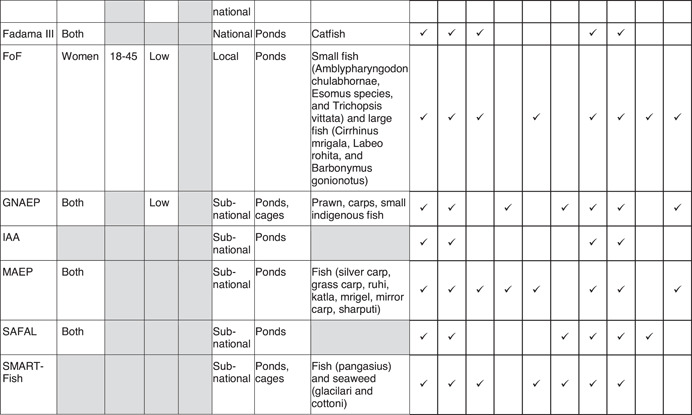

*Note*: Cells shaded in grey indicate that such information was not provided in the papers.

One area of interest to this review was whether programmes targeted participants who did or did not own the land on which the production took place on. Though five papers did not state this explicitly, three programmes targeted owners (DSAP, IAA and MAEP), three programmes targeted nonowners (AFP, BS and CBFC), and two targeted both owners and nonowners (Fadama III and GNAEP). Some of these programmes had a focus on nonlandowners. BS focused on providing credit to women's groups for them to rent the land to carry out aquaculture activities; AFP had a focus on bringing landless Adivasi households into the programme; and CBFC focused on community based seasonal floodplains not owned by any one individual.

Another area of interest in terms of target beneficiaries was how the interventions focused on individual vis‐à‐vis communities. Seven programmes targeted their activities at individuals (DSAP, ESP, Fadama II, Fadama III, FoF, GNAEP and IAA), while the other six programmes focused their activities in groups or communities (AFP, BS, CBFC, MAEP, SAFAL and SMART‐Fish). Examples of programmes targeting individuals are ESP, which provided inputs to farmers, and FoF, which provided inputs to women to create home‐ponds in their own gardens. In turn, programmes relying on trained farmers to act as demonstration points, such as AFP and SMART‐Fish, clearly focused on communities.

#### 5.1.4 Comparisons, outcomes and evaluation designs

Within the 19 included papers used in the synthesis, only two types of comparison groups were identified. Sixteen papers compared the intervention group to a control which received no intervention, labelled as business as usual; one study (Hallman et al., 2003) compared the intervention to a control group which would later receive the same intervention, labelled as pipeline; and two papers (DANIDA, 2008; Rand & Tarp, 2009) included both comparison groups. The three papers including a pipeline comparison group used the same data set, which provided short‐term data for an intervention group and a group which would later receive the intervention. These papers evaluated the BS, GNAEP and MAEP programmes.

As previously stated, we sought to identify all outcomes related to productivity, income, nutrition, and female empowerment. Six programmes evaluated outcomes related to productivity (DSAP, Fadama III, GNAEP, MAEP, SAFAL and SMART‐Fish). The main types of outcomes reported for productivity were production value and production volume. SMART‐Fish also assessed the quality of the production in terms of the colour of the aquaculture products. Only one programme did not provide any outcomes related to income, this was Fadama III. At least one paper for each of the other programmes reported an outcome related to income. Examples of the outcomes reported are: household income, farm profit, household total expenditure, household food expenditure and poverty incidence. Six programmes reported outcomes related to nutrition (AFP, BS, CBFC, FoF, MAEP and SAFAL) with there being variation in the specific types of outcomes reported. Anthropometric measurements were reported for three programmes (BS, FoF and MAEP), fish consumption was reported for two programmes (AFP and CBFC), and food security measures were reported for two interventions (FoF and SAFAL). In addition, FoF, whose main focus was health and nutrition, reported a number of markers pertaining to micronutrients intake and blood concentration measures. Disappointingly, only three programmes provided outcomes related to women's empowerment (BS, GNAEP and MAEP), and these varied substantially. While two reports present these outcomes using a number of individual indicators covering different aspects of female independence and ownership, the third study measured women's empowerment using an index.

The majority of studies included in our synthesis used propensity score matching as the evaluation design, either on its own (*n* = 7) or paired with difference‐in‐differences analysis (*n* = 7). Three papers reported on the FoF project, which was a randomised control trial. One paper used ANOVA (Khondker & Pemsl, 2011) and one paper used village characteristics (i.e., distance from the village to the main health centre and the office of the programme organisation) as instrumental variables (Hallman et al., 2003).

#### 5.1.5 Interventions

An important aspect of the included interventions is the scale at which they operated. Two programmes were implemented locally (BS and FoF), meaning that the programmes only took place in one region of Bangladesh and Cambodia, respectively. Ten programmes operated at the subnational level (AFP, CBFC, DSAP, ESP, Fadama II, GNAEP, IAA, MAEP, SAFAL and SMART‐Fish), that is, they operated across more than one region in their respective countries. Only one programme (Fadama III) operated at a national level.

Similar to scale, the length of these interventions gives an idea of the longevity of the programmes. Of the programmes whose information could be identified (all except BS), the mean programme length was almost 7 years (*M* = 82 months; *SD* = 61 months). The majority of programmes lasted between 30 and 99 months in length. SAFAL lasted for 30 months, whereas DSAP and AFP lasted for 36 months. Three programmes lasted 60 months: CBFC, ESP and SMART‐Fish; Fadama II lasted 72 months, and GNAEP lasted 99 months. The longest programmes were Fadama III and MAEP, lasting for 111 and 180 months respectively, and IAA, which was identified as having lasted 216 months as the programme covered a long‐term dissemination of integrated agriculture and aquaculture technology. The shortest programme was FoF. Set up as a RCT, the project lasted 22 months, however, this trial has now been expanded under a new name but with no evaluation having yet been completed.

In terms of the aquaculture system the programmes operated, almost all were ponds (*n* = 11). The two programmes that did not operate ponds where CBFC, which used seasonal floodplains, and AFP, which used rice‐fields. Some programmes operated in multiple aquaculture systems, including ponds and rice‐fields (DSAP and GNAEP), and ponds and cages (GNAEP and SMART‐Fish). We were also interested in noting the aquaculture products produced or managed by programme participants. Nine programmes (AFP, CBFC, ESP, Fadama II, Fadama III, FoF, GNAEP, MAEP and SMART‐Fish) focused on different fish species, while SMART‐Fish was the only programme also promoting the harvest of seaweed.

When looking at the specific components along the aquaculture value chain included in these programmes, all had a training activity, except for CBFC and ESP. Training was disseminated in different ways by different programmes. For instance, AFP used farmer‐field schools to provide training on fish storage, harvesting, and pest management; SAFAL trained lead farmers to disseminate practices to other farmers, while SMART‐Fish used demo‐farms to provide training on standard operating practices. Moreover, Fadama II paired training with market support, comprising of improved infrastructure and training specifically on contract negotiation and other skills involved in the sale of the farmer's products.

A distinction can be made between training and technical assistance, as training refers specifically to training days and learning opportunities for beneficiaries, whereas technical assistance refers to long‐term support provided by programmes, most often in the form of extension workers who work throughout the community. In this sense, technical assistance was provided in five programmes (BS, DSAP, FoF, MAEP and SMART‐Fish). For all programmes, apart from SMART‐Fish, this entailed long‐term support from extension workers across the entire length of the intervention. For example, in BS this involved support to beneficiaries as and when needed, while in the case of FoF this support was on an ad‐hoc basis from project staff. SMART‐Fish provided similar long‐term support but also introduced information and communication technologies support with standard operating procedures and guides being disseminated to beneficiaries via posters, books and smart‐phone applications.

Technology provision refers to intervention components which directly provide beneficiaries with the technology to create and rehabilitate aquaculture systems. Every programme included a component dedicated to technology provision. For instance, FoF provided women with the resources to build ponds in their home gardens, and DSAP and IAA provided the technology for beneficiaries to carry out integrated aquaculture‐agriculture production. In turn, direct resources comprises of the provision of aquaculture inputs, for example, fingerlings, fish seed, or other resources which impact production. Direct resources were provided in seven programmes (AFP, CBFC, ESP, Fadama III, FoF, MAEP and SMART‐Fish).

Credit was understood as access to funds or subsidies to improve beneficiary's access along the aquaculture value chain. This component was provided to beneficiaries in six programmes (AFP, BS, DSAP, ESP, GNAEP and MAEP), and this component took different forms. In BS, loans were organised for groups of women to lease ponds, while in ESP subsidies were provided for beneficiaries to purchase fish feed. In addition, mixed‐ and single‐gender fish farmers groups were created in GNAEP, and the women's‐only group was given access to credit.

Postproduction support was a component in five programmes (AFP, Fadama II, GNAEP, SAFAL and SMART‐Fish). This refers to any aspect of an intervention which worked past production along the aquaculture value‐chain and includes, for example, training on trading (AFP) and improved market access/selling conditions (SMART‐Fish).

Finally, we noted if and how each intervention addresses the four outcome pathways, as denoted in our theory of change. In terms of productivity and income, these components are intrinsically linked in the programmes included in the review. As expected, each programme had a component related to aquaculture productivity, which is linked to an increase in income. For example, Alawode and Oluwatayo (2019) make this clear by noting that the theory of change behind Fadama III was that an increase in productivity would positively affect income and other outcomes, such as food security. As described in the previous section on programme components, the main mechanism through which included programmes target productivity is the provision of technology to create and rehabilitate aquaculture systems. Improved aquaculture knowledge through training and best practices was the second most common mechanism, appearing in all but two programmes. The third most common mechanism was the provision of direct resources.

Three programmes had direct nutrition components: SAFAL included a nutrition awareness and knowledge training component in its programme, FoF included a behaviour change communication component targeting nutrition and hygiene, and some beneficiaries of the BS programme participated on a food‐for‐work arrangement, which was additional to the credit and fish production training components of BS.

In turn, five programmes had a direct component related to women's empowerment. The AFP programme encouraged female participants to become “lead entrepreneurs”, those coordinating farmer field schools, and required to have at least one woman lead in each field school. Because of the length of the programme, MAEP transitioned from focusing on the provision of technical inputs to including additional components focused on women's empowerment, which were later also part of GNAEP. Both these programmes promoted women by reserving a proportion of the spaces for women farmers and including gender training and awareness activities. BS solely targeted groups of poor women and aimed to provide them with loans and training on aquaculture production and technology. The FoF programme also only targeted women. While its main focus was on nutrition and productivity, as reflected by the outcomes that were measured among beneficiaries, it also included a behaviour change communication component targeting gender inequality.

### 5.2 Risk of bias in included studies

The risk of bias assessment was used to assess whether aspects of the studies' design, implementation, analysis or reporting may potentially introduce bias into their estimated effects. As mentioned previously, although we have included 21 studies, only 19 papers were included in our synthesis, meaning the risk of bias assessment needed only be completed for these 19 studies. As the assessment is based on the programme level, this means that when a paper reported outcomes on more than one programme, it required assessing the risk of bias individually for each programme within the paper. Because three of our papers reported on two interventions, we have 22 risk of bias assessments in all, 19 from QEDs and three from RCTs.

**Figure 5:**
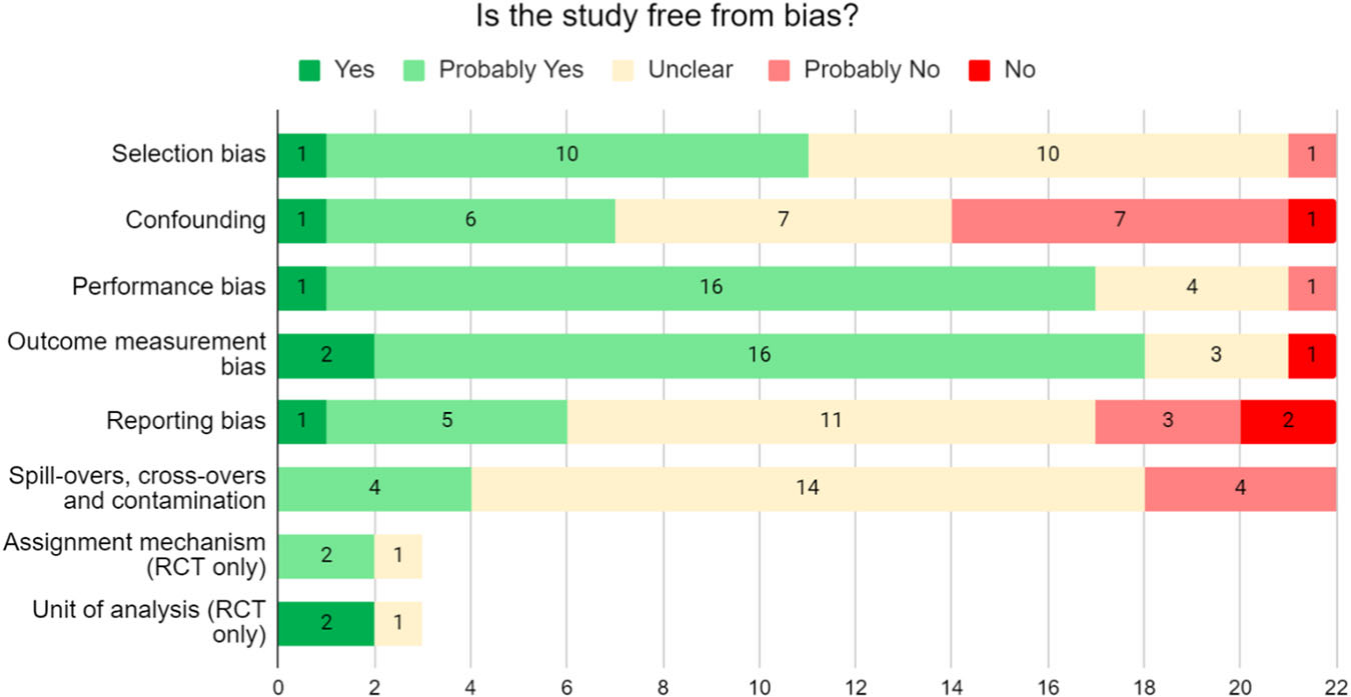
Summary of risk of bias assessment for RCTs and QEDs. A total of 22 risk of bias assessments were conducted, 19 for quasi‐experimental designs, and 3 for RCTs. QED, quasi‐experimental design; RCT, randomised controlled trial

Figure 5 shows the risk of bias assessment categories for all studies. The question being asked in this figure is whether a study is free from a risk of bias, hence the “Yes” and “Probably Yes” answers are positive results, meaning the paper is free from a certain bias. The overall result of the assessment is that, in many cases, studies lacked sufficient detail to make a decision and were classified as “Unclear”. Selection bias refers to whether the treatment allocation or identification mechanism is able to sufficiently control for potential selection bias. For the QEDs, nine studies were probably free from selection bias, nine were unclear, and one study was probably not free from selection bias. The mixed results is also reflected in the RCTs with one study being free from selection bias, one being probably free, and one being unclear. Each of the QEDs used matching to address selection bias, except for Hallman et al. (2003), which used instrumental variables, and Khondker and Pemsl (2011) which used ANOVA.

Confounding bias assessed whether the studies adequately ensured the comparability of treatment and control groups and prevented potential confounding. Five QEDs were probably free from confounding bias, six were unclear while, seven were probably not free, and one was not free. The main reason quasi‐experimental studies performed so poorly in this category is that there was a lack of information regarding which variables intervention and control participants were matched on when using propensity score matching. For RCTs, one study was free from confounding, one study was probably free, and one was unclear. The reason the one study was unclear is that there was no information on the nature of randomisation.

Both the QEDs and RCTs performed well on performance bias, which assessed whether the process of being observed was free from motivation bias. Within the QEDs one was free from performance bias, 13 were probably free, four were unclear, and one was probably not free. Whereas all three RCTs were probably free from performance bias. In most cases there was a lack of information to state there was no performance bias, but there were no obvious signs that it was a problem.

Outcome measurement bias was another example where many papers performed well. Fifteen QEDs were probably free from outcome bias, while three were unclear, and one was not free. Two of the RCTs were free from outcome measurement bias, whereas one was probably free. The paper which was not free from this bias (DANIDA, 2008, for GNAEP) had major issues pertaining to the timing of baseline and endline data collection, as the intervention and control groups had been engaged in the programme for differing amounts of time.

Reporting bias was an issue when papers selectively reported their results. For the QEDs, little information was available, thus we assessed that four papers were probably free from reporting bias, while 10 were unclear, three were probably not free, and two were not free from reporting bias. The two papers which were not free from this bias (Quisumbing & Kumar, 2011, for MAEP and BS) stated in text that results not reported were contradictory to results that were reported in the paper. For the RCTs, one was free from reporting bias, one was probably free, and one was unclear.

Spillovers, cross‐overs and contamination were difficult to assess in the included papers, as there was a lack of information regarding the surveys used to collect data, and there was little information available on the geographic context of programmes. Two of the QEDs were probably free, 13 were unclear, and four were probably not free. For the RCTs, two were probably free, while one was unclear.

The next two biases were only assessed for the RCTs. Two studies were probably free from an assignment mechanism bias, whereas one was unclear. There was little information on how randomisation was conducted, but there was no obvious reason to believe this was an issue. In turn, two studies were assessed as free of unit of analysis bias, while one was assessed as unclear as there was not enough information provided on how SEs were calculated to make a judgement on this issue.

After assessing the bias domains for each included study used in the review synthesis, we categorised each paper with an overall rating for its risk of bias. The full risk of bias assessment for each study can be found in Appendix B. Two papers have an overall low risk of bias, where all bias domains were either yes, or probably yes; both papers covered the FoF programme using an RCT design. Eight papers have “some concern” related to its risk of bias, meaning that one or several domains were unclear and none were no, or probably no. In turn, 12 papers were assessed with an overall high risk of bias, as at least one of the domains was appraised as no or probably no.

### 5.3 Quantitative synthesis of results

The results from the quantitative synthesis is structured following the four main outcome groups of the review: productivity, income, nutrition and women's empowerment. For each of these broad groups, we present all relevant primary and secondary outcomes reported in included studies, as introduced in the previous section. We were unable to identify outcomes which fell outside of these main four outcome groups.

After a first inspection, we found one outlier (standardized score = 5.32) in the full dataset of effect sizes (standardized scores without the outlier ranged from −2.40 to 2.10) However, this effect was ultimately not included in the quantitative synthesis. Where meta‐analysis was possible, the results per outcome include a discussion of moderator, sensitivity and publication bias analyses if applicable.^6^ We assessed 18 moderators to address inequity aspects within the aquaculture context, including substantive characteristics, such as context (continent and country of intervention, exposure to climate shocks index, World Bank country income group) and key intervention features (scale, number of beneficiaries, aquaculture and nonaquaculture components, community or individuals focus, value chain stage, and months of exposure to intervention); methodological characteristics (study design, comparison group, overall risk of bias rating, evaluation period, and if assumptions were made when extracting effect sizes), and extrinsic characteristics of the studies (publication type and year of publication). Complementary information, including plots and tables of these analyses, are included in Appendix C.^7^ If meta‐analysis was not possible given the lack of data reported, we present a narrative description of the outcome.

Some of our included studies presented more than one model specification in their results. Following the review protocol, we extracted data from the authors' preferred model for the only study that explicitly stated one (Alawode & Oluwatayo, 2019). The majority of the studies that provided several specifications used propensity score matching techniques. In these cases, we extracted the nearest neighbour matching technique as this was the most common across studies. While there is a risk that this specific matching technique yielded the more extreme results in these studies, the sensitivity analyses conducted for all relevant outcomes suggest that leaving any of the studies out does not affect the robustness of the results presented in the review.

#### 5.3.1 Productivity outcomes

In this section, we look at the impact of aquaculture programmes on productivity outcomes, in particular on production value and volume. We find a significant positive impact on production value and no statistically significant effect of aquaculture on production volume. The results are based on four and three studies, respectively.

A total of six programmes, reported in six studies, estimated the effect of aquaculture interventions on three productivity outcomes: production value, production volume and production quality. Given the small number of studies informing this outcome group, independent effects were synthesised whenever possible using a traditional approach to meta‐analysis (instead of using RVE, which would not be sufficiently powered to make valid inferences). Below we provide the results on the three outcomes identified.

**Figure 6:**
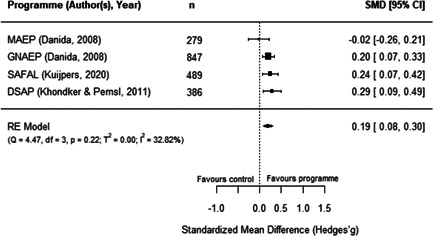
Observed outcomes and average effect for production value

Figure 6 reports the four programmes including outcomes on production value. The random‐effects model shows a positive and statistically significant estimated average effect of 0.19 SDs (95% confidence interval [CI] [0.08, 0.30], *p* < .01) and a low level of heterogeneity (*I* ^2^ = 32.82%). The prediction interval (not shown in the figure) ranges from 0.02 to 0.36 SDs, meaning that a random new aquaculture intervention would have a positive predicted effect on production value.

Further sensitivity analyses indicate the absence of outlier or influent studies for this outcome, showing robust results when leaving each study out of the analysis. Moreover, we conducted moderator analyses for the effect of aquaculture interventions on production value to account for any drivers of the overall estimate; however, these analyses were either not powered due to the small number of studies available, or were not statistically significant under conventional levels (*p* < .05). While the risk of bias of these studies is not a significant moderator of the average effect, two of these studies have an overall high risk of bias (reports for the GNAEP and DSAP programmes), suggesting that we should interpret this result with some caution.

**Figure 7:**
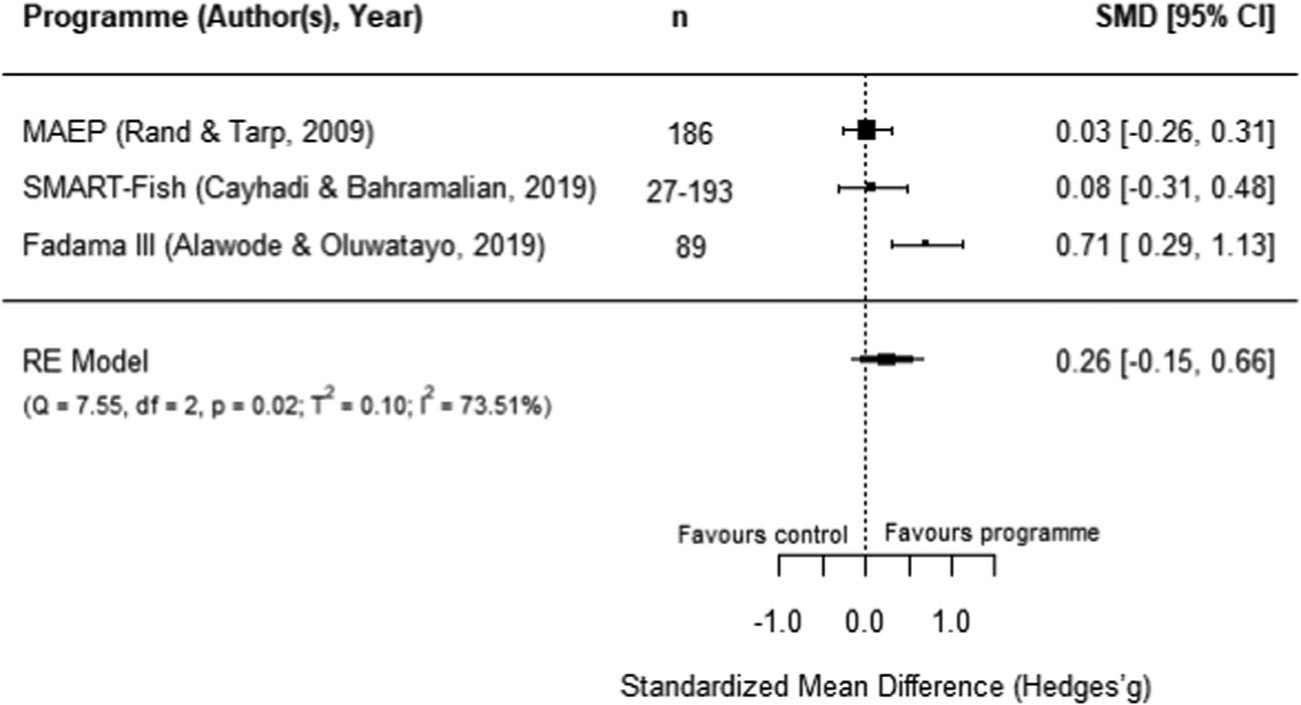
Observed outcomes and average effect for production volume

As shown in Figure 7, three studies reported outcomes related to the production volume^8^ from aquaculture programmes. The meta‐analysis shows a nonstatistically significant estimated average effect (SMD = 0.26, 95% CI [−0.15, 0.66], *p* = .22) with a considerable level of heterogeneity (*I* ^2^ = 73.51%). This suggests that a random new aquaculture intervention could have a positive, null, or negative effect on the level of production volume (prediction interval [−0.47, 0.99]; not shown in the figure).

While the estimate for Fadama III (Alawode & Oluwatayo, 2019) seems higher than that of the other two programmes, leaving this study out of the analysis results in a lower estimate but still within the confidence interval of the main effect, suggesting that this study is not overly influential. The lack of studies reporting effects on production volume does not allow for further moderator analyses. Moreover, the three studies reporting this outcome have a high risk of bias, and as such, this finding should be interpreted with caution.

Finally, the SMART‐Fish programme (Cahyadi & Bahramalian, 2019) estimated the effect of the intervention on production quality, measured by the colour spectrum of the products using a 1–4 scale. The report presents a positive but not statistically significant effect for production quality (SMD = 0.18, 95% CI [−0.13, 0.48]), and the study was assessed as having a high risk of bias.

#### 5.3.2 Income outcomes

In this section we look at the impact of aquaculture interventions on a number of income and livelihood measures. We first look at an aggregate livelihood construct, and then we synthesise specific outcomes including income, household total and food expenditure, poverty, household assets, and farm profit. We only find a significant impact on income and total household expenditures, based on 10 and five studies each.

A total of 16 studies reported outcomes related to the income group for 12 of the included programmes. Using RVE, we first synthesised similar outcomes representing different means of support that could be affected by being involved in aquaculture activities, which we refer as a livelihood construct. Twelve programmes contributed a total of 53 effects to this analysis, all of which relate to income measures at the farm, household, or farmer level, and expenditure and profit measures related to aquaculture activities. An RVE model was used solely for this outcome, as this is the only outcome group for which we had a sufficient number of effect sizes (and thus sufficient power for an RVE model).

The analysis reports a positive and statistically significant effect of aquaculture interventions on this livelihood construct (SMD = 0.25, 95% CI [0.13, 0.38], *p* < .01), which shows a high level of heterogeneity (*I* ^2^ = 86.68%). Moderator analyses suggest that this effect is higher by 0.10 SDs when aquaculture interventions are implemented in more than one region, rather than in only one region or at the national level, and when programmes focus on the provision of supplies and activities before production (*p* = .05 for both estimates). However, this effect is lower for interventions that include a nonaquaculture component and when they have an explicit focus around women's empowerment, by 0.13 and 0.19 SDs respectively, compared to when interventions do not include such components (*p* = .04 for both estimates).

As this analysis does not allow to differentiate patterns across outcomes and potentially between subgroups, we also synthesised the effect of aquaculture interventions on individual outcomes related to the income group. Below we discuss these results for the following nine outcomes reported in included studies: income, total and food expenditures, farm profit, household assets, poverty incidence, market participation, revenue and prices.

**Figure 8:**
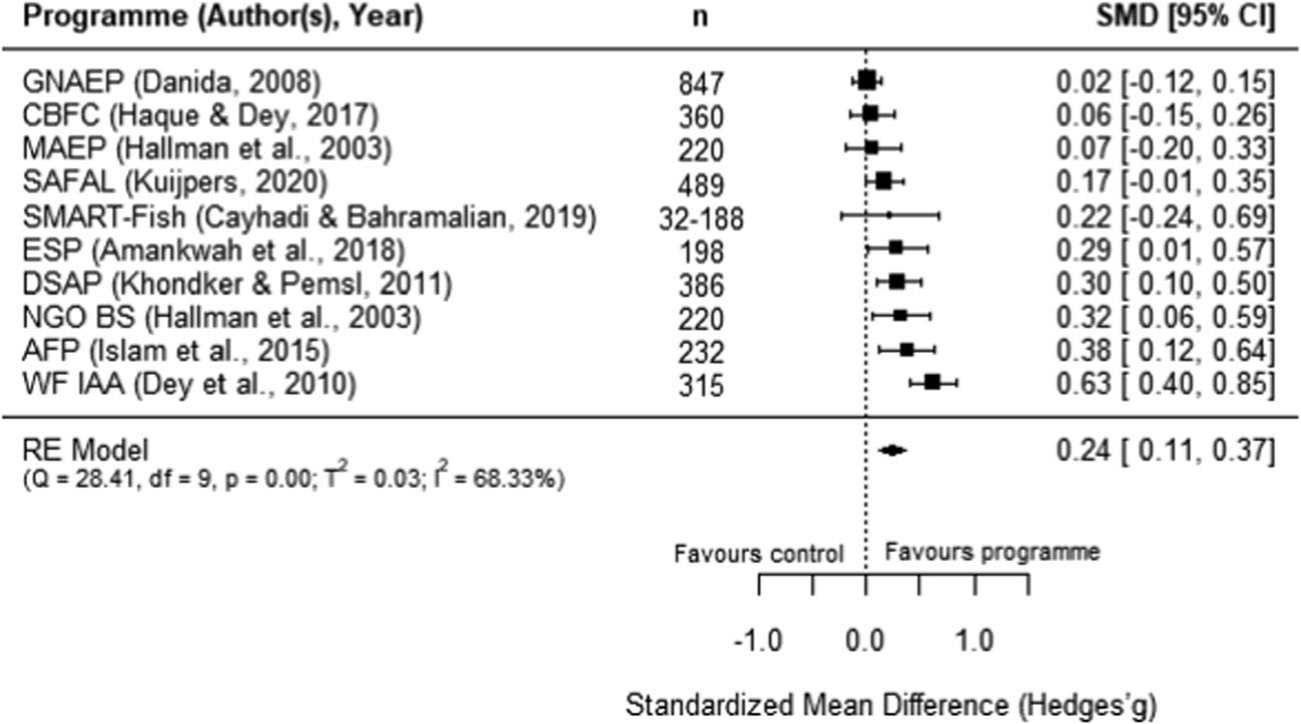
Observed outcomes and average effect for income

Figure 8 presents the 10 programmes that report an income outcome.^9^ The average effect of aquaculture interventions on income at the farmer, household, or farm level is estimated to be 0.24 SDs (95% CI [0.11, 0.37], *p* < .01) and presents a relatively high level of heterogeneity (*I* ^2^ = 68.33%). Moreover, the prediction interval for this outcome crosses the line of no effect ([−0.11, 0.58]; not shown in the figure), suggesting that a random new aquaculture intervention could have a positive, null or negative effect on income.

The effect of WorldFish's intervention in Malawi (Dey et al., 2010) seems to be a potential outlier in this analysis; however, sensitivity analyses show that when excluding this study, the average effect on income remains substantively and statistically the same. In turn, moderator analyses indicate that the effect on income is lower by 0.31 SDs for countries with a “very high” exposure to climate shocks, which in this case coincides with interventions in Asian countries (*p* = .02 for both estimates). This would indicate that the impact on income would be driven by the two aquaculture programmes based in African countries (ESP and particularly IAA). Likewise, the effect on income is lower by 0.26 SDs for programmes located in Bangladesh (*p* = .04), compared to interventions in other Asian or African countries. Moreover, the effect on income is reduced with longer exposures to the intervention (*p* = .02); however, this difference is substantively small, accounting for almost a 4% reduction in the effect for every additional year of exposure to the intervention. Finally, although the risk of bias of this set of studies is not a significant moderator of the average effect, seven of the 10 studies included in this analysis have a high risk of bias; hence, this result should be interpreted with some caution.

**Figure 9:**
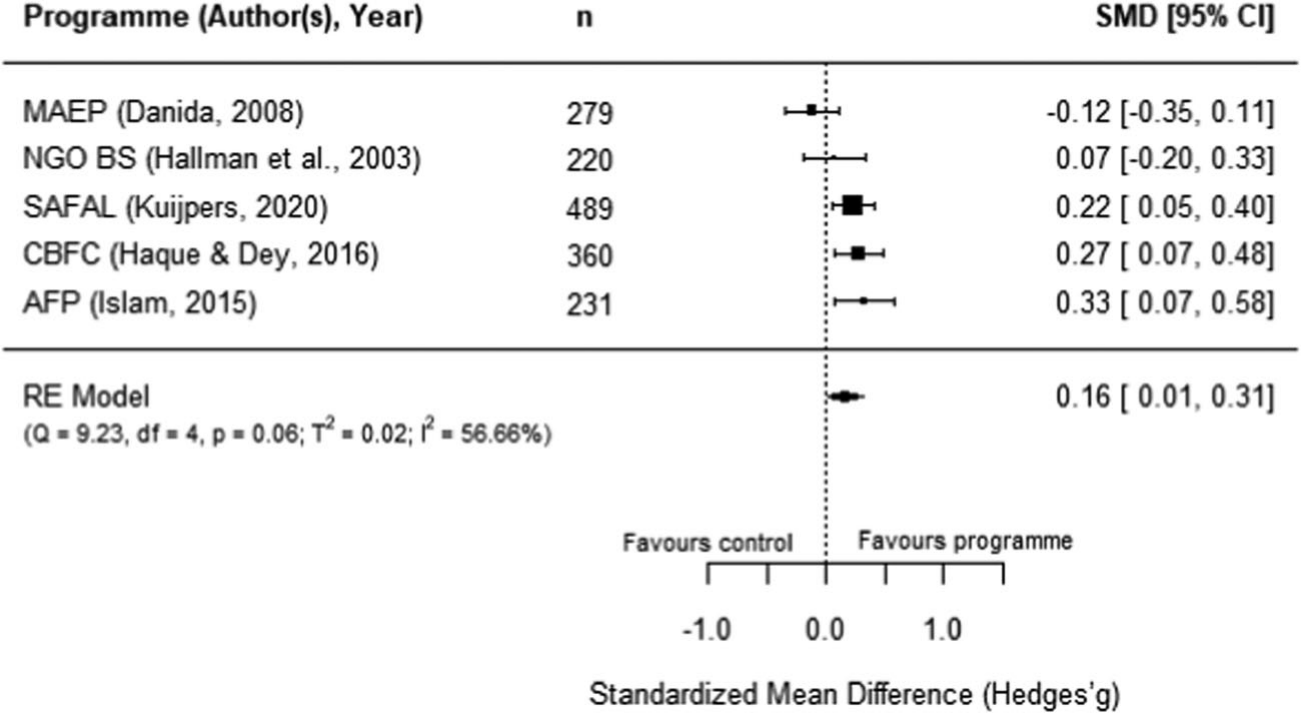
Observed outcomes and average effect for total expenditures

As presented in Figure 9, total expenditures at the household or farm level was reported for five programmes. The meta‐analysis shows a positive and statistically significant average estimated effect of 0.16 SDs (95% CI [0.01, 0.31], *p* = .04), and some heterogeneity although not significant (*I* ^2^ = 56.66%). We conducted moderator analyses to account for any drivers of the overall effect and found that the impact of aquaculture interventions is lower for programmes with a larger number of beneficiaries and when the effect was measured further away from the end of the programme (*p* = .01); however, for both variables, the magnitude of this effect is substantively insignificant, accounting for differences of 0.000 and 0.005 respectively. The sensitivity analysis suggests a robust estimation, with practically no statistical or substantive variation when excluding any of these studies from the analysis. While the overall risk of bias of these studies is not a significant moderator of the average effect, only one of these five studies has a high risk of bias (Hallman et al., 2003).

**Figure 10:**
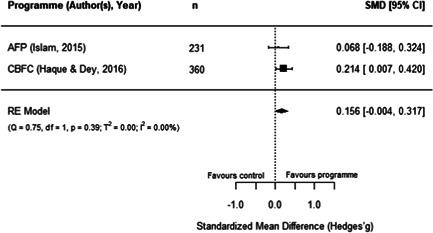
Observed outcomes and average effect for food expenditures

In turn, only two programmes reported the effect of aquaculture interventions on household food expenditures. Figure 10 shows that the average effect just crosses the line of not effect (SMD = 0.16, 95% CI [−0.004, 0.317], *p* = .056) and presents no heterogeneity (*I* ^2^ = 0.00%), suggesting that the overall effect on household food expenditures statistically equal to zero. Further statistical moderator, sensitivity, or publication bias analyses are not possible for this outcome, as it is only informed by two studies. Both studies were assessed as having some concerns regarding their overall risk of bias.

**Figure 11:**
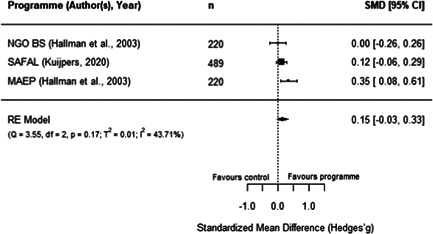
Observed outcomes and average effect for farm profit

As presented in Figure 11, three programmes included farm profit in their outcomes. Our results show a nonsignificant average effect of aquaculture interventions on farm profit levels (SMD = 0.15, 95% CI [−0.03, 0.33], *p* = .10), with a low amount of heterogeneity (*I* ^2^ = 43.71%). While the estimate for MAEP (Hallman et al., 2003) is higher than that of the other two programmes, leaving this study out of the analysis results in a lower estimate but still within the confidence interval of the main effect, suggesting that this study is not overly influential. The lack of studies reporting effects on farm profit does not allow for further moderator analyses. Moreover, Hallman et al.s' report (2003), covering the MAEP and BS programmes, was assessed as having an overall high risk of bias, so this finding should be interpreted with caution.

**Figure 12:**
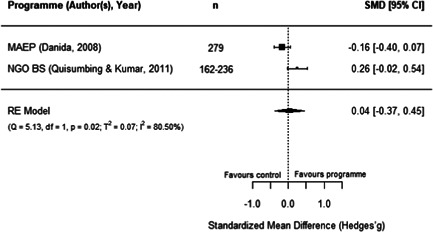
Observed outcomes and average effect for household assets

Two programmes reported outcomes related to a range or combination of household assets. Figure 12 shows that the impact of aquaculture interventions on household assets crosses the line of no effect (SMD = 0.04, 95% CI [−0.37, 0.45], *p* = .85), with a large and significant level of heterogeneity (*I* ^2^ = 80.50%). However, further statistical moderator, sensitivity, or publication bias analyses are not possible for this outcome, as it is only informed by two studies. Moreover, one of these two studies has an overall high risk of bias (Quisumbing & Kumar, 2011), while the other study was assessed as having some risk of bias concerns.

**Figure 13:**
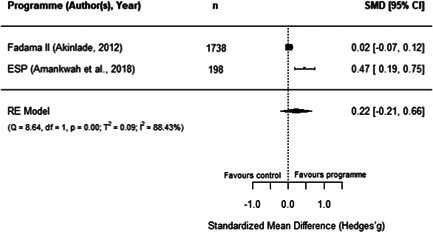
Observed outcomes and average effect for poverty incidence

Likewise, only two programmes estimated the effect of aquaculture interventions on poverty incidence. This outcome was reversed before conducting the analysis; hence, a positive average effect would indicate that the programmes had a positive impact on reducing poverty incidence. The meta‐analysis, presented in Figure 13, shows that the estimated average effect crosses the line of no effect (SMD = 0.22, 95% CI [−0.21, 0.66], *p* = .31) and presents a considerable level of heterogeneity (*I* ^2^ = 88.43%). Because poverty incidence is only reported for two programmes, further statistical moderator, sensitivity, or publication bias analyses are not possible for this outcome. Moreover, both studies have an overall high risk of bias. Additionally, ESP estimated the effect of the programme on poverty incidence of two subgroups: small farms versus large farms,^10^ and farmers with less than versus farmers with at least high school education. These effects (SMDs) range from 0.16 to 0.34 and only the effect within small farms is statistically significant. In the same line, Fadama II also estimated the effect of the programme on the poverty gap index and the severity of poverty index. However, the study only reported levels of significance and we had to assume a *t* value (as described in the Section Results, 4). This meant that all effects with the same level of significance received identical *t* values, and thus identical effect sizes.

Finally, only the SAFAL programme reported an effect on farm revenue and market participation. The former was measured as “total yearly earnings from the sale of crops, livestock products, and aquaculture based on the actual received prices instead of median prices” (Kuijpers, 2020, p. 9), whereas market participation was defined as “the gross value of farm sales divided by the gross value of all products produced by the farm” (Kuijpers, 2019, p. 17). While both studies have some concerns surrounding their overall risk of bias, the SAFAL programme shows positive results for both outcomes (farm revenue: SMD = 0.22, 95% CI [0.04, 0.39]; market participation: SMD = 0.23, 95% CI [0.06, 0.41]). In turn, the SMART‐Fish programme (Cahyadi & Bahramalian, 2019) is the only study reporting outcomes related to prices, measured as the received prices per different aquaculture products. These effects are not statistically significant with SMDs ranging from −0.02 to 0.57, and this study was assessed as having a high risk of bias.

#### 5.3.3 Nutrition outcomes

In this section, we explore the impact of aquaculture programmes on a range of indicators related to nutrition. We find positive and significant effect on food consumption, although the analysis is based on only two studies. In line with the literature, we do not find a significant impact on any of the anthropometric measures reported in our included studies.

A total of six programmes, reported in six studies, estimated the effect of aquaculture interventions on a range of outcomes related to nutrition, including fish consumption, anthropometric measures for women, men, and children, food security, micronutrients intake, and blood concentration measures. Because of the small number of studies reporting outcomes within this group, we synthesised independent effects using a traditional approach to meta‐analysis (instead of using RVE) whenever possible. Below we present the results on the nutrition outcomes identified.

**Figure 14:**
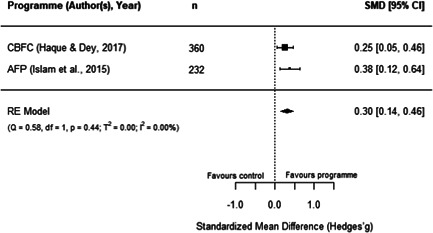
Observed outcomes and average effect for fish consumption

Two programmes report outcomes related to fish consumption within participating households. As shown in Figure 14, the average effect of aquaculture interventions on fish consumption is positive and statistically significant, estimated as 0.30 SDs (95% CI [0.14, 0.46], *p* < .01) with no heterogeneity (*I* ^2^ = 0.00%). Because we only identified two programmes reporting this outcome, further statistical moderator, sensitivity, or publication bias analyses are not possible for fish consumption. Both studies have some concerns related to their overall risk of bias.

**Figure 15:**
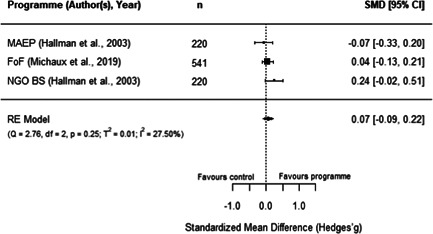
Observed outcomes and average effect for women's body mass index

Figure 15 presents the three programmes that report the effect of aquaculture interventions on women's BMI. Our analyses show a statistically nonsignificant average estimated effect (SMD = 0.07, 95% CI [−0.09, 0.22], *p* = .40), with a low level of heterogeneity (*I* ^2^ = 27.50%), which indicates that a new random aquaculture programme may have a positive, null, or negative effect on the BMI of adult women (prediction interval [−0.14, 0.27]; not shown in the figure). While the estimate for the BS programme (Hallman et al., 2003) is higher than that of the other two programmes, leaving this study out of the analysis results does not change the average effect statistically or substantively. The lack of additional data does not allow for further moderator analyses. While FoF's report (Michaux et al., 2019) has a low risk of bias, Hallman et al.s' (2003) report has an overall high risk of bias.

**Figure 16:**
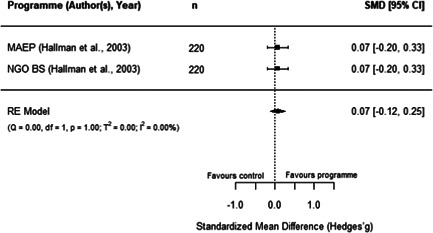
Observed outcomes and average effect for men's body mass index

Additionally, as shown in Figure 16, the MAEP and BS programmes (Hallman et al., 2003) also report the BMI of men. Due to missing data in this report, we had to make assumptions to extract these outcomes and hence, the individual effects for both programmes is the same. The average estimated effect crosses the line of no effect (SMD = 0.07, 95% CI [−0.12, 0.25], *p* = .48), with has no heterogeneity (*I* ^2^ = 0.00%). As there is no variation in these two effects, we did not conduct moderator, sensitivity, or publication bias analyses. Moreover, Hallman et al.'s report (2003) has a high risk of bias, so this finding should be interpreted cautiously.

**Figure 17:**
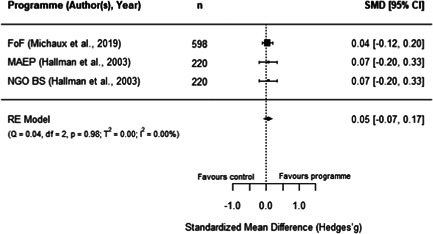
Observed outcomes and average effect for children's height‐for‐age

Similarly, three programmes report the effect of aquaculture interventions on height‐for‐age of children 0–5 years old. Figure 17 shows that the average effect also crosses the line of no effect (SMD = 0.05, 95% CI [−0.07, 0.17, *p* = .40), and presents no heterogeneity (*I* ^2^ = 0.00%). Sensitivity analyses show robust results when excluding any of these studies.

In addition, Hallman et al. (2003) disaggregated height‐for‐age for boys and girls under 5 years old, showing nonstatistically significant effects for both groups (girls: SMD = 0.07, 95% CI [−0.12, 0.25]; boys: SMD = 0.00, 95% CI [−0.19, 0.19]). In the same line as previous anthropometric outcomes, these findings should be interpreted with caution given that Hallman et al.'s (2003) study has a high risk of bias. In turn, the FoF programme (Michaux et al., 2019) also reported measures for the weight‐for‐height and weight‐for‐age, with both outcomes showing a negative and nonstatistically significant effect for participating children (due to reporting issues, both outcomes have the same effect size: SMD = −0.04, 95% CI [−0.15, 0.07]). This report was assessed has having an overall low risk of bias.

Two programmes reported food security measures. For SAFAL, Kuijpers (2020) reported the length of the hungry season of participants, showing a positive a statistically significant effect (SMD = 0.20, 95% CI [0.03, 0.38]). In turn, for FoF, Talukder and Green (2014) reported a food insecurity score. However, the sample in this study was not clearly reported, so we could not calculate an effect size with confidence. Therefore, this outcome could not be quantitatively synthesised. Moreover, both studies have some concerns surrounding their overall risk of bias.

Finally, FoF is the only included programme reporting micronutrients intake and blood concentration measures of mothers and children aged 5 or younger. Verbowski et al. (2018) reported several intake and inadequate prevalence measures, including energy, protein, fat, iron, zinc, calcium, vitamin A, thiamin and riboflavin for participating mothers and children of this RCT. Due to incomplete data in the report, we had to make assumptions to extract the results; therefore, many of these outcomes have the same effect sizes, which hinders the comparability of results. However, the report presented mixed and some statistically significant findings: the 17 effects reported for mothers range between −0.29 and 0.29 (SMD) where five of them are statistically significant, while the 17 effects reported for children range from −0.06 to 0.06 where none of them are statistically significant.

Likewise, Michaux et al. (2019) reported a range of blood measures as outcomes related to this programme, including serum ferritin, serum zinc, soluble transferrin receptor (sTfR), retinol binding protein, C‐reactive protein, α‐1 acid glycoprotein (AGP), and Hb concentrations, as well as anaemia rates for nonpregnant women. Children up to 5 years old were also measured for Hb and anaemia. The report did not include clear samples sizes for the two outcomes measured on children and for serum zinc concentration on nonpregnant women. The other seven measures yielded a nonstatistically significant effect (SMD = 0.04, 95% CI [−0.04, 0.12]). In addition, one study covering FoF (Talukder & Green, 2014) also measured the only secondary outcome included in the review, which lies under the nutrition pathway: vegetables production within participating households. While the paper did not report clear sample sizes for this analysis, it described a higher production of vegetables for treated households with homestead gardens. The first two of these studies have an overall low risk of bias, while the third study was assessed as having some risk of bias concerns.

#### 5.3.4 Women's empowerment outcomes

Due to the paucity of rigorous impact evaluations related to women in the aquaculture sector we were unable to conduct a quantitative analysis of the extant literature. Instead, this section synthesises in a narrative way the programmes measuring the effect of aquaculture interventions on women's empowerment outcomes. These programmes are also identified following Johnson et al.'s (2018) framework to understand interventions on female empowerment, as discussed in the Background section.

While we are not able to draw many comparisons across studies due to differences in the measures used, we were able to identify several trends. These studies highlight the importance of the context, particularly the local gender norms, and the implementation approach of the programmes to explain the findings on women's empowerment indicators. Below we provide a description of these results, and Table 3 presents a summary of the measures and findings for each programme.

**Table 3:** Summary of measures and findings for women's empowerment outcomes

Study	GNAEP	BS	MAEP
Hallman et al. (2003)	‐	Used 19 binary indicators on mobility, control, domestic violence, and political knowledge. The 17^a^ effect sizes (SMDs) reported range from −0.07 to 0.38, where 53% are positive effects. Positive results in terms on physical mobility (increased attendance to NGO sessions, and working for pay)	Used 19 binary indicators on mobility, control, domestic violence, and political knowledge. The 18^b^ effect sizes (SMDs) reported range from −0.38 to 0.38, where 78% are positive effects. Mixed results regarding political knowledge and activity. Positive results in terms of control over resources (less likely to have assets taken away)
Quisumbing and Kumar (2011)	‐	Used 22 indicators on wife/husband's growth in owned land and assets. The 22 effects sizes (SMDs) reported range from −0.35 to 0.35, with 45% of positive effects. Women's assets increased more relative to their husbands, suggesting a decrease in gender asset inequality	Used 22 indicators on wife/husband's growth in owned land and assets. The 22 effects sizes (SMDs) reported range from −0.47 to 0.47, with 14% of positive effects. Increase in husband's ownership of assets and land, relative to their wives, suggesting an increase in gender assets inequality
DANIDA (2008), Annex 9	Used a 0–7 women's empowerment index measuring autonomy and decision‐making. There is a positive and statistically significant effect (SMD = 0.46, 95% CI [0.33, 0.58]), suggesting an increase of around 1 SD from the index's mean value	‐	Used a 0–10 women's empowerment index measuring autonomy. No statistically significant effect was found (SMD = 0.15, 95% CI [−0.14, 0.43])

^a^ Two of the 19 binary indicators are reported as “NA.”

^b^ One of the 19 binary indicators is reported as “NA.”

Three of the five programmes including an intervention component related to women's empowerment report a range of different outcomes related to women's autonomy, decision‐making, and assets ownership. However, included studies for the AFP and FoF programmes did not report women's empowerment‐related outcomes. These programmes could then be classified as reaching women, as they aimed to promote female participation but, without further information, it is unclear whether they were benefited from these interventions.

The three programmes included in this synthesis were all located in Bangladesh and were covered by three manuscripts. Hallman et al. (2003) studied the BS and MAEP programmes, using 19 binary indicators covering several different aspects of empowerment, such as physical mobility, control over resources, domestic violence, and political knowledge and activity. Quisumbing and Kumar (2011) covered the same two programmes, reporting 22 indicators on the differential change and growth between wife and husband's owned land and assets. In turn, DANIDA (2008, Annex 9) covered the GNAEP and MAEP interventions, using a similar—although not equal—women's empowerment index. For the MAEP programme, the index measured only freedom of movement using a 0–10 scale, and included indicators on whether women were able to go outside the village, to the bazaar, hospital/doctor, cinema/fair, or to training sessions. The index for the GNAEP intervention used a 0–7 scale and included these autonomy indicators plus additional decision‐making indicators related to aquaculture production, health care, education, and access to credit choices. Although these indices are presented in the same report, there is no mention as to why the analyses for these two programmes use a different measure of the women empowerment index.

While the outcomes related to women's empowerment reported in these studies varies greatly, we identified an additional challenge to combining these measures. Hallman et al. (2003) and Quisumbing and Kumar (2011) reported their findings in a way that was not possible to translate them into effect sizes without making some statistical assumptions. Particularly, these studies reported the estimated coefficients and their corresponding *p* values (either numerically or by denoting the significance level using stars); however, without the SEs and/or *t* values associated with the coefficients, we had to assume the value of *t* following our methodological protocol (see Section Results, 4). These assumptions meant that the estimated effect sizes for these studies had little variability. Additionally, Hallman et al. (2003) did not report clear analysis samples for all relevant outcomes. Given the high number of individual indicators reported in these studies, a quantitative synthesis would not be addressing adequately the whole range of indicators. Therefore, this outcome group would be better addressed narratively. The rest of the section describes the main findings by programme.

According to Quisumbing and Kumar (2011), MAEP's extension programme increased the ownership of land and assets for the husbands, relative to that of their wives, suggesting that MAEP could have increased the existing gender asset inequality among participants. The authors linked this result to the fact that MAEP was implemented in an area of Bangladesh under more conservative gender norms; hence, by default, women tended to have less control over resources and involvement in work‐related activities. The report also highlighted that MAEP's individual approach in the dissemination of the programme meant that it was really targeting husbands. To summarise the importance of local gender norms in the implementation and expected results of a programme, the authors noted that “these norms do not change overnight and attempts to directly challenge such norms—such as involving women directly in the marketing of agricultural produce in areas where female seclusion is valued—may unintentionally result in an erosion of women's claims to resources” (p. 237). Likewise, Hallman et al. (2003) also noted that cultural and gender conservativism was an important factor to consider in the case of MAEP. Moreover, practical aspects of the intervention may not have necessarily helped address these issues. For example, they mention that “the ponds are largely outside the household compound, making it more difficult in practice for women to operate them” (p. 43). The authors agreed that, even though MAEP had an explicit aim to target women, in practice it was the husbands who operated the ponds. Given this, it is less surprising that this analysis found few significant effects related to women's empowerment: in terms of political knowledge and activity, participating women would be more likely to know the name of the prime minister but would also be more influenced by others when making electoral decisions. In turn, women participating in MAEP would be less likely to have their assets taken away by her husband or another family member, compared to nonparticipating women. Lastly, the DANIDA (2008) report found nonsignificant effects on the women's empowerment index, which measured indicators or mobility. Under Johnson et al.'s (2018) framework, MAEP would have the potential to benefit, or even empower women; however, due to contextual factors, this potential was difficult to accomplish.

Quisumbing and Kumar (2011) also reported that, relative to their husbands, assets and land ownership increased more for women participating in the BS programme. As this finding was not identified for MAEP participants, which applied the same aquaculture technology, the authors expanded on the idea that the implementation approach may be key to the success of programmes introducing new technologies. In the case of BS, the focus of the implementation was on groups of women managing the aquaculture ponds, and the authors argue that the social capital built in the short term within these groups could have been the channel to identify positive effects in the level of assets ownership of these women. This argument would also be supported by Hallman et al.'s (2003) study, which found positive impacts of the BS intervention on indicators of women's mobility. The authors report that technology‐adopting women showed higher attendance rates to training and project sessions run by the implementing NGO, as well as an increased likelihood of having a paid work, compared to likely adopters of the programme. This suggests that BS could be classified as benefitting women, possibly through the channel of social capital, but it remains unclear if this positive effect could be translated into empowerment.

In turn, the DANIDA report (2008) justified the use of a women's empowerment based on indicators of mobility and decision‐making on the notion that an increased control over these aspects could ultimately influence other welfare areas, such as education or nutrition‐related decisions for them and their families. The study found a positive average effect of GNAEP participation, indicated by an increase of 1 SD from the women's empowerment index mean. In line with the other two studies covered in this section, the DANIDA report also mentioned the importance of the dissemination approach of these programmes. While the authors note that this is a suggestive link, they speculate that, in contrast to the individual approach of MAEP, the household approach of GNAEP in which both the husband and wife attended the programme sessions could be related to the effects identified on women's empowerment indicators. These findings would signal that GNAEP may have the potential to empower its women beneficiaries, possibly by adapting the intervention mechanisms to the local context.

All these studies and programmes combinations have an overall high risk of bias rating, with the exception of the DANIDA report for the MAEP programme, which presents more information than for the GNAEP programme and, hence, has been assessed as having some concerns related to its risk of bias. While the narrative synthesis of these results provides some interesting insights, these findings should be interpreted with caution.

### 5.4 Summary of findings from qualitative evidence

This section details the evidence we found in answer to review question 4: What are the potential barriers and facilitating factors that impact the effectiveness of aquaculture interventions? As stated previously, we conducted a separate search for additional documentation to address this question. The search led to the identification of an additional 21 documents, which provided descriptive and qualitative data pertaining to barriers and facilitators of the effectiveness of the 13 programmes included in our review. Additionally, eight of the included impact evaluation papers contained information for barriers and facilitators, leading to the identification of a total of 29 papers for this analysis.^11^ Appendix A.7 includes the full list of these documents.

From the analysis conducted, three clear dimensions emerged: barriers and facilitators which affected programme set up, participation of beneficiaries, and the level of productive aquaculture activities. On programme set up, the key barriers identified related to low funding, participants not being able to choose the intervention package, unclear roles of partners, and plans that were never implemented. On participation, the main themes were social and cultural norms, the income from aquaculture activities, programme delivery features, and access to natural capital. On productive activities, we identified access to inputs and funding, the general economy, infrastructure, and environmental issues. While many of these themes were relevant across programmes, the analysis presented in this section should be taken as suggestive evidence and interpreted with some caution as we are unable to make causal claims from them.

#### 5.4.1 Process and quality of the qualitative evidence

After identification, we categorised the 29 papers used for the analysis of barriers and facilitators into four groups. The first category contains impact evaluation papers included in our quantitative synthesis which do not use other qualitative data collection or analysis techniques (Alawode & Oluwatayo, 2019; DANIDA, 2008; Dey et al., 2010; Khondker & Pemsl, 2011; Michaux et al., 2019; Rand & Tarp, 2009; Talukder & Green, 2014). These seven papers provided information on the programmes, usually based on authors' interpretations rather than on stakeholders' perspectives. The second category consists of papers that utilised quantitative research techniques but did not meet our eligibility criteria and, as such, were excluded from the review (Bature et al., 2013; Bouis, 2000; Fadare & Adereti, 2017; Kioi, 2014; Kiwiri & Njeru, 2015; Kumar & Quisumbing, 2011; Njagi et al., 2013; Olaoye et al., 2011; Ovharhe, 2020; Pant et al., 2014). Each of these 10 papers provided information on programme implementation. Many collected data using surveys and presented descriptive statistics on successes and failures from the perspective of beneficiaries and other stakeholders. The third category contains reports of one or multiple programmes (FAO, 2009; Meizen‐Dick et al., 2003; Sheriff et al., 2010; WorldFish, 2009). Rather than conducting data analysis, these four papers summarised the characteristics of included programmes and/or the analysis results conducted in other related studies (FAO, 2009; Meizen‐Dick et al., 2003; Sheriff et al., 2010; WorldFish, 2009). Finally, the fourth category consists of papers that utilised qualitative or mixed‐methods research techniques to explore the views of programme stakeholders (Hallman et al., 2003; Hima et al., 2016; Kessler et al., 2017; Mandal et al., 2014; Moumin, 2016; Naved, 2000; Omobowale & Akinola, 2017; UNIDO, 2019). Because these eight papers tried to achieve qualitative goals (i.e., understand the first‐hand experience of participants), they were critically appraised to provide a sense of their trustworthiness in terms of how the studies were contextualised, conducted, analysed, and presented. Appendix A.8 presents both the appraisal tool used and the detailed results for each paper.

These eight papers clearly described the research aims and the context in which the studies took place. All of them intended to use appropriate qualitative data collection techniques sourced from suitable participants. All these papers were also successful in providing enough evidence to support their findings, which meant that all research questions were addressed. However, none of these studies reported having quality data checks before analysis, or provided a reflection on the study's weaknesses and the researcher's bias and positionality. Additionally, only one study provided a strong description of the sampling procedure, data collection, and data reporting techniques used. One of the eight studies provided a well‐developed discussion of how their findings connected with theory, literature, or practice, and only one study reported ethical considerations related to their research.

In terms of the whole set of papers used in the analysis of barriers and facilitating factors, the substantive nature of these documents varies greatly. While some papers explore the entire programme, many papers speak only to a certain region or aspect of a programme. For instance, the additional documents related to the Fadama II and Fadama III programmes only looked at the beneficiary perspectives in certain states. In the same line, some programmes worked across multiple sectors (i.e., not exclusively in aquaculture; e.g., ESP, Fadama II, Fadama III, and SAFAL), and in some cases, the papers may have included the views of beneficiaries and stakeholders from other sectors. Thus, the conclusions from these papers may not have reflected the implementation of a programme in all locations, or the implementation of the aquaculture arm in multisectoral programmes. Moreover, implementation documents were also not representative of the entirety of a programme's length, with many of the implementation reports that we identified assessing the midterm achievements of the programmes. In all, the following discussion should be taken as suggestive and as an exploratory analysis of barriers and facilitators. The documents required to take a more in‐depth analysis were not available and so, again, the paucity research being produced around aquaculture interventions and programmes is apparent.

From the additional documents we were able to identify, several different themes or findings emerged, which were grouped into three distinctive dimensions: barriers and facilitators which affected the programmes' set up, the participation of beneficiaries, and the productive activities along the aquaculture value chain. In the following sections, we discuss each of these themes in turn. Additionally, each theme is summarised by a table including example quotes. These illustrate how the theme applies in the instance of one specific programme; we then discuss how the theme is covered in different interventions.

#### 5.4.2 Factors affecting programme set up

Table 4 shows the four themes we were able to identify as factors which affected programme set up. Overall, there was little available evidence on these factors, which is reflected in the number of programmes covered by each finding.

**Table 4:** Summary of findings for factors affecting programme set up

Theme and programmes reporting this finding	Evidence example	Papers contributing to this finding
**Low funding** Fadama III	“These lessons relate mostly to the challenge of scaling up the programme to the national level amid uneven performances among communities. Because the programme has been mainstreamed across local governments but not scaled up financially, the nationwide rollout has significantly stretched the programme's capacity and diluted available resources across a much larger number of beneficiaries. Although there was some effort to reinforce the capacity of beneficiaries who had received support under previous phases, Fadama III purposefully selected new villages that had not been treated under the second phase. This left many of the beneficiaries of Fadama II disappointed” (Hima et al., 2016, p. 18).	Hima et al. (2016)
**Production plans never implemented** BS GNAEP	“In Jessore, in four of nine group ponds surveyed, production was never planned and undertaken by the NGO‐sponsored groups themselves. In two of these four cases of non‐operation, excavation of ponds was not undertaken at all or was inadequate” (Bouis, 2000, p. 483).	Bouis (2000); FAO (2009); Kumar and Quisumbing (2011)
**Randomisation decreased motivation** FoF	“There were also challenges to the project and many lessons learned. Randomisation itself may have led to some farmers being less committed than if they had chosen the intervention package themselves. When a new factory offered work across the border in Thailand, many women chose to leave their farms and families for cash employment reducing the sample size. We recognize that motivational factors and aspirational beliefs need to be better understood for developmental outcomes to be achieved” (Talukder & Green, 2014, p. 5).	Michaux et al. (2019); Talukder and Green (2014)
**Unclear roles of partners** GNAEP	“In implementation it was reported that the roles of DoF, the NGOs, and DTA were not sufficiently clear and in reality roles were [not] followed according to the plan. Project reports state that the NGOs actually took on a technical role while DTA operated as the “main facilitator” for capacity building. It was also reported that DoF was not able to undertake the role articulated in project planning documents. In addition Community Based Organisations were established and took on roles of distributors of prawn post larvae and feed” (DANIDA, 2008, Annex 4, pp. 17–18).	DANIDA (2008), Annex 4

Fadama III was identified as the only paper which was affected by low funding. As the Fadama III programme was a successor to Fadama II, the Nigerian government aimed to expand the programme across the whole country, rather than in select regions. This expansion of resources was not adequately covered by the programme budget, leading to low funding in certain areas, with Fadama II beneficiaries feeling particularly affected (Hima et al., 2016).

For both BS and GNAEP, there was evidence that plans were never implemented by the intervention team, either as the proposed aquaculture programme was deemed unsuitable for the local ecological systems, or because the initial planning was inadequate or absent.

A decrease in the participant's motivation was a barrier specific to FoF, the only RCT we identified in the review. The randomisation of women to one of the two interventions arms within the programme led to a decrease in motivation, as participants felt uninvolved in the process. This hindered the programme from set up and meant that women farmers were not as motivated to achieve the programmes' objectives as perhaps they could have been. This finding had further implications, as the intervention team decided to change this feature in the follow‐up phase of the programme (Green et al., 2018).

Lastly, unclear roles of the intervention partners was identified in GNAEP, addressing specifically how the programme was set up by the implementers. DANIDA (2008, Annex 4) identified that the main implementers in the programme were not able to take on the work that had been envisaged at the project's proposal stage, leading to implementation issues resolved as the project progressed.

#### 5.4.3 Factors affecting participation

Whereas each theme for programme set‐up was identified as a barrier, factors affecting the participation of beneficiaries were seen as both barriers and facilitators. These findings are presented in Table 5.

**Table 5:** Summary of findings for factors affecting participation

Theme and programmes reporting this finding	Evidence example	Papers contributing to this finding
**Social/cultural** BS CBFC ESP Fadama III FoF MAEP	“The female credit farmers in Gafargaon have minimal decision‐making power. It is entirely up to men whether they will consult their wives before making any household decision. In general, men take all the decisions regarding the household budget. The women also do not have control over their own bodies; husbands decide how many children a woman must bear. While a few men now discuss with their wives how many children they will have, men always make the final decision. Similarly, the timing of childbearing is also not under women's control. Both the decision to use contraception and the choice of method depend on men. Men never use male methods. Thus, female credit farmers do not notice any change in their status within the household that they could attribute to fish cultivation” (Naved, 2000, pp.46–47).	DANIDA (2008), Annex 4; Fadare and Adereti (2017); Hallman et al. (2003); Kioi (2014); Kumar and Quisumbing (2011); Meizen‐Dick et al. (2003); Moumin (2016); Naved (2000); Omobowale and Akinola (2017); Rand and Tarp (2009); Sheriff et al. (2010)
**Income** AFP BS CBFC GNAEP FoF MAEP	"Second, attrition was higher than expected during the project, perhaps in part because of better livelihood opportunities for women in other geographical areas that resulted in an employment‐related temporary relocation” (Michaux et al., 2019, p. 10)	FAO (2009); Hallman et al. (2003); Michaux et al. (2019); Meizen‐Dick et al. (2003); Moumin (2016); Naved (2000); Pant et al. (2014); Sheriff et al. (2010); WorldFish (2009)
**Programme delivery** BS CBFC DSAP ESP Fadama II Fadama III FoF GNAEP IAA MAEP	“Farmers are demanding more effective support from the FAs to increase their fish production. The MTR team observation corroborates the concern of the farmers for more quality training. This indicated a need for improvement in training approaches and quality of the trainers. Many of the NGO staff lack adequate capacity in exercising participatory practices. It happened due to frequent dropout of the FAs, although some NGOs have made efforts to cover the DFGs support with the help of other staff including project coordinator” (Mandal et al., 2004, p. 21)	DANIDA (2008), Annex 4; Dey et al., 2010; Fadare and Adereti (2017); FAO (2009); Hallman et al. (2003); Hima et al. (2016); Kiwiri and Njeru (2015); Mandal et al., 2004; Meizen‐Dick et al. (2003); Moumin (2016); Njagi et al. (2013); Omobowale and Akinola (2017); Ovharhe (2020); Sheriff et al. (2010)
**Access to natural capital** BS FoF IAA MAEP	“Farmers who have access to extension services are more likely to adopt IAA, ceteris paribus. Also, the likelihood to adopt IAA is higher for older farmers with larger farm area and a greater number of enterprises. At the same time, access to irrigation enables a higher intensity of adoption. The available land area is a significant explanatory variable, having a positive effect on IAA adoption and the level of integration” (Dey et al., 2010, p. 23)	Dey et al. (2010); Hallman et al. (2003); Meizen‐Dick et al. (2003); Moumin (2016)

Social and cultural factors were identified for six programmes, covering topics such as gender norms and trust in government‐run programmes. Gender norms were identified as a barrier for the MAEP, BS and FoF programmes. Naved (2000), presented a thorough qualitative analysis of gender norms in the MAEP and BS intervention areas of Bangladesh. The author also identified barriers that could have affected the level and way of involvement of participants of these programmes, and thus, the results that were expected for these interventions. In MAEP sites, women's lack of knowledge of aquaculture production and of decision‐making power in aquaculture activities was mentioned; thus, their role was commonly limited to fish feeding and other restricted activities. Gender discrimination in food allocation was also highlighted in MAEP and BS sites by the author, and while the programmes were not able (or expected) to change this, an apparent increase in the fish consumption at the household level also seemed to increase the consumption of fish for women. In turn, for some women participants of FoF in Cambodia, the programme was identified as a burden. Traditional gender roles meant that women were usually preoccupied with cooking and cleaning tasks; then, with the additional duties that the aquaculture intervention entailed, women needed to ask for help from other family members; thus, they were less able to get involved in the aquaculture process. A possible implication of these gender roles is that we may not be seeing the full potential of the programmes in terms of their expected outcomes on nutrition and women's empowerment, although it is difficult to measure exactly how much these cultural barriers could be affecting the programmes' effectiveness.

Moreover, in MAEP, BS, and Fadama III, group conflicts were identified as a barrier affecting the participation of beneficiaries and the implementation of these programmes. Related issues mentioned ranged from intragroup disagreements and lack of cooperation, to the deliberate poisoning of ponds. These conflicts made some farmers stop participating in the programmes, or hindered the implementation of the interventions, particularly as these programmes were based on group decision‐making and collaboration.

Although this was identified in one programme, social acceptability of aspects related to fish was also identified as a potential barrier to participating in aquaculture interventions. In certain areas of Kenya where the ESP programme operated, a quarter of respondents believed that fish farming as an occupation was not an accepted activity, and only 30% believed that eating fish was socially acceptable (Kioi, 2014, p. 38). Again, these figures only represent one area in which a subnational programme was implemented, speaking to the lack of representative evidence we were able to locate.

Low trust in government was identified as a barrier to participation in four programmes MAEP, BS, Fadama III and CBFC. Evidence pointed out to a low level of confidence in public agencies and officials, as well as a general perception of corruption towards local and national governments. This hindered the participation of interested individuals with the fear that these programmes would be “white elephant projects” destined to fail (Omobowale & Akinola, 2017). The involvement of official agencies in these programmes was a concern especially in cases where the land where the intervention took place was privately owned. Sheriff et al. (2010) reported that, for CBFC, extra measures around transparency had to be taken to manage the mistrust of beneficiaries.

The theme of income was also mentioned in a number of programmes as a factor that both helped and hindered the participation of farmers. MAEP, GNAEP, BS, AFP and FoF project beneficiaries deemed that the returns from aquaculture activities were many times insufficient, which prevented them from practising aquaculture as their only or main means of livelihood. Similarly, AFP, FoF and CBFC records identified seasonal migration as a major issue given that beneficiaries migrated to find greater economic opportunities, and thus, stopped participating in these aquaculture interventions. While project beneficiaries may also decide to diversify their means of livelihood to reduce a perceived risk associated with aquaculture activities, this could still suggest that aquaculture may not be perceived as a stable and sufficient source of income. Despite these barriers, the income theme was also seen as a facilitator. The ability to save some extra income as a result of programme activities was identified in MAEP, BS and FoF as a positive aspect of participating in these programmes. MAEP was designed in a way that income from aquaculture activities was not immediately available to use or spend, and was instead put in savings accounts for the duration of the programme. Beneficiaries reported preferring this arrangement as it would help them invest in future plans (Naved, 2000, p. 33). There is some evidence from the BS programme that the increased income from aquaculture activities would be more equally distributed, both at the group and household levels.

Programme delivery was identified as one of the most important themes, as it was mentioned for 10 of the 13 included programmes. The first aspect of programme delivery, reported in three programmes, was the identification of bad practices in the implementation of the interventions as barriers to participation. GNAEP recorded some petty misuse of resources, while reports on Fadama II and Fadama III found that at least in certain states there were established issues of local corruption.

The second aspect identified within programme delivery was project support. This was one of the fundamental factors affecting participation as this support can define whether an individual chooses to partake in a programme. Reports for MAEP identified that some of the training programmes were overly simple, and ESP beneficiaries felt they were not supported and visited by extension officers often enough. Similarly, the DSAP project was scaled back and extension officer visits decreased, which negatively affected the motivation of participants. Conversely, in FoF, IAA, CBFC, Fadama II and Fadama III, programme participants were increasingly motivated by the constant support and supervision provided by the projects. Furthermore, MAEP, GNAEP and BS participants were motivated to participate in the programmes due to the access these provided to training and inputs. Overall, programmes that provided constant support and sufficient visits by extension officers were highly valued and the access to training was seen as a motivating factor for participants.

The final theme in this dimension is access to natural capital, which was identified as a barrier or facilitator depending on the focus of the programmes when targeting beneficiaries. A lack of access to natural capital can be a barrier to the adoption of aquaculture programmes, but programmes can be designed to overcome this barrier. For example, MAEP and BS participants highlighted the fact that none of these programmes required the ownership of land, which increased the likelihood that landless farmers could partake in the intervention. In contrast, IAA found that farmers with access to larger areas of land were more likely to adopt the new technology recommended by the programme. Additionally, FoF reported that the lack of access to water year‐round prevented women farmers from fully participating in the programme.

#### 5.4.4 Factors affecting productive activities

We present in Table 6 the findings that emerged around factors affecting the productive activities along the aquaculture value chain. These could mostly relate to the effectiveness of the programmes when production is an outcome of relevance.

**Table 6:** Summary of findings for factors affecting productive activities

Theme and programmes reporting this finding	Evidence example	Papers contributing to this finding
**Access to funding** BS Fadama II Fadama III FoF GNAEP MAEP	“However, there were constraints. It became clear that the package was not suitable in all contexts of Noakhali (there are probably 5 or 6 different ecological systems), especially in the charlands with their limited water holding capacity and in the areas close to the Indian border. Moreover and more seriously, the package did not address the problems of the poorest groups, especially since the concentration of the NGOs upon credit realisation meant that they tended to recruit the more creditworthy into the system. Finally, the net returns from the improved pond polyculture offered only a limited improvement to livelihood even for those with better pond resources” (FAO, 2009, p. 50)	Alawode and Oluwatayo (2019); Bature et al. (2013); DANIDA (2008), Annex 4; FAO (2009); Hallman et al. (2003); Meizen‐Dick et al. (2003); Moumin (2016); Olaoye et al. (2011); Omobowale and Akinola (2017); Ovharhe (2020)
**Access to inputs** AFP DSAP ESP Fadama II Fadama III FoF	“It is important to note that the sustainability of the livelihoods of resource‐poor Adivasi households depends not only on the continued viability of income‐generating activities, but also on continued access to aquatic resources. This was evident for the single community‐based fisheries group established under the project, which was revisited in 2012 and was found to be continuing with the management of community aquatic resources for production of culturally significant living aquatic resources (especially crabs, snails and swamp eel) for subsistence consumption. However, it proved difficult to secure access to floodplains on behalf of other Adivasi communities while the project was active. Scaling up interventions of this type thus may be problematic” (Pant et al., 2014, p. 9)	Mandal et al. (2004); Moumin (2016); Njagi et al. (2013); Pant et al. (2014); Olaoye et al. (2011); Ovharhe (2020)
**Economy** BS Fadama II FoF MAEP SAFAL SMART‐Fish	“For respondents that mentioned generating surplus vegetable, fruit, and fish products for sale, follow up questions regarding market challenges and transport of products were asked. The most common responses among positive and negative deviants alike was selling products at the village market and directly at the farm gate to neighbours. When asked about pricing of products, respondents resoundingly expressed their anger towards middlemen with all but one respondent reported losing profits by selling to them. In addition to negotiations with middlemen, respondents also stated that competition from other sellers was a major challenge in the sale of their EHFP outputs. In fact, almost all respondents mentioned that there were many other vendors who sold similar products and at the same price. As a result, in order to generate income, respondents mentioned they would often have to sell at a similar or lower price. When asked about how prices were determined, it was unanimously reported that prices were set commonly amongst all vendors; deviating from which would be met with negative criticism and loss of customers” (Moumin, 2016, p. 25)	Hallman et al. (2003) Kessler et al. (2017); Meizen‐Dick et al. (2003); Moumin (2016); Olaoye et al. (2011); Rand and Tarp (2009); UNIDO (2019)
**Environmental shocks** AFP DSAP FoF GNAEP SAFAL	“In 2008, due to the flash flood, Naltitabari (32 ponds and 18 rice fields), Durgapur, Kalmakanda Upazilla of Sherpur and Netrokona district respectively were severely affected. It was estimated by Caritas that the loss of fish and vegetables in the pond dike of AFP farmers were around Tk. 139, 096. Flood losses also occurred in Khalchanda village where a mud built embankment near a big depression was totally damaged. The 36 members of the Kuch community who reside in the village depend on this resource for fish consumption. The irrigation water for the rice fields also comes from this depression. Due to damage of the embankments 6 rice‐fish and 9 cage farmers of AFP were not able to do fish culture in 2008” (WorldFish, 2009, p. 12)	DANIDA (2008), Annex 4; Kessler et al. (2017); Khondker and Pemsl (2011); Michaux et al. (2019); Moumin (2016); Pant et al. (2014); WorldFish (2009)
**Infrastructure** Fadama II GNAEP MAEP SAFAL SMART‐Fish	“Poor communication, specifically roads, and the high cost of land and sea transport is a factor that hampers development in the seaweed and pangasius value chains. There are many areas well suited for seaweed production in Indonesia, but transport to processing centres is too expensive. In the pangasius value chain, poor transport is also a factor that hampers pangasius production in many areas. Long transport time also results in higher fish mortality on the road. SMART‐Fish has developed approaches to mitigate this problem but depending on the distance of transport losses can remain high” (UNIDO, 2019, p. 51)	DANIDA (2008), Annex 4; Kessler et al. (2017); Olaoye et al. (2011); UNIDO (2019)

Different experiences of accessing funding and credit could partly explain why beneficiaries were able, or not, to take loans to increase inputs and in turn yields. Three programmes, MAEP, GNEP and BS identified that access to funding was dependent on connections. Hallman et al. (2003) identified that poor women in the BS programme felt that credit was only given to those with good networks to implementing partners, a connection they were excluded from. In GNAEP, Fadama II, and Fadama III the access to funds was deemed as insufficient. This is not a surprise for Fadama III, as we know from the programme set up dimension that the intervention's budget was not sufficient for the third upscale. However, GNAEP respondents claimed that the two types of credit models available through the programme were too standardised, and that in practice, these did not work for the groups they were intended to help. Participants in FoF and Fadama III also mentioned a fear of taking out loans, as they worried about being unable to pay the costs back. Despite this, there were also some positive notes around the access to funding in these programmes. Fadama III participants (from a different region from those characterised earlier) stated that accessing funds was key for them to be able to get involved in aquaculture activities, a feature that also resonated with MAEP beneficiaries.

One way the included programmes could have overcome the issue of funding is by providing inputs directly to beneficiaries. Despite this, if a programme simply provides training with no inputs or provides inappropriate inputs, this could become a barrier for the success of these interventions. Six programmes, AFP, FoF, ESP, DSAP, Fadama II and Fadama III mentioned, to varying degrees, that a lack of inputs was identified as a basic barrier to productive activities. In the AFP programme, there were issues securing floodplain access on behalf of the marginalised Adivasi people, meaning that the continuance or expansion of this programme was at risk. FoF beneficiaries cited that, in addition to the regular inputs they would receive as part of the intervention, fertilisers and pesticides were required to increase the yield, yet these were not easily accessible or provided. In turn, DSAP also reported a lack of quality fingerlings in programme areas.

Another theme that emerged relates to the potential effect that the general economy could have on the productive activities along the aquaculture value chain, both in positive and negative ways. Market instability was identified in three instances as a possible barrier to the effectiveness of our programmes. For Fadama II, there is some evidence that high inflation rates of the economy was deemed as a very serious problem affecting aquaculture production. For MAEP and SMART‐Fish we were able to identify further details. Rand and Tarp (2009) argued that a decrease in the prices for fish from 1996 to 2006 limited the long‐term success of MAEP, while UNIDO (2019) identified that the Indonesian government's seaweed export policies had a potential hampering effect on the price of this product. Whereas government policies could have affected negatively to SMART‐Fish beneficiaries, MAEP and BS participants seemed to have benefited from the Bangladeshi government's trade liberalisation, which led to a general increase in the availability of aquaculture inputs. Additionally, both MAEP and BS highlighted the creation of additional jobs as a result of the use of technologies provided by these programmes. When looking at this issue at a local level, we identified some evidence of local market challenges. For FoF, respondents reported losing profits due to having to sell their products through markets' middlemen, whereas in SMART‐Fish while the quality of fish was one of the main outcomes of the programme, local markets did not always reward this quality. This meant that participant farmers found it difficult to compete with feed supply companies. In turn, Kessler et al. (2017) were careful in completely attributing their results to the SAFAL programme, stating that the general economic growth of Bangladesh may have had also an effect on income outcomes.

Environmental shocks was an additional finding that could prevent the effects of the programmes from being fully realised. A number of programmes, namely DSAP, GNAEP, AFP and SAFAL, reported that floods severely interrupted the development of aquaculture productive activities. For example, in AFP, one site was stopped altogether as a result of floods, while in SAFAL, respondents reported losses of up to 50% of yields due to flooding. In addition, FoF farmers faced major issues with droughts, with about 30% of fishponds drying up, which together with poor soil conditions prevented them from growing their aquaculture production.

Lastly, and similar to environmental shocks, infrastructure was an identified factor outside of the control of the programme implementers. Fadama II and SMART‐Fish identified deficiencies in infrastructure as a barrier to the progress of aquaculture activities. For example, the SMART‐Fish programme reported that many parts of Indonesia were suitable for aquaculture production but that poor communication systems and roads, paired with the high cost of sea and land transportation, were key issues for farmers. In practice, this meant that for these areas the development of aquaculture productive activities was almost inaccessible. In contrast, for MAEP, GNAEP and SAFAL, general improvements in the national infrastructure and communication systems were identified around the time these programmes were implemented. This facilitated the growth of aquaculture production and the access to local markets, as farmers were able to transport their products faster.

### 5.5 Summary of findings from cost evidence

In the following section, we present a synthesis of the evidence to address review question 5 around the cost‐effectiveness of aquaculture interventions. We include a description of the general findings of our cost analysis, followed by a brief summary for each of the programmes for which we were to identify relevant data. We found 10 sources reporting either a budget or expenditures, associated with nine of our included aquaculture programmes. Some reported a unit cost or number of households affected, while a few reported benefit‐cost ratios using unit budget/expenditure and average household gains in terms of net income. However, the overall quality of the cost analyses was generally poor. A summary table of the sources and analysis for this section is provided in Appendix D.

#### 5.5.1 Extent and quality of the cost evidence

The aquaculture programmes included in the review varied in terms of what activities were carried out. Although all sought to increase income from aquaculture activities, one project was intended to affect policy rather than implement an intervention that directly affected the income of the poor (Fadama III). In two cases we were able to identify two reports for a single programme. For one of these interventions (SAFAL), accounts differed in the two reports as to what the project was, although both reported the same budget.

We only found cost per household for programmes in Bangladesh, where six of the nine programmes with cost data were located (AFP, CBFC, DSAP, GNAEP, MAEP and SAFAL). These interventions seemed to have two components—construction of enclosure of fish culture and stocking of fingerlings and fish culture– both of which could be costed by examining the inputs that went into it; however, none of the sources reported this. The first component could be thought of as fixed or investment cost, while the second may involve assistance in smoothing out the routine supply chains (Kuijpers, 2020). For a small project in Bangladesh (CBFC), the authors provided unit fixed and variable costs without further explanation as to how these figures were calculated. The other projects in Bangladesh provided a budget and number of households reached, along with dividing the budget over the number of households reached. The yearly cost per households for the programmes never exceeded $300, and the benefits never exceeded $900 measured in 2019 USD; these upper bounds were identified for the same programme that lasted 4 years (SAFAL). The lowest cost for reaching a household was $19 per annum, determined through the budget allocation (MAEP).

The two programmes from African nations were part of larger agricultural projects. Effectiveness was described in detail for Fadama III, while no unit cost could be obtained for ESP as the report did not detail how many households were affected. World Bank's (2016) large project in Nigeria (Fadama III), in which aquaculture was a small part, generated an encouraging benefit cost ratio of 1.51 for the aquaculture component; however, there was no account as to how costs were calculated.

From the reports used for this analysis, none of the interventions that worked with farmers seemed to have clear explanations as to what the programme was and the elements for which money was spent; there were generally no annex or documents referenced for this type of information. Therefore, it is only possible to obtain an understanding of the programmes and their costs in broad generality. Below we describe the main aspects to consider from each of the programmes assessed.

#### 5.5.2 Main features of cost data for included programmes

While some of the SMART‐Fish intervention components were intended to have broad impacts, one of the impacts considered was the generation of more money fitting into some of the components the programme funded: $11 million were generated for an improved educational programme in fishery and finding methods for research on productivity. Evaluated at 2019 USD, SMART‐Fish budgeted for six components at a total of $2.2 million with $1.56 million for admin support. A total of $3.26 million were spent on project operations, support costs, round tables, quality and productivity centre, traceability system, trade promotion and evaluation. Analyses of the report (UNIDO, 2019) could not account for USD 0.47 million.

The MAEP and GNAEP programmes had a specific intent that is amenable to costing and impact evaluation. The costs reported (DANIDA, 2008, Annex 4) are expenditure undertaken at the end of programme; however, as there is no information on how these were collected and for what purposes, we cannot say we know the cost of replicating the intervention elsewhere. There are no proper cost‐effectiveness analyses to report. There are different types of aquaculture approaches tried and different types of beneficiaries. Using the budget and the number of reported households affected we can report the following in annual costs in 2019 USD: $19.43 for MAEP, and $24.40 for GNAEP.

The AFP programme, sponsored by the European Union, was a small project affecting 3600 households. This would have been amenable to costing the programme by input usage and linking it to output. However, this was not done. One of the studies covering AFP (Pant et al., 2014) reports the programme cost spent on asset development for household, so we could link this cost to one of the outputs. Although households were given different support, total per‐capita devoted to asset development was about $50 measured in 2019 USD value. Expenditure is detailed for 2008 in another study (WorldFish, 2009); however, the values are not amortised, for example, for computers. Little consideration is given to the type of input shadow prices, prioritising just spent amount. Costs are not linked to output, which can be difficult for aquaculture programmes. The reporting of expenditure is a model for reporting costs for inputs used, and probably meets accountability needs. It does not, however, report economic costs. Total cost of the project in 2008 was €296,227, at an average cost valued in 2019 USD of $138.

The CBFC programme had a small scale, with only 778 households as the beneficiaries. The size of the project would be perfectly amenable to carrying out a cost‐effectiveness analysis. The costs are divided into fixed and variable costs, and the project does seem to have a distinct investment and an on‐going component. It is possible that this was derived from budget allocation to each of these items. The cost per household was $206, with fixed cost at $153 and $51 for variable costs; and we were able to estimate the benefit cost ratio as 1.85, however, this value differs from what is reported in the impact evaluation paper (Haque & Dey, 2017).

The SAFAL intervention was a large project which may have had considerable fixed costs. As the evaluation (Kessler et al., 2017) does not break down the programme components, it is hard to know what the costs were. For example, the budget was used as cost, which may not be entirely accurate in practice. Kuijpers (2020) rephrased the programmes as a supply chain project which aimed to reduce transaction costs that farmers face; it would have been interesting to have more details on the main components of the project and how much they cost. Moreover, the number regarding households reached are different in these two studies. It is possible that lower effectiveness is due to the more rigorous study in Kuijpers (2020). The benefit cost ratios are programme ratios, earned over September 2012 to August 2016, and were calculated as 1.5 from Kuijpers (2020) and 10.7 from Kessler et al. (2017).

DSAP was a 5‐year project funded by USAID, with a budgeted amount to 5.5 million, or approximated at $7.66 in terms of 2019 USD valued at the midpoint of the programme. This was a mainly aquaculture‐focused project spending $200 per household in Bangladesh to reach 35,000 households in their demonstration lists. We were not able to identify or calculate a cost‐effectiveness ratio or benefit cost ratio from this study (Mandal et al., 2004).

There is little information around ESP. From the impact evaluation paper (Amankwah et al., 2018) we know that the programme received $17.87 million in 2009; however, we were not able to ascertain unit costs associated with the intervention.

FADAMA III is an ongoing programme not yet fully evaluated. It was difficult to determine from the report (World Bank, 2016) when any of the budget was spent. Assuming that the average time at which the $290 million were spent is 2012, in terms of USD 2019, $316 million had been spent. The main beneficiaries were accounted as 965,000 households. The analyses offer benefit cost ratio for several types of agricultural activities over 15 years, for activities ending in 2013; however, programme spending does not occur for 4 years of that period. With the assumption that only FADAMA III affected the programmes sites, over a period of 11 years, $350 was spent per household. For aquaculture activities, the authors reported a benefit cost ratio of 1.51, although it is not clear how costs were calculated. Finally, we were not able to identify cost data for the following programmes: BS, Fadama II, FoF and IAA.

## 6 DISCUSSION

### 6.1 Summary of main results

Our systematic review explored and analysed the existing evidence on the impact of aquaculture interventions in low‐ and middle‐income countries on four specific dimensions: productivity, income, nutrition, and women's empowerment. The quantitative analysis showed a small and significant impact of aquaculture programmes on some important production, income and nutrition measures. In particular, production value, income, total expenditures, and food consumption all showed a significant increase as a result of the aquaculture interventions. However, these findings show substantial variability, which we were not able to try to explain as the small number of studies included in the review prevented us from conducting moderator analyses.

We found a small significant impact of aquaculture interventions on production value. This finding would represent an average increase of around $53,^12^ measured in 2021 USD, in the yearly production value of participating farmers. We did not find a significant effect on production volume. Following Kuijpers (2020), one potential explanation for these findings could be that the increased value of the production is driven by higher prices rather than by a larger production quantity. However, the data available from our studies did not allow us to test this theory further.

Results also showed a small but significant positive impact of aquaculture programmes on an aggregate livelihood measure, as well as on individual measures of income and total expenditures. We found no impact on food expenditure, household assets, farm profits, and poverty levels. When looking specifically at income measures, the analysis showed that aquaculture interventions have a positive significant impact on the income of participants, which would be translated to an increase in a household's yearly income of $67 measured in 2021 USD. In turn, the analysis on total expenditures at the household or farm level also showed a positive and significant effect, which would represent an average increase of $26 in purchases, measured in 2021 USD.

While only two studies looked at food expenditure, the analysis suggested that this effect is statistically not different from zero. However, we found a positive and significant effect on fish consumption, which would correspond to approximately an additional 200 grams in the household's monthly fish consumption. The results around food expenditure and fish consumption could be driven partly by the limited number of studies available, and while these two studies did not provide further details on how these measures were calculated, this finding could also suggest that farmers might consume more own‐produced fish but are not necessarily spending more money on other types of food.

We were able to identify very limited evidence on intermediate measures of nutrition, such as food security and quality of diets. Therefore, we were not able to assess and synthesise the impact of aquaculture programmes on these outcomes. However, we identified a few studies reporting on anthropometric measures, including women's and men's BMI, and 0–5 year old children's height‐for‐age. In line with the aquaculture and nutrition literature, we did not find a significant impact of aquaculture programmes on these anthropometric measures.

Likewise, we found scarce data on the effect of aquaculture interventions on women's empowerment. These few studies collected measures with little comparability and, due to the way they were reported, we could not synthesise these data quantitatively. Although the evidence showed mixed‐results, contextual and implementation aspects of these programmes, such as gender norms and individual‐ versus group‐based interventions, were mentioned to explain the presence or absence of effects on women's empowerment measures.

When analysing barriers and facilitating factors related to the aquaculture interventions, three dimensions emerged: factors affecting the programme set up, the participation of beneficiaries, and the level of productive activities. On programme set up, only barriers were identified, which related to low funding, participants not being able to choose the intervention package, unclear roles of partners, and project plans that were never implemented. On participation, the main barriers and facilitators were social and cultural norms, income from aquaculture activities, programme delivery aspects, and access to natural capital. On productive activities, we also identified positive and negative factors, including access to inputs and funding, general economy, infrastructure and environmental issues.

Related to the cost‐effectiveness of aquaculture interventions, the data available did not allow us to make full comparisons across programmes. However, the yearly cost per households for programmes in Bangladesh never exceeded $300 and the benefits never exceeded $900, both measured in 2019 USD. The lowest cost for reaching a household was $19 per annum in 2019 USD.

### 6.2 Overall completeness and applicability of evidence

As part of the screening at the full‐text stage, we found that a high number of potentially relevant studies were excluded because they used an inappropriate study design, and from this group, a high number of studies were also based in Bangladesh. Papers excluded by study design at full‐text (*n* = 70) covered 27 low‐ and middle‐income countries, meaning that besides the six countries already covered by included studies, papers excluded by study design focused on 21 additional countries. Among countries covered by these excluded papers, Bangladesh was the focus of a quarter of these papers, followed by Nigeria. It is clear that there is a large number of aquaculture studies that take place in Bangladesh, and so it is no surprise this makes up more than half of our included papers (12 of 21 studies focused on Bangladesh). In addition, within papers excluded by study design, 13 focused on our included programmes, and we also identified 44 additional interventions studied by this set of excluded papers. Thus, we find that there is a vast amount of literature on relevant aquaculture programmes across a variety of countries; however, in too many cases they do not use appropriate methods to identify an effect attributable to the aquaculture intervention. In addition, Bangladesh seems to be a focal point for aquaculture programmes and research around those interventions, although these are not always impact evaluations. Thus, the results of this review may be more applicable to Bangladesh than to the rest of the countries covered by included studies.

In light of the majority of extant literature being focused on Bangladesh, we looked at the extent to which external validity issues were reported or discussed in included studies. We found that issues around generalisability were not discussed at all in more than half of these papers (14 of 22 papers with risk of bias assessment). External validity was directly or indirectly addressed in the rest of the included papers. Four papers addressed this directly: the two papers covering SAFAL, and two of the three studies covering FoF. In these cases, the authors addressed the limitations of their results and made it clear that implementing those programmes in a different context would not necessarily provide the same results. In the other papers, external validity was addressed in a more general way when discussing the results, by stating, for example, that the programme had an effect “on the study areas” but without explicitly acknowledging or discussing that the findings are specific to the context.

Related to the external validity of our systematic review with regard to its interventions of interest, we can mention two points. First, is the fact that, within our 13 included programmes, only one is a RCT. This limits the generalisability of the results found that are particular to this programme (e.g., the barrier of programme set up related to the randomisation of the intervention packages), and also the results of this review in the general context of aquaculture interventions in low‐ and middle‐income countries. Second, the majority of the included programmes focus on the preproduction or production stages of the aquaculture value chain. Thus, the results presented in this review would be less applicable to stages after production, such as processing, marketing, or trading.

While we have been able to investigate in some way all main outcome groups defined for the review, namely productivity, income, nutrition and women's empowerment, we could not synthesise evidence for many individual outcomes. The most important of these cases relate to measures of women's empowerment, which was based on three programmes and allowed only a descriptive analysis. Other outcomes that were reported in only one programme include production quality, farm revenue, market participation, received prices, micronutrients intake and blood concentration measures.

In turn, because we did not impose restrictions on the participants of interest for this review, we were able to cover all groups for which data was reported. However, only a few studies presented results for subgroups. For example, only one of the included studies (Quisumbing & Kumar, 2011), reporting on two programmes, presented results using sex‐disaggregated data. This paucity of data at the subgroup level also connects to the question on the relevance of our body of evidence to address the questions defined for the review. Even with the caveats discussed in these sections, we were able to respond to review questions 1, 4 and 5, namely about the overall effectiveness of aquaculture interventions on our outcomes of interest, about the barriers and facilitators that affect these interventions, and the cost‐effectiveness of these programmes. However, the lack of suitable data prevented us from addressing questions 2 and 3, related to spillover effects and differential effects by subgroups. Our intention is to revisit these questions when we update this review.

### 6.3 Quality of the evidence

A total of 21 studies were included in this review, of which we used 19 in our synthesis. These studies covered 13 different aquaculture interventions in low‐income and lower‐middle‐income countries. We found no evidence for programmes in middle‐income countries. For the quantitative synthesis using RVE, 12 programmes contributed a total of 53 effects in the analysis of a livelihood measure; in turn, when meta‐analysing the effect of aquaculture interventions using specific outcomes, we were mainly able to use measures covered by between two to five programmes per outcomes, and only in one case we could analyse data representing 10 of the 13 included interventions. Thus, in many cases, these analyses were not able to fully represent the range of aquaculture interventions identified in the review.

Among our included studies, two papers have an overall low risk of bias, eight papers have some concerns related to its risk of bias, and 12 papers were assessed with an overall high risk of bias.^13^ The most common methodological limitations in included studies, as assessed by their risk of bias, were related to confounding bias, reporting bias, and spillovers, cross‐overs and contamination bias. All of the studies assessed as not free or probably not free of these biases were QEDs, which were also the majority of the studies. The critical appraisal of included studies suggests that the overall quality of this evidence is low, particularly for the QEDs.

As part of the risk of bias assessment of included studies, we also coded if studies reported having ethical approval. Ethical clearance was reported for the only RCT study but not for any of the studies based on QEDs. In the case of the RCT, ethical approval was reported in two of the three papers that cover the same programme (Michaux et al., 2019; Verbowski et al., 2018). Though the third paper for the RCT (Talukder & Green, 2014) does not mention having ethical clearance, we could assume this is more likely a reporting issue rather than a lack of such clearance. In turn, many of the QEDs are retrospective and did not report having obtained ethical clearance. Though this does not justify the absence of information regarding ethical approval, the retrospective nature of the studies may be the reason why there is a lack of information. Ethical clearance is especially important when programmes have a focus on sensitive topics, such as gender inequality or empowerment. The fact that the only RCT included in the review, which targeted women, reported having ethical clearance is encouraging.

By using different documents to inform our analyses, we were able to identify some issues related to the consistency of evidence around our included aquaculture programmes. Particularly, we found that some of the themes that emerged in the barriers and facilitators analysis had different levels of correspondence with our quantitative synthesis results. In the first instance, few of the themes identified in qualitative data had a direct correspondence to our meta‐analysis results. For example, we found that interventions in countries with a “very high” exposure to climate shocks index had a significantly lower impact on income. In turn, environmental shocks, such as droughts or floods, emerged as a barrier to participating in these programmes, which would ultimately affect their ability to generate a higher income from aquaculture activities. While this evidence provides more confidence in the result, this correspondence is not very common.

Second, we found that for some of the themes that emerged from the qualitative analysis there is no quantitative synthesis to compare them with. Because this and other similar information was not usually reported in the impact evaluation studies, we were not able to code it and incorporate it in the meta‐analysis. Examples of this include the themes on access to land (this information was coded as unclear for the majority of the studies), and project support (this information was not usually reported in such detail). Third, some qualitative themes may seem inconsistent with our quantitative synthesis results. For example, while income emerged as both a barrier and a facilitator of participation, its evidence as a barrier—highlighting that returns from aquaculture are insufficient—does not correlate with our meta‐analysis results, which showed a significant and positive impact on income. This may be explained by the different expectations, perceived risks, competing opportunities, and general expenses for beneficiary households. The quantitative results showed an increase of $67 USD per year, which though statistically significant, may not be enough or may just not be better than other opportunities for these households.

**Figure 18:**
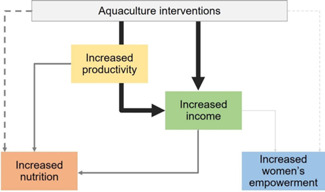
State of the evidence around aquaculture interventions

Figure 18 summarises the state of rigorous evidence around aquaculture interventions following the theory of change defined for the review. The productivity and income pathways are the ones where we identified more comparable evidence, as denoted by the thickness of the arrows. Between 1 and 10 programmes reported outcomes within these groups, and we were able to synthesise quantitatively the majority of these measures (66% of the outcomes in both pathways). For the nutrition pathway, we found less rigorous evidence, as denoted by narrower and lighter‐coloured arrows. Up to six programmes reported these types of outcomes, and we were able to synthesise 57% of these individual outcomes. The more limited evidence on the nutrition pathway may reflect the relatively recent interest in rigorously evaluating the linkage between aquaculture—and agriculture more generally—and nutrition. For example, a recent update of a review looking at this linkage found more evidence with consistent impacts of agriculture programmes on nutrition outcomes (Ruel et al., 2018). Finally, the pathway for women's empowerment is the one where this review is less able to inform, as denoted by even lighter arrows. Up to three programmes reported a range of different indicators of women's empowerment, but because of the diversity and the little comparability of these measures, we were only able to present this pathway descriptively. The review covered an extensive period of time (i.e., from 1980 onwards), so these results reflect that the evidence around aquaculture and women's empowerment is relatively new. The studies for the three programmes reporting these outcomes could be taken as state‐of‐the‐art research on this specific area, and as such, it may be less surprising that their data is less comparable.

### 6.4 Potential biases in the review process

One potential bias we could have from the review process is related to the lack of complete data in included studies. In many cases, the reports did not include all the necessary data to directly translate the results into effect sizes. We tried contacting authors to access the full data for the four most serious cases but were unsuccessful in three of them. Thus, we had to make assumptions in the extraction of effect sizes for two‐thirds of included studies (15 of 22 papers with risk of bias assessment). An important implication of this was that we had little variation among effect sizes, which was especially problematic for studies reporting many effects as we were not able to account for the full range of results from these studies. Moreover, we also had to make assumptions for a quarter of the studies (6 of 22 studies) related to the sample size used, as this was not explicit in the reports. While we made a conscious effort to apply these assumptions consistently, this may be a source of bias in our results.

Related to the quantitative synthesis of results, we did not conduct Egger's tests for outcomes with <10 effect sizes. In practice, this meant that we were only able to assess the presence of publication bias for one outcome, namely, income of beneficiaries. While we took active measures to minimise this potential bias, including searching an extensive list of grey literature sources and the inclusion of studies regardless of their publication status, we could not rule out the possibility that the results presented in the review could still suffer from publication bias.

An additional potential bias in our review is related to the search strategy and the possibility that we may have missed some relevant studies. Particularly, the fact that we did not include sources specific to thesis, dissertations and conference proceedings in the list of grey literature may have affected the likelihood that all relevant unpublished studies were identified. In the same line, we did not include the term “prawn” in the full search strategy, which may have potentially left out relevant studies. However, because we tested the strategy to achieve a balance between comprehensiveness and feasibility, and we based the search on key terms and indexing, we deem that the review worked with a solid search strategy. In addition, we published an institutional blog calling for further evidence, from which we were able to identify one additional included study. Nevertheless, we will try to improve the search strategy when we update the review.

### 6.5 Agreements and disagreements with other studies or reviews

Our review represents a novel contribution to the literature on aquaculture. To our knowledge, no previous systematic review has been conducted with a specific focus on aquaculture interventions and that represents in itself an important contribution towards building a useful evidence base to inform future programming and research. The closest evidence we could access to inform our thinking and expectations on the impact of aquaculture programmes was twofold: agriculture literature, and other (nonsystematic) reviews and studies on aquaculture that did not meet our definition of a rigorous impact evaluation.

Related to the first type of evidence, and despite the differences between aquaculture and agriculture, we expected the pathways, challenges, and possibly the direction of the impacts to be similar. While none of the relevant agriculture reviews we identified provided comparable effects from quantitative synthesis, we found that overall our findings on aquaculture interventions resonate with the results from agriculture. In line with our review, previous agriculture reviews have identified that agricultural programmes report successfully meeting production objectives (Ruel et al., 2018), whereas most of these reviews report failing to measure an impact of agriculture programmes on nutrition indicators, such as anthropometrics, BMI, micronutrient intake and general health (Bird et al., 2019; Masset et al., 2012). There were various reasons for this, including the lack of availability of quality evidence to confidently assess impact, and the long‐term nature of changes related to these health indicators. Previous reviews (Ruel & Alderman, 2013) were able to find some evidence on intermediary nutritional outcomes, such as food consumption, dietary diversity and food security scores, while a recent update identified a more consistent impact on dietary diversity, food consumption, and micronutrient intake across diverse programme types and contexts (Ruel et al., 2018). In our review, we have limited data available to synthesise similar indicators, with the exception of food consumption, on which we found a positive and significant impact.

Related to other aquaculture studies, a recent review of the aquaculture sector (Naylor et al., 2021) noted a few trends that are relevant in the context of this review. First, and in line with FAO (2020b), China is still the largest aquaculture producer, processor, and trader in the sector; second, there has been an expansion of aquaculture in South American countries; and third, there has been a growth of aquaculture value chains in and across countries in South and Southeast Asia. The majority of our included studies focuses on Asian countries, but we did not find impact evaluations looking at cross‐country interventions. Moreover, we did not find any relevant study based in China or South America. Thus, while these developments in the aquaculture sector may also reflect production approaches that go beyond the objectives of this review, the effectiveness evidence from this review was not able to represent these geographical niches in aquaculture.

While the impacts we were able to synthesise relate to three of the four outcome groups of interest defined for this review, a gap of rigorous evidence persists to assess the linkages between aquaculture and the relevant outcome groups. This may reflect that the rigorous measurement of impacts that are attributable to these programmes is a relatively new interest. However, this does not mean that aquaculture has been deprived of research efforts. While the rigorous body of evidence found in this review may not completely cover the whole picture in aquaculture, we acknowledge that there is a wealth of other types of research that have examined the relationship between aquaculture programmes and nutrition and women's empowerment, the pathways for which this review may be less informative.

In terms of the women's empowerment pathway, and following Johnson et al.'s (2018) framework, the results from this review would suggest that aquaculture interventions are reaching women, the BS and FoF programmes being examples of this. However, the data available did not allow us to assess the extent to which the positive impacts we found are benefitting or empowering women. Moreover, the qualitative data from included studies suggest that access to credit and programme support may be two mechanisms to increase participation in aquaculture programmes. These mechanisms have also been identified in aquaculture programmes that were not included in this review (Choudhury et al., 2017; Dickson et al., 2016; Farnworth et al., 2016), which linked access to grants and loan schemes, and knowledge and training on both aquaculture and gender equality to increased women's empowerment. Likewise, the qualitative analyses of the review highlight that sociocultural expectations are one of the major barriers to the participation of beneficiaries in general, and women in particular, in aquaculture activities. This notion is also echoed in work focused on aquaculture programmes not included in the review, which note similar challenges related to the expected roles of women within the aquaculture value chain and within their own households (Brugère et al., 2001; Choudhury et al., 2017; Farnworth et al., 2015; Kusakabe, 2003). When thinking about future research and programming, Kruijssen et al. (2021) recommend that interventions be aware of “subtle bias and gender‐reinforcing practices” (p. 51) that may be present in different areas of their programme design in order for aquaculture interventions to tackle these socio‐cultural factors. Similarly, Hillenbrand et al. (2014) proposed to revise the way women's empowerment is measured, so that researchers are able to reflect more accurately the social relations within aquaculture that determine the way women engage in these activities “in their own cultural terms” (p. 365).

While our quantitative results for the nutrition pathway indicate a positive and significant effect of aquaculture programmes on the consumption of fish, we were not able to identify outcomes measuring diet diversity. Studies focusing on other interventions not included in the review provide encouraging evidence to link aquaculture to nutrition outcomes. Nutrition‐sensitive fish production interventions may have the potential to improve diet quality (Ahern et al., 2020; Akter et al., 2020) by increasing the consumption of own‐produced and highly nutritious fish and improving the households' diversity of diets, particularly when the programmes include polyculture systems producing small indigenous fish species (Baten et al., 2018; Castine et al., 2017; Roos et al., 2007). More generally, this literature suggests that the adoption of nutrition‐sensitive aquaculture (i.e., prosustainability policies specifically focused on equal access to nutritious food), could provide a strong framework to allow for increases in nutrition (Gephart et al., 2021), particularly in low‐income settings (Thilsted et al., 2016).

Although this literature may seem in line with key points highlighted in the review around ways in which aquaculture programmes can affect their beneficiaries, more research is needed to evaluate these linkages in a way that observed changes in nutrition and women's empowerment can be attributed to aquaculture programmes.

## 7 AUTHORS' CONCLUSIONS

### 7.1 Implications for practice

One of the key implications of the results from this review for aquaculture policy and practice relates to the paucity of rigorous impact evaluations of relevant interventions, and the concentration of these studies on low‐income and lower‐middle‐income countries (particularly Bangladesh). From potentially relevant studies, we excluded by study design 44 interventions from 21 countries other than the ones included in this review. This suggests a lack of impact evaluations, and not necessarily of aquaculture programmes. Therefore, the aquaculture policy sector—including a wide range of organisations that fund, design, implement and evaluate aquaculture interventions—would benefit from aligning investments in aquaculture programmes with evaluation frameworks to inform what works, for whom, why and at what cost.

This review also contributes to the thinking and practice around aquaculture interventions in low‐ and middle‐income countries. Our analysis of the barriers and facilitators indicated that an increased frequency, quality and regularity of support from these interventions could affect the motivation of participants to maintain their involvement in aquaculture activities. While we were not able to contrast this finding with quantitative data around intensity or quality of support measures, this suggests that the provision of constant support to beneficiaries would be a key element in the implementation of these aquaculture programmes. While resources are always scarce and the sustainability of interventions also needs to be accounted for, future programming could incorporate this evidence to plan for suitable levels of support, as well as appropriate ways of monitoring and evaluating this to inform its impact evaluation.

### 7.2 Implications for research

Based on the evidence for this review, the main implication for future research is for the sector to encourage the production of more quality impact evaluations to assess the effectiveness of aquaculture interventions. These studies may find ways to measure a range of outcomes of interest to better inform the impact of these interventions and build up the areas where there is less evidence, namely, intermediate and main nutrition outcomes, and indicators of women's empowerment in the short and long term.

Specifically, the aquaculture body of evidence would also benefit from expanding the focus of impact evaluations into other low‐ and middle‐income countries than Bangladesh. From the studies of this review, the recent evaluations of Fadama III in Nigeria or SMART‐Fish in Indonesia are good examples of this approach. Moreover, many studies performed poorly in the risk of bias assessment as we were not able to establish from the reports that confounding issues were adequately addressed. Thus, promoting reporting standards among new evaluations would also serve to improve the quality of the body of evidence around aquaculture interventions. This could also be implemented to standardise the reporting of intervention components to facilitate a substantive comparison across programmes. Relevant examples of resources that could be adapted to report intervention components and evaluation findings in aquaculture include the CONSORT tools for reporting randomised trials of social and psychological interventions (CONSORT‐SPI; Montgomery et al., 2018) and Template for Intervention Description and Replication (TIDieR; Hoffmann et al., 2014), or the STrengthening the Reporting of OBservational studies in Epidemiology (STROBE; von Elm et al., 2008) guidelines.

In addition, while not providing a comprehensive set of recommendations, we note that much more effort could also be devoted to costing the programmes aimed to improve the aquaculture sector. Consistent with the goals of the review, cost‐effectiveness analyses are done for the purpose of knowing whether an intervention was worth doing, whether it can be implemented in a similar but different context, or if it should be extended. However, for this review, we were not able to undertake such an analysis. Broadly, we suggest the following considerations. The costs of a programme are different from the budget allocated, as not all of the budget would be devoted to implementing the programme. Intervention components should also be clearly identified, and cost guidelines from cost‐benefit analysis should be followed to account for the opportunity cost of activities in each of the components. The WorldFish report (2009) for AFP, for example, attempted to follow this approach. Moreover, investment and variable costs of the programmes should be clearly delineated. In an intervention involving private actors, such as farmers, it is possible that their net earnings may not capture the shadow cost of their activities. Thus, costing may also involve how the programme changes activities of the beneficiaries and what would be the cost of those activities. Costing is an intensive process that should be an integral part of impact analyses, and as such, aquaculture programmes could benefit from including cost‐effectiveness analysis into their evaluation frameworks.

## ACKNOWLEDGEMENTS

The review authors would like to acknowledge the advisory group of experts in the field and the funder of the review for their contributions to the review: Froukje Kruijssen, KIT Royal Tropical Institute; Harrison Charo‐Karisa, WorldFish; Richard Abila, IFAD; Vegard Iversen, University of Greenwich and 3ie; Suneetha Kadiyala, London School of Hygiene and Tropical Medicine; Jillian Waid, Potsdam Institute for Climate Impact Research; and Amy Sherwood, the Bill & Melinda Gates Foundation. Their inputs were instrumental to ensure the review is relevant for the aquaculture sector.

This study was supported, in whole or in part, by the Bill & Melinda Gates Foundation (OPP1197415). Under the grant conditions of the Foundation, a Creative Commons Attribution 4.0 Generic License has already been assigned to the Author Accepted Manuscript version that might arise from this submission.

The full data set used for the quantitative synthesis, along with its corresponding codebook, can be accessed in 3ie's Dataverse https://dataverse.harvard.edu/dataverse/3ie.

## CONTRIBUTIONS OF AUTHORS

*Content*: Marta Moratti, Constanza Gonzalez Parrao, and Shannon Shisler. *Systematic review methods*: Constanza Gonzalez Parrao, Shannon Shisler, Cem Yavuz, and Birte Snilstveit. *Statistical analysis*: Shannon Shisler and Constanza Gonzalez Parrao. *Additional analysis*: Constanza Gonzalez Parrao, Cem Yavuz, Arnab Acharya, Shannon Shisler, and Birte Snilstveit. *Information retrieval*: John Eyers, Constanza Gonzalez Parrao, and Cem Yavuz.

## DECLARATIONS OF INTEREST

The authors of the review declare having no conflict of interest related to this review, they have no financial interest in it, and have not participated in previous research or publications related to the topic of this review.

To minimise a potential bias in conducting the review, 3ie has two independent teams working on the impact evaluation of an aquaculture intervention and on this review, respectively. The funder and an advisory group of experts in the field have collaborated in the review to ensure its relevance and usability. However, they have no bearing on the implementation or reporting of this review.

## SOURCES OF SUPPORT

This review is part of an impact evaluation programme for an aquaculture intervention currently implemented by WorldFish, evaluated by 3ie, and funded by the Bill & Melinda Gates Foundation. The implementation and funding bodies, as well as the evaluation team, have no influence over the execution or reporting of the review.

## PLANS FOR UPDATING THIS REVIEW

The leading author will be responsible of updating the review 3 years after the publication of this review. If for some reason this is not possible, the leading author will communicate this to the International Development Coordinating Group.

## DIFFERENCES BETWEEN PROTOCOL AND REVIEW

For the screening process, the review team made the decision to deviate from the protocol in terms of the use of the machine learning tools available in EPPI‐Reviewer. When screening at title and abstract, the use of the priority screening function was discontinued as it was not producing the expected results. The double‐screening of records continued in the traditional way (i.e., without prioritisation of studies more likely to be included) until we had sufficient information to run the classifier model, which was successful in predicting the likelihood of inclusion of studies. We stated in the protocol that we could auto‐exclude records with a prediction below 20% if a random sample of studies within this group did not yield any relevant study. Although this was the case, we decided to screen all records with a prediction below 20% as a safety measure. We believe that this decision compensates for our deviation from the protocol, and as such, it does not pose a risk of bias or undermines the review implementation process.

The per‐protocol analysis found one outlier (standardized score = 5.32) in the full dataset (standardized scores without the outlier ranged from −2.40 to 2.10); this effect was ultimately not included in the quantitative synthesis so it was not necessary to windsorised it. Additionally, we also conducted an additional outlier analysis for each outcome model that we synthesised quantitatively. While this additional analysis was not predefined, we deemed that it would better inform the analysis of outcomes. Thus, this change would not pose a threat to the trustworthiness of the review.

## Figures and Tables

**Figure C1:**
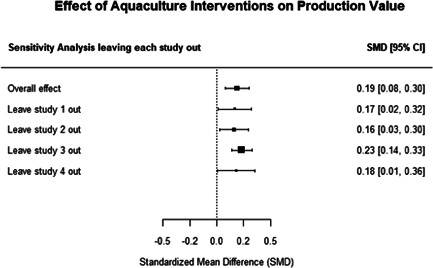
Sensitivity analysis for production value

**Figure C2:**
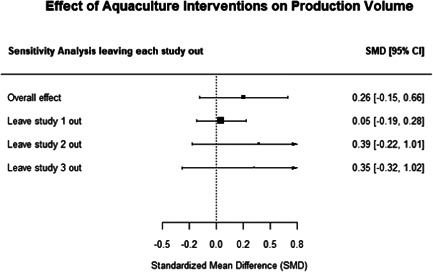
Sensitivity analysis for production volume

**Figure C3:**
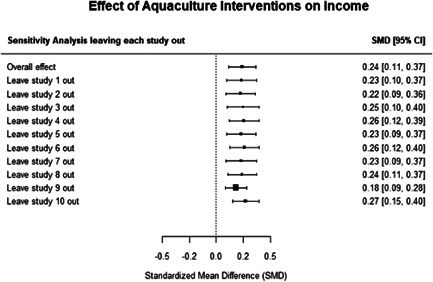
Sensitivity analysis for income

**Figure C4:**
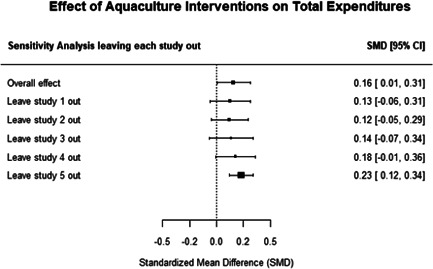
Sensitivity analysis for total expenditures

**Figure C5:**
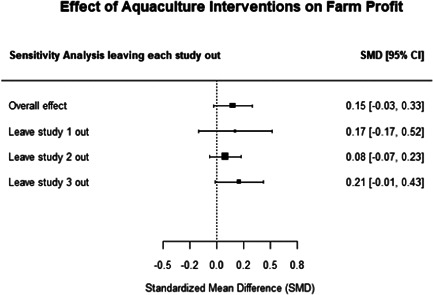
Sensitivity analysis for farm profit

**Figure C6:**
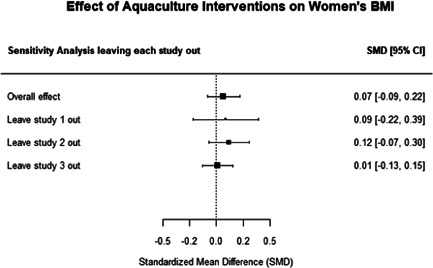
Sensitivity analysis for women's body mass index

**Figure C7:**
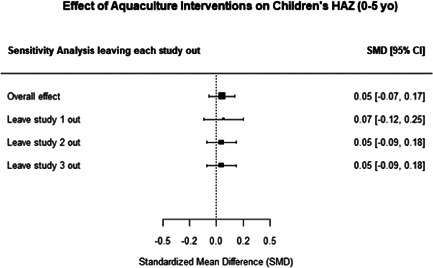
Sensitivity analysis for children's height‐for‐age

## Footnotes

^1^
This review is part of a broader aquaculture impact evaluation programme conducted by 3ie and supported by the Bill & Melinda Gates Foundation. More information on the programme is available at https://www.3ieimpact.org/our-work/agriculture/impacts-aquaculture-livelihoods-nutrition-and-womens-empowerment-bangladesh

^2^
We define value chain as the full range of activities that are required to bring a product or service from conception, through production and transformation, to delivery to final consumers, and final disposal after use (Kaplinsky & Morris, 2000).

^3^
The extent to which increased consumption comes from increases in own consumption or via higher income is an interesting research question per se. However, the effect may vary by the context, depending on which activities one is engaged with along the value chains (producing vs. nonproducing role), the type of aquaculture organisms (small vs. export‐led types), the welfare position of the household, and the accessibility and availability of aquaculture in the markets. A study on food consumption in Bangladesh (Roos, 2001) found, for example, that fish consumed from fish produced by own‐pond aquaculture only contributed 1%–11% of the total amount of fish consumed at household level, and fish sold in the markets is the single most important source of fish (57%–69%, depending on the season) for households with and without fish ponds.

^4^
Following van Eerdewijk et al. (2017), we refer to empowerment as the expansion of choice and strengthening of voice through the transformation of power relations, so women and girls have more control over their lives and futures.

^5^
This source was used as the development database. A full example of the search strategy for this database is detailed in Appendix A.1.

^6^
Egger's test for publication bias was conducted only for outcomes with at least 10 independent effect sizes.

^7^
Appendix C.1 includes a summary of the main meta‐analysis results. Appendix C.2 presents funnel plots for outcomes where Egger's test for publication bias is statistically significant. Appendix C.2 includes forest plots for sensitivity analyses using the leave‐one‐out approach for outcomes with at least three effects sizes. Conducting sensitivity analyses excluding studies with high risk of bias was not possible in this review given the relatively small number of included studies, of which many were assessed to have a high risk of bias. Finally, Appendix C.4 includes a summary table for the moderator analyses results.

^8^
Production volume includes measures related to the amount of fish production, productivity measures (e.g., kg/m^2^), mortality, and feed conversion ratio. None of these measures is reported for two programmes, hence, we have grouped them under the same outcome.

^9^
Talukder and Green (2014) also reported an income outcome for Fish on Farms; however, the sample used in the analyses is not clearly reported, and we could not calculate an effect size confidently. Therefore, this outcome could not be included in the synthesis.

^10^
Small farms were defined as operating one pond of <0.08 acres, while large farms operate at least two ponds covering 0.08 acres or more (Amankwah et al., 2018, p. 424).

^11^
We identified a further two papers with potentially relevant information: Hillenbrand et al. (2014) and Kuijpers (2020). These papers were not ultimately used in this analysis as it was not possible to synthesise them with other findings.

^12^
All conversions from average effects to USD have been adjusted using the U.S. Bureau of Labor Statistics' consumer price index inflation calculator (BLS, n.d.). Additionally, whenever possible, we used data from studies that have some concerns related to their risk of bias (instead of studies with a high risk of bias).

^13^
We assessed 22 papers as three included studies (of 19) covered two aquaculture programmes each.

## APPENDIX A IMPLEMENTATION OF THE REVIEW

### A.1. Search strategy

We conducted our initial search between November 3 and 10, November 2020, which resulted in 14,256 records being identified: 10,179 from academic databases and 4077 from grey literature sources.

For academic databases, we used a combination of key terms and indexing searching. The review team tested the strategy iteratively to achieve a balance between comprehensiveness and feasibility, and a full example is presented below. We used these key term groups as the basis for the search in grey literature sources. However, the strategy had to be adapted to each source depending on its functionalities. We began the grey literature search with a baseline search string, and added to or subtracted from it depending on the restrictions imposed by the source website. The basic strategy consisted of a term related to aquaculture being paired with a term relating to our primary outcomes, for example, nutrition or income. At the most basic level, some websites only allowed to search for the term “aquaculture”, whereas most allowed us to search for “aquaculture” alongside terms indicating the outcomes we were searching for. The functionality of the websites dictated the exact string we could use, as some search engines would not allow the use of Boolean functions. In contrast, larger repositories, such as that of USAID, allowed us to enter an extensive Boolean search.

Full search strategy used in development database: CAB Abstracts (EBSCO)

**Table:**

S26	S24 OR S25
	4648
S25	S7 AND S18 AND S19 AND S23
	2683
S24	S4 AND S18 AND S19 AND S23
	4530
S23	S20 OR S21 OR S22
	4,269,556
S22	TI ((income* or livelihood* or production or productivity or productive or consumption or pay or payment* or earning* or remunerat* or profit* or salar* or wage or wages or expenditure or "food security" or cost‐utility or ((cost* or economic*) N3 (benefit* or effect* or evaluat*)) or (poverty N3 (reduc* or alleviat*)) or extension or training or (knowledge N4 (practis* or practic*))) OR AB ((income* or livelihood* or production or productivity or productive or consumption or pay or payment* or earning* or remunerat* or profit* or salar* or wage or wages or expenditure or "food security" or cost‐utility or ((cost* or economic*) N3 (benefit* or effect* or evaluat*)) or (poverty N3 (reduc* or alleviat*)) or extension or training or (knowledge N4 (practis* or practic*))) OR SU ((income* or livelihood* or production or productivity or productive or consumption or pay or payment* or earning* or remunerat* or profit* or salar* or wage or wages or expenditure or "food security" or cost‐utility or ((cost* or economic*) N3 (benefit* or effect* or evaluat*)) or (poverty N3 (reduc* or alleviat*)) or extension or training or (knowledge N4 (practis* or practic*)))
	1,796,348
S21	TI ((gender* or empower* or disempower* or inequit* or inequalit* or equalit* or disadvantage* or marginali* or discriminat* or vulnerab* or barrier* or "self help" or control or controlling or ownership* or (decision* N3 (make or maker* or making or made)) or confident or confidence or power* or access* or norm or norms or women or female*)) OR AB ((gender* or empower* or disempower* or inequit* or inequalit* or equalit* or disadvantage* or marginali* or discriminat* or vulnerab* or barrier* or "self help" or control or controlling or ownership* or (decision* N3 (make or maker* or making or made)) or confident or confidence or power* or access* or norm or norms or women or female*)) OR SU ((gender* or empower* or disempower* or inequit* or inequalit* or equalit* or disadvantage* or marginali* or discriminat* or vulnerab* or barrier* or "self help" or control or controlling or ownership* or (decision* N3 (make or maker* or making or made)) or confident or confidence or power* or access* or norm or norms or women or female*))
	2,718,403
S20	TI ((nutritio* or diet* or nourishment or fish‐based or "food intake" or "food consumption" or (food* N2 (varie* or divers*)) or ((eat* or consum*) N3 fish) or weight‐for‐height or weight‐for‐length or height‐for‐age or weight‐for‐age or "body mass index" or BMI or anthropometr*)) OR AB ((nutritio* or diet* or nourishment or fish‐based or "food intake" or "food consumption" or (food* N2 (varie* or divers*)) or ((eat* or consum*) N3 fish) or weight‐for‐height or weight‐for‐length or height‐for‐age or weight‐for‐age or "body mass index" or BMI or anthropometr*)) OR SU ((nutritio* or diet* or nourishment or fish‐based or "food intake" or "food consumption" or (food* N2 (varie* or divers*)) or ((eat* or consum*) N3 fish) or weight‐for‐height or weight‐for‐length or height‐for‐age or weight‐for‐age or "body mass index" or BMI or anthropometr* or "height‐weight tables"))
	924,075
S19	TI (("random* control* trial*" OR "random* trial*" OR RCT OR "cluster random* trial" OR "propensity score matching" OR PSM OR "regression discontinuity design" OR RDD OR "difference in difference*" OR "control* random* trial*" OR "case control" OR matching OR "interrupted time series" OR "random* allocation*" OR (random* N3 (allocat* OR select*)) OR "instrumental variable*" OR evaluation OR assessment OR ((quantitative OR "comparison group" OR counterfactual OR "counter factual" OR counter‐factual OR experiment*) N3 (design OR study OR analysis)) OR QED OR quasi‐experiment*)) OR AB (("random* control* trial*" OR "random* trial*" OR RCT OR "cluster random* trial" OR "propensity score matching" OR PSM OR "regression discontinuity design" OR RDD OR "difference in difference*" OR "control* random* trial*" OR "case control" OR matching OR "interrupted time series" OR "random* allocation*" OR (random* N3 (allocat* OR select*)) OR "instrumental variable*" OR evaluation OR assessment OR ((quantitative OR "comparison group" OR counterfactual OR "counter factual" OR counter‐factual OR experiment*) N3 (design OR study OR analysis)) OR QED OR quasi‐experiment*)) OR SU (("random* control* trial*" OR "random* trial*" OR RCT OR "cluster random* trial" OR "propensity score matching" OR PSM OR "regression discontinuity design" OR RDD OR "difference in difference*" OR "control* random* trial*" OR "case control" OR matching OR "interrupted time series" OR "random* allocation*" OR (random* N3 (allocat* OR select*)) OR "instrumental variable*" OR evaluation OR assessment OR ((quantitative OR "comparison group" OR counterfactual OR "counter factual" OR counter‐factual OR experiment*) N3 (design OR study OR analysis)) OR QED OR quasi‐experiment*))
	1,196,472
S18	S8 OR S9 OR S10 OR S11 OR S12 OR S13 OR S14 OR S15 OR S16 OR S17
	4,387,938
S17	GL(Afghanistan OR Albania OR Algeria OR Angola OR Antigua OR Barbuda OR Argentina OR Armenia OR Armenian OR Aruba OR Azerbaijan OR Bahrain OR Bangladesh OR Barbados OR Benin OR Belize OR Bhutan OR Bolivia OR Botswana OR Brazil OR Brasil OR "Burkina Faso" OR "Burkina Fasso" OR "Upper Volta" OR Burundi OR Urundi OR Cambodia OR "Khmer Republic" OR Kampuchea OR Cameroon OR Cameroons OR Cameron OR Camerons OR "Cape Verde" OR "Central African Republic" OR Chad OR Chile OR China OR Colombia OR Comoros OR "Comoro Islands" OR Comores OR Mayotte OR Congo OR Zaire OR "Costa Rica" OR "Cote d'Ivoire" OR "Ivory Coast" OR Cuba OR "Djibouti" OR "French Somaliland" OR Dominica OR "Dominican Republic" OR "East Timor" OR "East Timur" OR "Timor Leste" OR Ecuador OR Egypt OR "United Arab Republic" OR "El Salvador" OR Eritrea OR Ethiopia OR Fiji OR Gabon OR "Gabonese Republic" OR Gambia OR Gaza OR "Georgia Republic" OR "Georgian Republic" OR Ghana OR "Gold Coast" OR Grenada OR Guatemala OR Guinea OR Guam OR Guiana OR Guyana OR Haiti OR Honduras OR India OR Maldives OR Indonesia OR Iran OR Iraq OR Jamaica OR Jordan OR Kazakhstan OR Kazakh OR Kenya OR Kiribati OR Korea OR Kosovo OR Kyrgyzstan OR Kirghizia OR "Kyrgyz Republic" OR Kirghiz OR Kirgizstan OR "Lao PDR" OR Laos OR Lebanon OR Lesotho OR Basutoland OR Liberia OR Libya OR Madagascar OR "Malagasy Republic" OR Malaysia OR Malaya OR Malay OR Sabah OR Sarawak OR Malawi OR Nyasaland OR Mali OR "Marshall Islands" OR Mauritania OR Mauritius OR "Agalega Islands" OR Mexico OR Micronesia OR "Middle East" OR Moldova OR Moldovia OR Moldovian OR Mongolia OR Montenegro OR Morocco OR Ifni OR Mozambique OR Myanmar OR Myanma OR Burma OR Namibia OR Nepal OR Antilles OR "New Caledonia" OR Nicaragua OR Niger OR Nigeria OR "Mariana Islands" OR Oman OR Muscat OR Pakistan OR Palau OR Palestine OR Panama OR Paraguay OR Peru OR Philippines OR Philipines OR Phillipines OR Phillippines OR "Puerto Rico" OR Rwanda OR Ruanda OR "Saint Kitts" OR "St Kitts" OR Nevis OR "Saint Lucia" OR "St Lucia" OR "Saint Vincent" OR "St Vincent" OR "Grenadines" OR "Samoa" OR "Samoan Islands" OR "Navigator Island" OR "Navigator Islands" OR "Sao Tome" OR "Saudi Arabia" OR Senegal OR Seychelles OR "Sierra Leone" OR "Sri Lanka" OR "Solomon Islands" OR Somalia OR Sudan OR Suriname OR Surinam OR Swaziland OR Syria OR Tajikistan OR Tadzhikistan OR Tadjikistan OR Tadzhik OR Tanzania OR Thailand OR Togo OR "Togolese Republic" OR Tonga OR Trinidad OR Tobago OR Tunisia OR Turkey OR Turkmenistan OR Turkmen OR Uganda OR Ukraine OR Uruguay OR Uzbekistan OR Uzbek OR Vanuatu OR "New Hebrides" OR Venezuela OR Vietnam OR "Viet Nam" OR "West Bank" OR Yemen OR Zambia OR Zimbabwe OR Jamahiriya OR Jamahiryria OR Libia OR Mocambique OR Principe OR Syrian OR "Indian Ocean" OR Melanesia OR "Western Sahara")
	2,115,450
S16	TI(Afghanistan OR Albania OR Algeria OR Angola OR Antigua OR Barbuda OR Argentina OR Armenia OR Armenian OR Aruba OR Azerbaijan OR Bahrain OR Bangladesh OR Barbados OR Benin OR Belize OR Bhutan OR Bolivia OR Botswana OR Brazil OR Brasil OR "Burkina Faso" OR "Burkina Fasso" OR "Upper Volta" OR Burundi OR Urundi OR Cambodia OR "Khmer Republic" OR Kampuchea OR Cameroon OR Cameroons OR Cameron OR Camerons OR "Cape Verde" OR "Central African Republic" OR Chad OR Chile OR China OR Colombia OR Comoros OR "Comoro Islands" OR Comores OR Mayotte OR Congo OR Zaire OR "Costa Rica" OR "Cote d'Ivoire" OR "Ivory Coast" OR Cuba OR "Djibouti" OR "French Somaliland" OR Dominica OR "Dominican Republic" OR "East Timor" OR "East Timur" OR "Timor Leste" OR Ecuador OR Egypt OR "United Arab Republic" OR "El Salvador" OR Eritrea OR Ethiopia OR Fiji OR Gabon OR "Gabonese Republic" OR Gambia OR Gaza OR "Georgia Republic" OR "Georgian Republic" OR Ghana OR "Gold Coast" OR Grenada OR Guatemala OR Guinea OR Guam OR Guiana OR Guyana OR Haiti OR Honduras OR India OR Maldives OR Indonesia OR Iran OR Iraq OR Jamaica OR Jordan OR Kazakhstan OR Kazakh OR Kenya OR Kiribati OR Korea OR Kosovo OR Kyrgyzstan OR Kirghizia OR "Kyrgyz Republic" OR Kirghiz OR Kirgizstan OR "Lao PDR" OR Laos OR Lebanon OR Lesotho OR Basutoland OR Liberia OR Libya OR Madagascar OR "Malagasy Republic" OR Malaysia OR Malaya OR Malay OR Sabah OR Sarawak OR Malawi OR Nyasaland OR Mali OR "Marshall Islands" OR Mauritania OR Mauritius OR "Agalega Islands" OR Mexico OR Micronesia OR "Middle East" OR Moldova OR Moldovia OR Moldovian OR Mongolia OR Montenegro OR Morocco OR Ifni OR Mozambique OR Myanmar OR Myanma OR Burma OR Namibia OR Nepal OR Antilles OR "New Caledonia" OR Nicaragua OR Niger OR Nigeria OR "Mariana Islands" OR Oman OR Muscat OR Pakistan OR Palau OR Palestine OR Panama OR Paraguay OR Peru OR Philippines OR Philipines OR Phillipines OR Phillippines OR "Puerto Rico" OR Rwanda OR Ruanda OR "Saint Kitts" OR "St Kitts" OR Nevis OR "Saint Lucia" OR "St Lucia" OR "Saint Vincent" OR "St Vincent" OR "Grenadines" OR "Samoa" OR "Samoan Islands" OR "Navigator Island" OR "Navigator Islands" OR "Sao Tome" OR "Saudi Arabia" OR Senegal OR Seychelles OR "Sierra Leone" OR "Sri Lanka" OR "Solomon Islands" OR Somalia OR Sudan OR Suriname OR Surinam OR Swaziland OR Syria OR Tajikistan OR Tadzhikistan OR Tadjikistan OR Tadzhik OR Tanzania OR Thailand OR Togo OR "Togolese Republic" OR Tonga OR Trinidad OR Tobago OR Tunisia OR Turkey OR Turkmenistan OR Turkmen OR Uganda OR Ukraine OR Uruguay OR Uzbekistan OR Uzbek OR Vanuatu OR "New Hebrides" OR Venezuela OR Vietnam OR "Viet Nam" OR "West Bank" OR Yemen OR Zambia OR Zimbabwe OR Jamahiriya OR Jamahiryria OR Libia OR Mocambique OR Principe OR Syrian OR "Indian Ocean" OR Melanesia OR "Western Sahara")
	781,645
S15	AB(Afghanistan OR Albania OR Algeria OR Angola OR Antigua OR Barbuda OR Argentina OR Armenia OR Armenian OR Aruba OR Azerbaijan OR Bahrain OR Bangladesh OR Barbados OR Benin OR Belize OR Bhutan OR Bolivia OR Botswana OR Brazil OR Brasil OR "Burkina Faso" OR "Burkina Fasso" OR "Upper Volta" OR Burundi OR Urundi OR Cambodia OR "Khmer Republic" OR Kampuchea OR Cameroon OR Cameroons OR Cameron OR Camerons OR "Cape Verde" OR "Central African Republic" OR Chad OR Chile OR China OR Colombia OR Comoros OR "Comoro Islands" OR Comores OR Mayotte OR Congo OR Zaire OR "Costa Rica" OR "Cote d'Ivoire" OR "Ivory Coast" OR Cuba OR "Djibouti" OR "French Somaliland" OR Dominica OR "Dominican Republic" OR "East Timor" OR "East Timur" OR "Timor Leste" OR Ecuador OR Egypt OR "United Arab Republic" OR "El Salvador" OR Eritrea OR Ethiopia OR Fiji OR Gabon OR "Gabonese Republic" OR Gambia OR Gaza OR "Georgia Republic" OR "Georgian Republic" OR Ghana OR "Gold Coast" OR Grenada OR Guatemala OR Guinea OR Guam OR Guiana OR Guyana OR Haiti OR Honduras OR India OR Maldives OR Indonesia OR Iran OR Iraq OR Jamaica OR Jordan OR Kazakhstan OR Kazakh OR Kenya OR Kiribati OR Korea OR Kosovo OR Kyrgyzstan OR Kirghizia OR "Kyrgyz Republic" OR Kirghiz OR Kirgizstan OR "Lao PDR" OR Laos OR Lebanon OR Lesotho OR Basutoland OR Liberia OR Libya OR Madagascar OR "Malagasy Republic" OR Malaysia OR Malaya OR Malay OR Sabah OR Sarawak OR Malawi OR Nyasaland OR Mali OR "Marshall Islands" OR Mauritania OR Mauritius OR "Agalega Islands" OR Mexico OR Micronesia OR "Middle East" OR Moldova OR Moldovia OR Moldovian OR Mongolia OR Montenegro OR Morocco OR Ifni OR Mozambique OR Myanmar OR Myanma OR Burma OR Namibia OR Nepal OR Antilles OR "New Caledonia" OR Nicaragua OR Niger OR Nigeria OR "Mariana Islands" OR Oman OR Muscat OR Pakistan OR Palau OR Palestine OR Panama OR Paraguay OR Peru OR Philippines OR Philipines OR Phillipines OR Phillippines OR "Puerto Rico" OR Rwanda OR Ruanda OR "Saint Kitts" OR "St Kitts" OR Nevis OR "Saint Lucia" OR "St Lucia" OR "Saint Vincent" OR "St Vincent" OR "Grenadines" OR "Samoa" OR "Samoan Islands" OR "Navigator Island" OR "Navigator Islands" OR "Sao Tome" OR "Saudi Arabia" OR Senegal OR Seychelles OR "Sierra Leone" OR "Sri Lanka" OR "Solomon Islands" OR Somalia OR Sudan OR Suriname OR Surinam OR Swaziland OR Syria OR Tajikistan OR Tadzhikistan OR Tadjikistan OR Tadzhik OR Tanzania OR Thailand OR Togo OR "Togolese Republic" OR Tonga OR Trinidad OR Tobago OR Tunisia OR Turkey OR Turkmenistan OR Turkmen OR Uganda OR Ukraine OR Uruguay OR Uzbekistan OR Uzbek OR Vanuatu OR "New Hebrides" OR Venezuela OR Vietnam OR "Viet Nam" OR "West Bank" OR Yemen OR Zambia OR Zimbabwe OR Jamahiriya OR Jamahiryria OR Libia OR Mocambique OR Principe OR Syrian OR "Indian Ocean" OR Melanesia OR "Western Sahara")
	1,417,604
S14	TI ((developing or less* N1 developed or "under developed" or underdeveloped or "middle income" or low* N1 income or underserved or "under served" or deprived or poor*) N1 (countr* or nation* or population* or world)) OR AB ((developing or less* N1 developed or "under developed" or underdeveloped or "middle income" or low* N1 income or underserved or "under served" or deprived or poor*) N1 (countr* or nation* or population* or world)) OR SU ((developing or less* N1 developed or "under developed" or underdeveloped or "middle income" or low* N1 income or underserved or "under served" or deprived or poor*) N1 (countr* or nation* or population* or world))
	4,191,490
S13	TI ((developing or less* N1 developed or "under developed" or underdeveloped or "middle income" or low* N1 income) N1 (economy or economies)) OR AB ((developing or less* N1 developed or "under developed" or underdeveloped or "middle income" or low* N1 income) N1 (economy or economies)) OR SU ((developing or less* N1 developed or "under developed" or underdeveloped or "middle income" or low* N1 income) N1 (economy or economies))
	2310
S12	TI (low* N1 (gdp or gnp or "gross domestic" or "gross national")) OR AB (low* N1 (gdp or gnp or "gross domestic" or "gross national")) OR SU (low* N1 (gdp or gnp or "gross domestic" or "gross national"))
	160
S11	TI (low 3 middle N3 countr*) OR AB (low 3 middle N3 countr*) OR SU (low 3 middle N3 countr*)
	9
S10	TI ((lmic or lmics or "third world" or "lami country" or "lami countries")) OR AB ((lmic or lmics or "third world" or "lami country" or "lami countries")) OR SU ((lmic or lmics or "third world" or "lami country" or "lami countries"))
	43,978
S9	TI (("transitional country" or "transitional countries")) OR AB (("transitional country" or "transitional countries")) OR SU (("transitional country" or "transitional countries"))
	144
S8	TI (Africa or Asia or Caribbean or "West Indies" or "South America" or "Latin America" or "Central America") OR AB (Africa or Asia or Caribbean or "West Indies" or "South America" or "Latin America" or "Central America") OR SU (Africa or Asia or Caribbean or "West Indies" or "South America" or "Latin America" or "Central America") OR GL (Africa or Asia or Caribbean or "West Indies" or "South America" or "Latin America" or "Central America")
	2,404,838
S7	S5 OR S6
	46,203
S6	DE "salmon culture" or DE "frog culture" or DE "turtle culture"
	890
S5	TI (((fish* or tilapia or carp or shrimp or mussel* or shellfish or crustacean* or mollusc* or rice‐fish or frog* or turtle* or seaweed) N2 ((farm* or culture or small‐scale or pond or pond* or cage*))) OR AB (((fish* or tilapia or carp or shrimp or mussel* or shellfish or crustacean* or mollusc* or rice‐fish or frog* or turtle* or seaweed) N2 ((farm* or culture or small‐scale or pond or pond* or cage*))) OR SU (((fish* or tilapia or carp or shrimp or mussel* or shellfish or crustacean* or mollusc* or rice‐fish or frog* or turtle* or seaweed) N2 ((farm* or culture or small‐scale or pond or pond* or cage*))
	46,203
S4	S1 OR S2 OR S3
	143,695
S3	CC "MM120"
	131,344
S2	DE "aquaculture" OR DE "brackishwater aquaculture" OR DE "fish culture" OR DE "freshwater aquaculture" OR DE "marine aquaculture" OR DE "agropisciculture" OR DE "shellfish culture" OR DE "wastewater aquaculture" OR DE "growout ponds" OR DE "fish production"
	60,077
S1	TI ((aquaculture or ((fish* or shellfish or rice‐fish or seaweed) N3 (farm* or culture or small‐scale or cage*)) or "pond culture" or polyculture or fishpond* or Mallahin or fisherwomen or pisciculture)) OR AB ((aquaculture or ((fish* or shellfish or rice‐fish or seaweed) N3 (farm* or culture or small‐scale or cage*)) or "pond culture" or polyculture or fishpond* or Mallahin or fisherwomen or pisciculture))
	48,289

### A.2. Screening process with machine learning tools

As outlined in the protocol for this review, we utilised two machine learning functions of EPPI‐Reviewer during the screening process. The first function, priority screening, allowed us to code studies and EPPI to use those decisions to prioritise the records being screened by relevance. The second function, the classifier model, allowed us to group records into probabilities of their inclusion at the title and abstract screening stage. The following sections outline the process we utilised for screening papers.

To train consultants on screening, the core review team produced three batches of 60 records to be screened at the title and abstract stage, for each consultant to practice. If a consultant had at least 95% agreement with the core team's decision at the include/exclusion level, then they began with priority screening. If a consultant did not reach this threshold, they carried on with the following training batch until they did. One consultant required a fourth batch, which was produced by one member of the core team.

We began title and abstract screening using EPPI's priority screening function. It was set up in such a way that two coders would code each review paper. The priority screening function did not work as anticipated, allegedly due to how it was set up in EPPI. As one consultant had coded around 2500 papers, we chose to stop using priority screening and instead allocated these records between all other coders. Then, this function was used only for 20% of total papers from the academic and grey literature search. Once this group of records was completed, we used these papers to run the classifier model to order and prioritise the remaining studies. The classifier function requires a number of records to be screened, and uses the include and exclude codes assigned to the completed records to make a judgement on the inclusion probability of the remaining unscreened records. Because reconciliations for this group of records was based on the include/exclusion level, instead of specific exclusion criteria, it became apparent that the classifier model was unable to identify papers of interest due to the noise surrounding this reconciliation mode. While during the training stage we based reconciliations at the specific exclusion codes, this was not possible to implement for the group of 20% of studies due to time and resource constraints. To overcome this issue, we used the training studies, which had been reconciled at the specific code level. Therefore, once we based the classifier model on the training batches plus all included studies from the 20% group of screened records, the classifier was successful.

We then continued to screen based around the probability range of studies. Table A1 shows the number of records classified into each probability range, and the number of records included after double‐screening at title and abstract. Originally, we had planned to screen a random sample of records under 20% probability of inclusion, and if no studies were included, we would automatically exclude the rest. After performing this task, and despite no papers being included, we decided to screen all papers to test the classifier model. As no papers under 20% probability were included, we were able to confirm the success of the classifier model.

**Table A1:** Classifier model results for the T&A screening stage

Classifier range of inclusion	Total number of unscreened records	Studies included at T&A stage
90%–99%	389	48
80%–89%	469	12
70%–79%	464	19
60%–69%	524	7
50%–59%	569	8
40%–49%	706	5
30%–39%	817	9
20%–29%	1177	3
10%–19%	1688	0
0%–10%	2312	0

### A.3. Backwards and forward citation tracking

After screening records at full‐text, we identified papers for which we would conduct backwards and forwards citation tracking. Not only did we conduct this on the included records, but during the screening process we also coded relevant papers and reviews which were not includable to check their references. Table A2 provides a list of the reviews which were screened at this stage: six were identified at the protocol stage, whereas seven of them where identified in the screening process.

**Table A2:** Relevant reviews for backwards citation tracking

Author	Year	Title of review	Identified at
Bene et al.	2016	Contribution of Fisheries and Aquaculture to Food Security and Poverty Reduction: Assessing the Current Evidence	Screening
Bird et al.	2019	Interventions in agriculture for nutrition outcomes: A systematic review focused on South Asia	Protocol
d'Armengol et al	2018	A systematic review of co‐managed small‐scale fisheries: Social diversity and adaptive management improve outcomes	Protocol
DFID	2014	Can agriculture interventions promote nutrition? Agriculture and nutrition evidence paper	Screening
Gambelli et al.	2019	Economic performance of organic aquaculture: A systematic review	Protocol
Joffre et al.	2017	Increasing productivity and improving livelihoods in aquatic agricultural systems: a review of interventions	Screening
Kruijssen et al.	2018	Gender and aquaculture value chains: A review of key issues and implications for research	Protocol
Loneragan and Stacey	2018	SRA small‐scale fisheries in Indonesia: benefits to households, the roles of women, and opportunities for improving livelihoods	Screening
Masset et al.	2012	Effectiveness of agricultural interventions that aim to improve nutritional status of children: systematic review	Protocol
Phillips et al	2009	Review of environmental impact assessment and monitoring in aquaculture in Asia‐Pacific	Screening
Ruel et al.	2018	Nutrition sensitive agriculture: What have we learned so far?	Protocol
Ruiz Campo and Zuniga‐Jara	2018	Reviewing capital cost estimations in aquaculture	Screening
Stevenson and Irz	2009	Is aquaculture development an effective tool for poverty alleviation? A review of theory and evidence	Screening

Backwards citation tracking was carried out by handsearching the reference list of the relevant records and identifying firstly whether a reference is of potential relevance, and second, whether the study had already been located in our previous search. Team members were located in the UK when carrying out the backwards and forward citation tracking, as well as the search for additional documentation to answer RQ4 and RQ5.

Forwards citation tracking was carried out using Google Scholar. We carried out the original backward and forwards citation tracking in December 2020, which resulted in the identification of 317 new records: 305 from forwards citation tracking, and 12 from backwards citation tracking. A second search was carried out in January 2021 after a new paper had been included in the review, which resulted in no new papers from backwards citation but a further 20 papers being identified from forwards citation tracking. We later identified one additional includable paper after our blog post, but given that this was an unpublished report, there was no need to carry out forward citation, and backwards search did not identify any additional papers. After identifying included papers, we located records that had referenced our included paper. This process was repeated for all our included papers and the list of which papers had cited our included papers was composed. A total of 36 of these records were duplicates of those which had previously been included and so an additional 301 records were screened. The final stage of our search was the publication of a blog on 3ie's website in January 2021 describing the included records we had found so far and asking for references to any additional records. This resulted in the identification of three further papers.

Of the 21 included papers, 15 were identified in the academic database search, three were identified in the grey literature search, two were identified from backwards citation tracking, and one was identified through the publication of an institutional blog calling for additional evidence.

### A.4. Study inclusion and exclusion criteria

The inclusion and exclusion criteria we used for this review can be summarised by the population, interventions, comparators, outcomes and study designs (PICOS) framework, shown in Table A3. In addition, we excluded papers published before 1980 but had no restrictions on the publication language.

**Table A3:** PICOS framework for identifying relevant literature

Population	The unit of analysis included individuals, households, villages, municipalities, or community‐based organisations. The study sample must have been based in a low‐ and middle‐income country. There was no restriction on location of study in terms or rural or urban areas and there were no demographic restrictions.
Interventions	We included all aquaculture activities related to farming fish and other aquatic organisms (e.g., seaweed), based in ponds, cages, and other aquaculture systems, involving land‐based and water‐based aquaculture, and covering the various stages of its value chain for which there is relevant evidence.
Comparators	Any type of comparison group (i.e., business as usual, or another, different aquaculture intervention)
Outcomes	Primary outcomes: productivity, income, nutrition, and empowerment. Secondary outcomes: any outcome reported which could not be categorised by the above groups.
Study designs	To address research questions 1–3, we included evaluations that use an experimental or quasi‐experimental design to robustly measure a change in outcomes that is attributed to an intervention as is compared to an appropriate counterfactual. We considered evaluations of pilot studies aimed to be scaled up. However, efficacy studies, feasibility studies, acceptability studies, literature reviews and systematic reviews were not included as primary studies.

As well as our PICOS framework, we defined a set of codes to be used at both title and abstract and full‐text screening. Tables A4 and A5 describe these codes, which were the exact instructions provided to coders when conducting the screening process, and show the number of studies excluded using each criterion.

**Table A4:** Exclusion criteria used at title and abstract (T&A) screening

Exclusion criteria	Description	Number of records excluded at T&A
Publication year	Use this code if the study was published before 1980	20
Low‐ and middle‐income country	Use this code if the intervention took place in a country that was classified as a high‐income country at the time the intervention was implemented. The classification is based on the World Bank's Country and Lending Group Classification	651
Study type	Use this code if the paper is focused on aquaculture but is a literature or systematic review	6174
	We also exclude papers which are not primary studies, this would include briefs and conference proceedings	
Efficacy study	Efficacy studies are excluded, so use this code if the intervention is clearly set under ideal control conditions. For example, this could be a study set within a lab or where lab conditions are carried out in the field. Pilot studies with the intention of being scaled up are included	1704
Intervention	Use this code if there is no intervention or if the intervention does not fall within the intervention framework specified within the protocol	2418
Study design	Use this code if the study does not use one of the designs specified within the protocol, or if there is no comparison group. Often it is difficult to judge this at the T&A stage so be inclusive (i.e., if there is no information to clearly exclude the study, then don't)	802

**Table A5:** Exclusion criteria used at full‐text screening

Exclusion criteria	Description	Number of records excluded at full‐text
Publication year	Use this code if the study was published before 1980	2
Low‐ and middle‐income country	Use this code if the intervention took place in a country that was classified as a high‐income country at the time the intervention was implemented. The classification is based on the World Bank's Country and Lending Group Classification	0
Study type	Use this code if the paper is focused on aquaculture but is a literature or systematic review	49
	Efficacy studies are excluded, so use this code if the intervention is clearly set under ideal control conditions. For example, this could be a study set within a lab or where lab conditions are carried out in the field. Pilot studies with the intention of being scaled up are included	
Intervention	Use this code if there is no intervention or if the intervention does not fall within the intervention framework specified within the protocol	35
Study design	Use this code if the study does not use one of the designs specified within the protocol, or if there is no comparison group	70

A further discussion as to why certain papers were excluded is warranted. The main reason for exclusion at the full‐text stage was study design (*n* = 70), which can be further broken down into four issues. Twenty‐nine papers were excluded as they used an inappropriate evaluation method; for instance, many papers simply reported means and frequencies without selecting an appropriate counterfactual (e.g., Dey et al., 2007; Henri‐Ukoha et al., 2011). Seventeen papers were excluded as their analysis did not include a counterfactual (e.g., Laxmi et al., 2015; Rahman et al., 2010). Sixteen papers were excluded as their analysis was purely descriptive, meaning there was no quantitative assessment of an effect of an intervention (e.g., Hallman et al., 2007; Ike & Roseline, 2007). Finally, eight papers were excluded as they selected an inappropriate counterfactual. The most interesting of these being Kumar and Quisumbing (2011), a paper linked to one of our included studies (Quisumbing & Kumar, 2011). Despite both papers using the same data for their analysis, the way the analysis is carried out in Kumar and Quisumbing (2011) means that the counterfactual group contains programme beneficiaries. In contrast, Quisumbing and Kumar (2011) sets up the counterfactual to exclude programme beneficiaries by clearly making a distinction between long‐term project beneficiaries and short‐term project beneficiaries. To exemplify the inclusion and exclusion criteria for this review, Table A6 presents the rationale for specific inclusion decisions made for similar studies in terms of the aquaculture programmes covered.

**Table A6:** Examples of inclusion and exclusion decisions

Included paper	Excluded paper	Rationale of decision
Quisumbing and Kumar (2011); Does social capital build women's assets? The long‐term impacts of group‐based and individual dissemination of agricultural technology in Bangladesh	Kumar and Quisumbing (2011); Access, adoption, and diffusion: understanding the long‐term impacts of improved vegetable and fish technologies in Bangladesh	These two related papers use the same set of data to look at different outcomes related to both the Mymensingh Aquaculture Extension Project and the NGO Banchte Shekha programme. The data they use is split between three groups of participants, early adopters, late adopters and late nonadopters. Whereas Quisumbing and Kumar (2011) analyse the difference between early adopters and late adopters against nonadopters, Kumar and Quisumbing (2011) analyse the difference between early adopters against late adopters and late nonadopters. Therefore, their control is made up partly of those who received the intervention, and this was not valid for inclusion in the review
Alawode and Oluwatayo (2019); Development Outcomes of Fadama III among Fish Farmers in Nigeria: Evidence from Lagos State	Umar (2012); Assessment of the adoption rate of technologies among fadama III farmers in Adamawa State, Nigeria	Both papers looked at outcomes related to fish farmers who participated in the Fadama III programme in Nigeria. Though each paper has yield as an outcome, Umar (2012) was excluded as it does not assess the impact effects of the programme. Rather, it analyses the effects that participation had on technology adoption, and in‐turn the effects technology adoption had on yield. Technology adoption was not an outcome of interest and so this paper was excluded. Alawode and Oluwatayo (2019) was included as it assessed the direct effects of the programme on yield
		Moreover, Umar is a descriptive study, not an impact evaluation. The paper mentions randomisation and the use of propensity score matching; however, there is no evidence that these methods were used, and the results presented are descriptive
Dey et al. (2010); The impact of integrated aquaculture–agriculture on small‐scale farms in Southern Malawi	Akuffo and Quagrainie (2019); Assessment of household food security in fish farming communities in Ghana	Both of these papers use propensity score matching to analyse the effects of aquaculture on different outcomes. Dey et al. (2010), was included as it analyses the effects of long‐term dissemination activities by WorldFish promoting integrated aquaculture‐agriculture (IAA) practices. While Akuffo and Quagrainie (2019) was excluded as it did not focus on a specific intervention. The authors had a binary variable indicating treatment which equalled one when a household had taken part in fish‐farming. Although different programmes, which had been active in Ghana were mentioned, the analysis did not uniquely or directly assess the effects of a certain programme and so was excluded
Cahyadi and Bahramalian (2019); Sustainable Market Access through Responsible Trade of Fish (SMART‐Fish Indonesia)—Impact Evaluation Report	Dickson et al. (2016); Increasing fish farm profitability through aquaculture best management practice training in Egypt	Both papers focused on field‐based training for farmers. In the case of Cahyadi and Bahramalian (2019) this was for the introduction of new technology, whereas in the case of Dickson et al. (2016), it was for best management practices. The reason Dickson was excluded was due to the fact that although the intervention and outcomes were of relevance, other authors did not address the issue of selection into the programme. Programme nonparticipants were selected randomly from a database, but as participation in the programme was dependent on a farmer knowing the trainer, without carrying out an analysis beyond comparison of means, the issue of baseline differences between intervention and control farmers was not accounted for. Like most papers in our review, Cahyadi and Bahramalian used propensity score matching to overcome this issue
Amankwah et al. (2018); Impact of aquaculture feed technology on fish income and poverty in Kenya	Duy and Flaaten (2016); Profitability effects and fishery subsidies: average treatment effects based on propensity scores	Each of these papers assessed government programmes providing technology and subsidies. Each paper also used propensity score matching as a method to successfully create a counterfactual for analysis. Whereas Amankwah et al. (2018) was focused on outcomes for fish farmers and aquaculture, Duy and Flaaten (2016) focused on the effects of subsidies on the profitability of fisheries. The focus of their analysis was not on aquaculture as defined in our review, rather it was on fisheries and fishing, thus showing the distinction we employed in the selection of studies

### A.5. Data extraction tools

The data extraction process was conducted in two stages. The first focused on characteristics of included studies and the programme reported, and its presented in Table A7. The second stage was focused on extracting information of the study designs and the quantitative results reported in the papers, which is shown in Table A8.

**Table A7:** Data extraction tool for study characteristics

Section	Code	Description
Report identification	Study ID	Use EPPI's internal item ID (8 digits code)
	Author(s)	For 1 author: leading author last name (e.g., Gomez)
		For 2 authors: both author last names with ampersand in between (e.g., Smith & Bahn)
		For 3 or more authors: leading author last name followed by et al. (e.g., Gupta et al.)
	Title of report	Use only the English version of the publication's main title
	Publication date	Year the study was published. If unsure, look for the study in EPPI and copy the year shown in its record
	URL	Add URL or DOI of the landing page of the study (preferable) or the URL of the full‐text PDF
	Publication type	What is the impact evaluation publication type?
		1 = Peer‐reviewed journal article
		2 = Book chapter/whole book
		3 = Conference paper
		4 = Institutional report
		5 = Working paper
		6 = Implementation document
		7 = PhD thesis/dissertation
	Language of publication	Language of publication of the impact evaluation, for example, English, Spanish, French, and so forth
	Report funding agency	Select the category of the agency/agencies funding the evaluation/study
		1 = Government
		2 = NGO, Foundation or Charity
		3 = Multilateral/bilateral organisation (World Bank, UN)
		4 = Private sector
		5 = University or research centre
		6 = Other
		8 = Not clear
	Report funding agency name	Add the name of the agency/agencies funding the evaluation/study. Add the name for each row in the previous question
	Conflict of interest	Is there a potential conflict of interest associated with the study which could influence the results collected/reported? (e.g., Is there a declaration of conflict of interest? Is any of the authors related in any way to the funding or implementing institution?)
		0 = No
		1 = Yes
		8 = Not clear
	Conflict of interest description	If Yes in previous question, please add reason for your answer to whether there is a conflict of interest. Add page numbers as relevant
	Other methods	If the impact evaluation addresses other questions than effectiveness, note research questions and methods used, adding page numbers as relevant
	Other papers used for coding	Author (Publication date) followed by the type of additional paper used for coding (descriptive quantitative, qualitative, process evaluations, costs, etc.), NA if not used. For example, Gomez (1999) process evaluation
Intervention context	Project name	State the programme, project, or policy name being evaluated. If there is no name, then list the location
	Continent name	Select the continent/region in which the study was conducted
		1 = East Asia and Pacific
		2 = Europe and Central Asia
		3 = Latin America and Caribbean
		4 = Middle East and North Africa
		5 = North America
		6 = South Asia
		7 = Sub‐Saharan Africa
	Country	Select the countries in which the study was conducted
	Region	If provided, give detailed information on the administrative division of a country where the study took place, for example regions/districts covered. This includes both intervention and control groups
	World bank income category	Select the World Bank income classification of the country at the time when the intervention started. For example, if an intervention was implemented in 2012, use the classification for the country in 2012. If there is no implementation date, use the publication year
		Add one income category for each country
		1 = Low income country
		2 = Lower‐middle income country
		3 = Upper‐middle income country
	Exposure to climate shocks	Select the Exposure classification of the country at the time when the intervention started, according to the World Risk Index. Note that the Exposure classification is one of the dimensions of the World Risk Index
		Add one Exposure classification for each country
		1 = Very high
		2 = High
		3 = Medium
		4 = Low
		5 = Very low
Intervention description	Intervention type	Write a short paragraph to describe, in the author's own words, the intervention type and characteristics. The description should be as detailed as possible. Add page numbers for each relevant description
	Objectives of intervention	Note the objectives of the intervention as stated in the study or other relevant documents
	Programme theory	Do the authors make explicit reference to the programme theory, theory of change, conceptual framework, or similar?
		0 = No
		1 = Yes
		8 = Not clear
	Programme theory description	If Yes in previous question, report any description/statement of programme theory as stated by the author(s). Add page numbers as relevant
	Programme theory evaluation	Is the study using theory to inform the evaluation design and analysis? Describe if and how the authors use theory in the evaluation; for example, do they use it to inform data collection (e.g., purposely collecting specific data to be able to address their research questions)?
	Intervention components	Based on the intervention type, objectives and theory of change, select the relevant components that would describe the focus of the intervention
		Select as many codes as relevant.
		1 = Productivity
		2 = Income
		3 = Nutrition and health
		4 = Women's empowerment
		5 = Other (specify)
	Intervention components other	If Other in previous question, provide, in the authors' own words, the main focus of the intervention. Add page numbers as relevant
	Intervention implementing agency	Select the category of the agency/agencies implementing the intervention. Use one code for each organisation and select as many codes as relevant
		1 = Government
		2 = NGO, Foundation or Charity
		3 = Multilateral/bilateral organisation (World Bank, UN)
		4 = Private sector
		5 = University or research centre
		6 = Other
		8 = Not clear
	Iintervention implementing agency name	Add the name (and department if relevant) of the agency/agencies implementing the intervention. Add page numbers as relevant
	Intervention funding agency	Select the category of the agency/agencies funding the intervention
		Note that this may not be the same as the organisation funding the evaluation of the intervention. Use one code for each organisation and select as many codes as relevant
		1 = Government
		2 = NGO, Foundation or Charity
		3 = Multilateral/bilateral organisation (World Bank, UN)
		4 = Private sector
		5 = University or research centre
		6 = Other
		8 = Not clear
	Intervention funding agency name	Add name of the agency/agencies funding the intervention. Add page numbers as relevant
	Intervention development	What information do the impact evaluation and additional papers present about how the intervention was designed/created? For example: Who designed the intervention? What process was used to develop the design? Did the intervention respond to a local demand, or was it a donor‐created initiative? Was this intervention an initiative developed in consultation with field partners? What process was used to develop the design?
		Please add any information in the paper(s) with the relevant page numbers, or N/A if no information is presented
	Intervention start	State date when the intervention implementation started (month/year is preferred (Dec 2020), but year will also suffice). If not stated, leave blank
	Intervention end	State date when the intervention implementation ended (month/year is preferred (Dec 2020), but year will also suffice). If not stated, leave blank)
	Intervention length	State the intervention length in number of months. If there is no month information, consider the whole year (12 months) for this estimation
Intervention target	Scale of intervention	At what scale was the intervention implemented?
		Select Local is the study takes place within one region in a country; Subnational if the study takes place across one or more regions of a country; National if the study takes place at the national level, or if the authors state to be evaluating a national programme; or Transnational if the study takes place across multiple countries
		1 = Local
		2 = Subnational
		3 = National
		4 = Transnational
		8 = Not clear
	Scale of operation	Is the intervention focused on a specific scale of aquaculture operation or production?
		Because definitions based on the scale of the operations are not agreed upon and may have different meanings in different countries and regional contexts (Philips et al., 2016) describe, in the author's own words, the scale of aquaculture operation
	Area	Is the intervention focused on a specific area? Code as explicitly stated in the study and please do not make assumptions of where it might take place
		1 = Urban
		2 = Rural
		3 = Both
		8 = Not clear
	Value chain	Where in the aquaculture value chain is the intervention focused on? Value chain is defined as the full range of activities that are required to bring a product or service from conception, through production and transformation, to delivery to final consumers, and final disposal after use (Kaplinsky & Morris, 2000). Select as many codes as relevant
		1 = Supply and services before production (e.g., supply of inputs such as feed and fertilisers)
		2 = Production (including use of technologies and management practices)
		3 = Processing
		4 = Trading
		5 = Marketing
		6 = Supply and services after production (e.g., disposal)
		7 = Other
		8 = Not clear
	Value chain other	If Other in previous question, specify stage of aquaculture value chain where the intervention is focused on. Add number pages as relevant
	Land use	Where is the aquaculture intervention practised in?
		Code as explicitly stated in the study. Inland examples include when aquaculture is practised in ponds, tanks, rice‐fields or other facilities built on dry land. Water examples include when aquaculture is practised in coastal or offshore water or inland waters such as lakes, reservoirs, or rivers
		1 = Inland
		2 = Water
		3 = Both
		8 = Not clear
	Land ownership	If Inland in previous question, do individuals engaging in aquaculture own the land where this activity is located?
		Code as explicitly stated in the study
		0 = No
		1 = Yes
		8 = Not clear
		NA if not inland
	Land ownership 1	If No in previous question, who owns the land where the aquaculture activity is located?
		Code as explicitly stated in the study
		1 = Private entity (e.g., company or corporation) or individual
		2 = Community or shared ownership between individuals
		3 = Public entity (e.g., local authority)
		4 = Other
		8 = Not clear
		NA if yes in previous question
	Land ownership 2	If Other in previous question, specify who owns the land where the aquaculture activity is located. Add number pages as relevant
	Aquaculture system	Where does the aquaculture activity take place?
		Code as explicitly stated in the study. Select as many codes as relevant
		1 = Ponds
		2 = Cages
		3 = Tanks
		4 = Rice fields (rice‐fish integrated systems)
		5 = Other
		8 = Not clear
	Aquaculture system other	If Other in previous question, specify the aquaculture system where the intervention takes place. Add number pages as relevant
	Products	Which aquaculture products is the intervention focused on? Code as explicitly stated in the study. Specific product types are preferred (e.g., oysters, mussels, shrimps, crayfish, seaweed) but if the study does not specify this information, use general codes (e.g., fish, molluscs, etc.). If there is more than one product, add each in a new row. Add number pages as relevant
	Sex	Is the intervention focused specifically on female or male individuals? Code as explicitly stated in the study
		1 = Women
		2 = Men
		3 = Both
		8 = Not clear
	Age	Is the intervention focused on individuals from a certain age‐range? Code as explicitly stated in the study. While the study may measure outcomes for other groups, this code should reflect the age of the group targeted by the intervention
		For example, an intervention focused on women could also measure outcomes on production or on children's nutrition. In this case, code the specific age range of women (if available, for example, 18‐50 years old) or code as “adult”
		Add page numbers as relevant
	Socioeconomic background	From which socioeconomic status (SES) are individuals targeted by the intervention? Code as explicitly stated in the study and please do not make assumptions about it. Select as many codes as relevant
		1 = Low SES
		2 = Medium SES
		3 = High SES
		8 = Not clear
	Educational background	What is the (general) level of education of individuals targeted by the intervention? Code as explicitly stated in the study and please do not make assumptions about it. Select as many codes as relevant
		1 = No formal education
		2 = Primary education
		3 = Secondary education
		4 = Tertiary education
		8 = Not clear
Implementation information	Programme take‐up	Is there any information about programme take‐up? This is, of those individuals the interventions was targeting, is there information on how many ended up participating in the programme or intervention?
		1 = Yes, commentary from author
		2 = Yes, formally assessed
		3 = No
	Methods of assessing take‐up	If formally assessed, which methods are used to measure programme take‐up?
		1 = Observation by intervention staff
		2 = Reporting by participants
		3 = Other
		9 = NA
	Take‐up results	What is the result or information provided of the programme take‐up?
		Add page number of relevant information
	Programme adherence	Is there any information about programme adherence? This is, among those who accessed the intervention, does the study assess how many beneficiaries did take part in the intervention?
		1 = Yes, commentary from author
		2 = Yes, formally assessed
		3 = No
	Methods of assessing adherence	If formally assessed, which methods are used to measure programme adherence?
		1 = Observation by intervention staff
		2 = Reporting by participants
		3 = Other
		9 = NA
	Adherence results	What is the result or information provided of the programme adherence?
		Add page number of relevant information
	Fidelity implementation quality	Is there any information on implementation fidelity/intervention delivery quality? This is, any information on how close the intervention design was to the intervention implementation
		1 = Yes, commentary from author
		2 = Yes, formally assessed
		3 = No
	Methods of assessing fidelity	If formally assessed, which methods are used to measure implementation fidelity/intervention delivery quality
		1 = Observation by intervention staff
		2 = Reporting by participants
		3 = Other
		9 = NA
	Fidelity results	What is the result or information provided of the implementation fidelity/intervention delivery quality
		Add page number of relevant information
	Barriers/facilitators	Is there any information on barriers or facilitating factors that could affect the effectiveness of the intervention? This is, any factor that helped or hindered the implementation or general process of the intervention (e.g., an unforeseen event that affected the implementation in all or some areas; the parallel implementation of other interventions in the same area/at the same time; etc.)
		Add page number of relevant information
	Other implementation info	Any other description of process/implementation factors not covered above?
		Add page number of relevant information
Cost data	Cost data identification	Are any cost data estimates provided in the report or other documents?
		0 = No
		1 = Yes
	Cost data description	Summarise the type of data available (we will then assess if/how to analyse it). Report any details of unit costs and/or total costs, year and currency used, and page numbers

**Table A8:** Data extraction tool for quantitative data

Section	Code	Description
Study design	Study ID	This is the study ID—it should match the EPPI ID (e.g., 58374737)
	Estimate ID	The estimate ID will provide a specific number for each effect size extracted and should include the original study number, underscore, then the unique ID number (e.g., 58374737_1, 58374737_2 and so on)
	Author	For 1 author: leading author last name (e.g., Gomez)
		For 2 authors: both author last names with ampersand in between (e.g., Smith & Bahn)
		For 3 or more authors: leading author last name followed by et al. (e.g., Gupta et al.)
	Year	Year published
	Design	0 = Experimental Design (e.g., RCT),
		1 = Quasi‐Experimental Design
	How counterfactual is chosen	Free text (e.g., random control trial, propensity score matching, etc.)—Multiple codes are ok
	Analysis type for this effect size	Free text, what type of analysis was used (Regression, 2SLS, ANCOVA, etc.)—Multiple codes are ok
	Estimate type	Type of data for this effect size:
		1 = Continuous—means and SDs
		2 = Continuous—mean difference and SD
		3 = Dichotomous outcome—proportions
		4 = Regression data—dichotomous outcome (e.g., logistic regression)
		5 = Regression data—continuous outcome (e.g., linear regression)
	Comparison	1 = No intervention (service delivery as usual)
		2 = Other intervention
		3 = Pipeline (wait‐list) control (still service delivery as usual)
	Describe comparison group	If answer above is (1) no intervention, type N/A, if (2) Other Intervention, list what intervention the control group is receiving, if (3) Pipeline control, report when the control group will receive the intervention in relation to the treatment group (e.g., one year later)
	Subgroup	Is this analysis of a subgroup?
		0 = No
		1 = Yes
	If yes to subgroup, describe	Free text, describe the subgroup if applicable (e.g., boys, girls). If no subgroup, type N/A
	Source	Note the page number, table number, column, and row you used to extract the data
	Treatment Effect	1 = Intention to Treat (ITT)
		2 = Average Treatment Effect on the Treated (ATET)
		3 = Average Treatment Effect (ATE)
		4 = Local Average Treatment Effect (LATE)
	Intervention	Free text, what is the author description of the intervention?
	Exposure to intervention (in months)	How long is the intervention exposure itself?
	Evaluation period (in months)	The total number of months elapsed between the end of an intervention and the point at which an outcome measure is taken post intervention, or as a follow‐up measurement. If <1 month, use decimals (e.g., one week would be.25)
	Postintervention or change from baseline?	0 = Postintervention
		1 = Change from baseline
	data set	Record if data comes from an identified data set (free text)
	Author definition of outcome	Free text—How does the author define the outcome?
Outcome Codes	Production, Productivity	Code 1 under any applicable columns
	Income	Code 1 under any applicable columns
	Nutrition	Code 1 under any applicable columns
	Women's empowerment	Code 1 under any applicable columns
Effect Size Data Extraction	Reverse Sign (i.e., decrease is good)	Record 0 = ”no” if an increase is good, record 1 = ”yes” if a decrease is good and the sign needs to be reversed
	Unit of analysis	What is the unit of analysis? UOA for this effect size:
		1 = Individual
		2 = Household
		3 = Group (e.g., farm, community organisation)
		4 = Village
		5 = Other
		6 = Not clear
	mean_t	Outcome mean for the treatment group
	sd_t	Outcome standard deviation for treatment group
	mean_c	Outcome mean for the comparison group
	sd_c	Outcome standard deviation for control group
	mean_overall_diff	Overall mean difference (treatment—control)
	diff se	Standard error of the overall mean difference
	Diff _t	*t* statistic of mean difference
	Odds ratio	Odds ratio reported in the study
	OR_se	Odds ratio standard error reported in the study
	Risk ratio	Risk ratio reported in study
	RR_se	Risk ratio standard error
	reg_coeff	Report the regression coefficient of the treatment effect
	reg_SE	Report the associated standard error of the regression coefficient.
	reg_t	Report the associated t statistic of the effect size (coefficient/SE)
	P value	Exact p value if given, if not, record as written in the manuscript (e.g., *p* < .001, or *p* > .05)
	clust_t	Number of clusters—treatment group
	clust_c	Number of clusters—control group
	clust_T	Number of clusters—total sample
	n_t	Sample size—treatment group
	n_c	Sample size—control group
	n_T	Sample size—total sample
	periods (1 if cross sectional)	Record how many periods of evaluation there are (e.g., cross section is 1, panel data with 3 measurements is 3)
	Treatment Variable	Record the treatment variable as written in the model (e.g., the variable name the author uses, such as ("Intervention x Time")

### A.6. Risk of bias tool

The critical appraisal of included studies was conducted using the criteria presented in Table A9, A11 for quasi‐experimental designs, and Table A10 for RCTs. Each table presents the risk of bias domains, criteria, and coding used for the assessment of each study.

**Table A9:** Risk of bias assessment tool for quasi‐experimental designs

Question	Criteria	Coding
Study ID		Open answer
Study first author		Open answer
Outcome		Open answer
Study design: What type of study design is used?		1 = Natural experiment: randomised or as‐if randomised 2 = Natural experiment: regression discontinuity (RD) 3 = CBA (nonrandomised assignment with treatment and contemporaneous comparison group, baseline and end line data collection)—individual repeated measurement 4 = CBA pseudo panel (repeated measurement for groups but different individuals) 5 = Interrupted time series (with or without contemporaneous control group) 6 = Panel data, but no baseline (pre‐test) 7 = Comparison group with end line data only
Methods used for analysis: Which methods are used to control for selection bias and confounding?		1 = Statistical matching (PSM, CEM, covariate matching) 2 = Difference in differences (DID) estimation methods 3 = IV‐regression (2‐stage least squares or bivariate probit) 4 = Heckman selection model 5 = Fixed effects regression 6 = Covariate adjusted estimation 7 = Propensity weighted regression 8 = Comparison of means 9 = Other (please state)
Study population	Provide any details in the paper that describe how the study population was selected, covering: a) How is the population selected? what is the sampling strategy to recruit participants from that population into the study? b) What are the characteristics of that study participants? c) Was this a pilot program aimed at being scaled up? d) Were there specific factors of success or failure in the implementation?	Open answer
Ethical clearance	Provide any details of ethical research clearances granted. Report unclear if this information is not available	Open answer
Study registration	Provide any details of study registration, including registry IDs, and so forth	Open answer
1: Selection bias Mechanism of assignment: was the allocation or identification mechanism able to control for selection bias?	For regression discontinuity designs: a) Allocation is made based on a predetermined discontinuity on a continuous variable (regression discontinuity design) and blinded to participants or; b) If not blinded, individuals reasonably cannot affect the assignment variable in response to knowledge of the participation decision rule; c) and the sample size immediately at both sides of the cut‐off point is sufficiently large to equate groups on average	1 = Yes, 2 = Probably Yes, 3 = Probably No, 4 = No, 8 = Unclear
	For assignment based nonrandomised programme placement and self‐selection (studies using a matching strategy or regression analysis, excluding IV): a) Participants and nonparticipants are either matched based on all relevant characteristics explaining participation and outcomes, or; b) all relevant characteristics are accounted for. c) and the data set used contains relevant variable that are measured in a relevant way (i.e. they were not collected for a different purpose initially and therefore are good proxy for some characteristics).	
	For identification based on an instrumental variable (IV estimation): Justification for coding decision, include a brief summary of justification for rating, cite relevant pages.	
2: Confounding Group equivalence: was the method of analysis executed adequately to ensure comparability of groups throughout the study and prevent confounding?	For regression discontinuity design: a) The interval for selection of treatment and control group is reasonably small OR authors have weighted the matches on their distance to the cut‐off point; b) and the mean of the covariates of the individuals immediately at both sides of the cut‐off point (selected sample of participants and nonparticipants) are overall not statistically different based on t‐test or ANOVA for equality of means; c) Significant differences in covariates of the individuals have been controlled in multivariate analysis; and for cluster‐assignment, authors control for external cluster‐level factors that might confound the impact of the programme	1 = Yes, 2 = Probably Yes, 3 = Probably No, 4 = No, 8 = Unclear
	For nonrandomised trials using difference‐in‐differences methods of analysis: a) The authors use a difference‐in‐differences (or fixed effects) multivariate estimation method; b) the authors control for a comprehensive set of individual time‐varying characteristics, and for cluster‐assignment, authors control for external cluster‐level factors that might confound the impact of the programme; c) and the attrition rate is sufficiently low and similar in treatment and control, or the study assesses that drop‐outs are random draws from the sample (for example, by examining correlation with determinants of outcomes, in both treatment and comparison groups)	
	For statistical matching studies including propensity scores (PSM) and covariate matching: a) Matching is either on baseline characteristics or time‐invariant characteristics which cannot be affected by participation in the programme; and the variables used to match are relevant (for example, demographic and socio‐economic factors) to explain both participation and the outcome (so that there can be no evident differences across groups in variables that might explain outcomes); and, for cluster‐assignment, authors control for external cluster‐level factors that might confound the impact of the programme b) In addition, for PSM Rosenbaum's test suggests the results are not sensitive to the existence of hidden bias; c) And, with the exception of Kernel matching, the means of the individual covariates are equated for treatment and comparison groups after matching; d) different matching methods including varying sample sizes yields the same results and authors take into account the use of control observations multiple times against the same treatment in their standard error calculation.	
	For regression‐based studies using cross sectional data (excluding IV): a) The study controls for relevant confounders that may be correlated with both participation and explain outcomes (for example, demographic and socioeconomic factors at individual and community level) using multivariate methods with appropriate proxies for unobservable covariates, and, for cluster‐assignment, authors control particularly for external cluster‐level factors that might confound the impact of the programme; b) and a Hausman test with an appropriate instrument suggests there is no evidence of endogeneity; c) and none of the covariate controls can be affected by participation; d) and either, only those observations in the region of common support for participants and nonparticipants in terms of covariates are used, or the distributions of covariates are balanced for the entire sample population across groups.	
	For identification based on an instrumental variable (IV estimation): a) The instrumenting equation is significant at the level of *F* ≥ 10 (or if an *F* test is not reported, the authors report and assess whether the R‐squared (goodness of fit) of the participation equation is sufficient for appropriate identification); b) the identifying instruments are individually significant (*p* ≤ .01); for Heckman models, the identifiers are reported and significant (*p* ≤ .05); c) where at least two instruments are used, the authors report on an over‐identifying test (*p* ≤ .05 is required to reject the null hypothesis); and none of the covariate controls can be affected by participation and the study convincingly assesses qualitatively why the instrument only affects the outcome via participation. If the instrument is the random assignment of the treatment, the reviewer should also assess the quality and success of the randomisation procedure in part a). d) and, for cluster‐assignment, authors particularly control for external cluster‐level factors that might confound the impact of the programme (e.g., weather, infrastructure, community fixed effects, and so forth) through multivariate analysis.	
3: Performance bias Was the process of being observed free from motivation bias?	a) For data collected in the context of a particular intervention trial (randomised or nonrandomised assignment), the authors state explicitly that the process of monitoring the intervention and outcome measurement is blinded, or argue convincingly why it is not likely that being monitored could affect the performance of participants in treatment and comparison groups in different ways (such as resulting in Hawthorne or John Henry effects). b) The study is based on data collected in the context of a survey, and not associated with a particular intervention trial, or data are collected from administrative records or in the context of a retrospective (ex post) evaluation	1 = Yes, 2 = Probably Yes, 3 = Probably No, 4 = No, 8 = Unclear
4: Spill‐overs, cross‐overs and contamination Was the study adequately protected against spill‐overs, cross‐overs and contamination?	a) There was no implementation issues that might have led the control participants to receive the treatment (implementer's mistake). b) The intervention is unlikely to spill‐over to comparisons (e.g. participants and nonparticipants are geographically and/or socially separated from one another and general equilibrium effects are not likely) or the potential effects of spill overs were measured (e.g. variation in the % of unit within a cluster receiving the treatment). c) There is no risk of contamination by external programs: the treatment and comparisons are isolated from other interventions which might explain changes in outcomes. d) There is nothing in the surveys that might have given the control participants an idea of what the other group might receive OR they did but there is no risk that this has changed their behaviours; AND the survey process did not reveal information to the control group that they did not have before (e.g., the study aims to measure increase in take up of a service or product that participants might not know about). Authors might put something in place in the design of the study that allows to control for that survey effect (e.g., a pure control with no monitoring except baseline end line)	1 = Yes, 2 = Probably Yes, 3 = Probably No, 4 = No, 8 = Unclear
5: Outcome measurement bias	a) Outcome assessors are blinded or the outcome measures are not likely to be biased by their judgement. b) For self‐reported outcomes: respondents in the intervention group are not more likely to have accurate answers due to recall bias; c) For self‐reported outcomes: respondents do not have incentives to over/under report something related to their performance or actions, OR researchers put in place mechanisms to reduce the risk of reporting bias (researchers not strongly involved in the implementation of the program and it is clear that their answers to the survey will not affect what they receive in the future) OR authors have measured the risks of bias through falsification tests or measuring the effect on placebo outcomes in cases where there was a risk of reporting bias. d) Timing issue: the data collection period did not differ between intervention and comparison group, the baseline data is not likely to be affected by the beginning of the intervention or affects a small percentage of the study participants	1 = Yes, 2 = Probably Yes, 3 = Probably No, 4 = No, 8 = Unclear
6: Reporting bias Selective analysis reporting: was the study free from selective analysis reporting?	a) A preanalysis plan is published, especially for prospective NRS but it should also be for retrospective studies b) Authors use “common” methods of estimation (i.e., credible analysis method to deal with attribution given the data available); c) There is no evidence that outcomes were selectively reported (e.g. results for all relevant outcomes in the methods section are reported in the results section); d) Requirements for specific methods of analysis: ‐ For PSM and covariate matching: (a) Where over 10% of participants fail to be matched, sensitivity analysis is used to re‐estimate results using different matching methods (Kernel Matching techniques); (b) For matching with replacement, no single observation in the control group is matched with a large number of observations in the treatment group. ‐ For IV (including Heckman) models, (a) The authors test and report the results of a Hausman test for exogeneity (*p* ≤ .05 is required to reject the null hypothesis of exogeneity); (b) the coefficient of the selectivity correction term (Rho) is significantly different from zero (*p* < .05) (Heckman approach). ‐ For studies using multivariate regression analysis, authors conduct appropriate specification tests (e.g., testing robustness of results to the inclusion of additional variables, or (very rare) reporting results of multicollinearity test, etc.)	1 = Yes, 2 = Probably Yes, 3 = Probably No, 4 = No, 8 = Unclear
7: Other risks of bias Is the study free from other sources of bias?	Justification for coding decision. Include a brief summary of justification for rating, cite relevant pages	1 = Yes, 4 = No
8: External validity	What do authors say about external validity, if anything?	Open answer
9: Random Sampling Was random sampling used?	Describe sampling process	1 = Yes, 4 = No

**Table A10:** Risk of bias assessment tool for randomised controlled trials

Question	Criteria	Coding
Study ID		Open answer
Study first author		Open answer
Design type: What type of study design is used?		1 = Randomised controlled trial (RCT) (random assignment to households/individuals) or quasi‐RCT 2 = Cluster‐RCT (quasi‐RCT)
Methods used for analysis: Which methods are used to control for selection bias and confounding?		1 = Statistical matching (PSM, CEM, covariate matching) 2 = Difference in differences (DID) estimation methods 3 = IV‐regression (2‐stage least squares or bivariate probit) 4 = Heckman selection model 5 = Fixed effects regression 6 = Covariate adjusted estimation 7 = Propensity weighted regression 8 = Comparison of means 9 = Other (please state)
Design and analysis method description	Briefly describe the study design and analysis method undertaken by the authors	Open answer
Study population	Provide any details in the paper that describe how the study population was selected, covering: a) How is the population selected? what is the sampling strategy to recruit participants from that population into the study? b) What are the characteristics of that study participants? c) Was this a pilot programme aimed at being scaled up? d) Were there specific factors of success or failure in the implementation?	Open answer
Type of comparison group	Indicate type of comparison group	1 = No intervention (service delivery as usual) 2 = Other intervention 3 = Pipeline (wait‐list) control (still service delivery as usual)
Type of comparison group (if other)		Open answer
Ethical clearance	Provide any details of ethical research clearances granted. Report unclear if this information is not available	Open answer
Study registration	Provide any details of study registration, including registry IDs, and so forth	Open answer
1: Assignment mechanism Was the allocation or identification mechanism random or as good as random?	a) The authors describe a random component in sequence generation/randomisation method (e.g., lottery, coin toss, random number generator) and assignment is performed for all units at the start of the study centrally or using a method concealed from participants and intervention delivery b) If public lottery is used for the sequence generation, authors provide detail on the exact settings and participants attending the lottery c) If a special randomization procedure is used to ensure balance, it is well described and justified given the study setting (stratification, pairwise matching, unique random draw, multiple random draws, etc.) d) A balance table is reported suggesting that allocation was random between all groups including subgroup receiving different treatment within control or treatment groups (if the comparison is relevant for this assessment)	1 = Yes, 2 = Probably Yes, 3 = Probably No, 4 = No, 8 = Unclear
2: Unit of analysis Is unit of analysis in cluster allocation addressed in standard error calculation?	Method used to address differences between UoA and unit of data collection	1 = Yes 2 = No 3 = Not reported/unclear 4 = Not applicable
3. Selection bias Was any differential selection into or out of the study (attrition bias) adequately resolved?	Justification for coding decision. Include a brief summary of justification for rating, cite relevant pages	1 = Yes 2 = Probably Yes 3 = Probably No 4 = No 8 = Unclear
4: Confounding and group equivalence Was the method of analysis executed adequately to ensure comparability of groups throughout the study and prevent confounding	a) Baseline characteristics are similar in magnitude; b) Unbalanced covariates at the individual and cluster level are controlled in adjusted analysis; c) Adjustments to the randomization were taken into account in the analysis (stratum fixed effects, pairwise matching variables)? (Bruhn & McKenzie 2009)	1 = Yes 2 = Probably Yes 3 = Probably No 4 = No 8 = Unclear
5. Deviations from intended interventions Spill‐overs, cross‐overs and contamination: was the study adequately protected against spill‐overs, cross‐overs and contamination?	a) There was no implementation issues that might have led the control participants to receive the treatment (implementer's mistake) b) The intervention is unlikely to spill‐over to comparisons (e.g., participants and nonparticipants are geographically and/or socially separated from one another and general equilibrium effects are not likely) or the potential effects of spill overs were measured (e.g., variation in the % of unit within a cluster receiving the treatment) c) There is no risk of contamination by external programmes: the treatment and comparisons are isolated from other interventions which might explain changes in outcomes d) There is nothing in the surveys that might have given the control participants an idea of what the other group might receive OR they did but there is no risk that this has changed their behaviours; AND the survey process did not reveal information to the control group that they did not have before (e.g., the study aims to measure increase in take up of a service or product that participants might not know about). Authors might put something in place in the design of the study that allows to control for that survey effect (e.g., a pure control with no monitoring except baseline end line)	1 = Yes 2 = Probably Yes 3 = Probably No 4 = No 8 = Unclear
6. Performance bias Was the process of monitoring individuals unlikely to introduce motivation bias among participants?	a) The authors state explicitly that the process of monitoring the intervention and outcome measurement is blinded and conducted in the same frequency for treatment and control groups, or argue convincingly why it is not likely that being monitored could affect the performance of participants in treatment and comparison groups in different ways (such as resulting in Hawthorne or John Henry effects) b) The outcome is based on data collected in the context of a survey, and not associated with a particular intervention trial, or data are collected from administrative records or in the context of a retrospective (ex post) evaluation	1 = Yes, 2 = Probably Yes, 3 = Probably No, 4 = No, 8 = Unclear
7. Outcome measurement bias Was the study free from biases in outcome measurement?	a) Outcome assessors are blinded or the outcome measures are not likely to be biased by their judgement b) For self‐reported outcomes: respondents in the intervention group are not more likely to have accurate answers due to recall bias c) For self‐reported outcomes: respondents do not have incentives to over/under report something related to their performance or actions, OR researchers put in place mechanisms to reduce the risk of reporting bias (researchers not strongly involved in the implementation of the programme and it is clear that their answers to the survey will not affect what they receive in the future) OR authors have measured the risks of bias through falsification tests or measuring the effect on placebo outcomes in cases where there was a risk of reporting bias d) Timing issue: the data collection period did not differ between intervention and comparison group, the baseline data is not likely to be affected by the beginning of the intervention or affects a small percentage of the study participants	1 = Yes 2 = Probably Yes 3 = Probably No 4 = No 8 = Unclear
8. Reporting bias Analysis reporting: Was the study free from selective analysis reporting?	a) A preanalysis plan or trial protocol is published and referred to or the trial was preregistered or the outcomes were preregistered b) Authors report results corresponding to the outcomes announced in the method section (there is no outcome reporting bias) c) Authors report results of unadjusted analysis and intention to treat (ITT) estimation, alongside any adjusted and treatment‐on‐the‐treated/complier‐average‐causal‐effects analysis) d) Authors use the appropriate analysis method (use baseline data when available) and different treatment arms are differentiated in the analysis e) Authors have reported all the analysis which could help understand the results and no other bias is assessed as unclear due to the lack of an important analysis (e.g., a balance table or a subgroup analysis)	1 = Yes 2 = Probably Yes 3 = Probably No 4 = No 8 = Unclear
9. Other risks of bias Is the study free from other sources of bias?	Justification for coding decision. Include a brief summary of justification for rating, cite relevant pages. For example, information is collected using a different survey instrument in different intervention groups; measurement of the intervention received in unclear	1 = Yes 4 = No
10. Blinding—observers Blinding of participants?	If there is no information, code NO. If there is information but it is ambiguous, code UNCLEAR	1 = Yes 2 = No 8 = Unclear 9 = N/A
11. Blinding—observers Blinding of outcome assessors?	If there is no information, code NO. If there is information but it is ambiguous, code UNCLEAR	1 = Yes 2 = No 8 = Unclear 9 = N/A
12. Blinding—analysts Blinding of data analysts?	If there is no information, code NO. If there is information but it is ambiguous, code UNCLEAR	1 = Yes 2 = No 8 = Unclear 9 = N/A
13. Blinding—method(s) Method(s) used to blind	Describe method(s) used to blind	Open answer (including describe method of placebo control); 9 = N/A
14. External validity	a) What do authors say about external validity?	Open answer—Include all information that can help assess the external validity of the results

### A.7. Additional search of relevant papers of included studies

In addition to our main search, we conducted a second search for any additional documentation we could find on our 13 included programmes in relation to barriers and facilitators of programme effectiveness (RQ4) and the cost effectiveness of the programmes (RQ5). We conducted this search by attempting to locate documents related to our programmes on Google, as well as in institutional websites when the implementing organisation was clear. Table A9, A11 shows the new sources identified for each programme, for both the barriers and facilitators analysis and the cost‐effectiveness analysis.

**Table A11:** Summary of additional sources used per programme

Programme name and country	Sources additional documentation	Sources cost data
Adivasi Fisheries Project (AFP) Bangladesh	Pant et al. (2014); WorldFish (2009)	Pant et al. (2014); WorldFish (2009)
NGO Banchte Shekha (BS) Bangladesh	Hallman et al. (2003)*; Kumar and Quisumbing (2011); Meizen‐Dick et al. (2003); Naved (2000); Bouis (2000)	No cost data identified
Community‐based Fish Culture in Seasonal Floodplains and Irrigation Systems (CBFC) Bangladesh	Sheriff et al. (2010)	Haque and Dey (2017)
Development of Sustainable Aquaculture Project (DSAP) Bangladesh	Mandal et al. (2004); Khondker and Pemsl (2011)*	Mandal et al. (2004)
Economic Stimulus Programme (ESP) Kenya	Kioi (2014); Njagi et al. (2013); Kiwiri and Njeru (2015)	Amankwah et al. (2018)
Fadama II Nigeria	Hima et al. (2016); Olaoye et al. (2011)	No cost data identified
Fadama III Nigeria	Hima et al. (2016); Fadare and Adereti (2017); Omobowale and Akinola (2017); Ovharhe (2020); Alawode and Oluwatayo (2019)*; Bature et al. (2013)	World Bank (2016)
Fish on Farms Project (FoF) Cambodia	Michaux et al. (2019)*; Moumin (2016); Talukder and Green (2014)*	No cost data identified
Greater Noakhali Aquaculture Extension Project (GNAEP) Bangladesh	FAO (2009); DANIDA (2008), Annex 4*; Bouis (2000)	DANIDA (2008), Annex 4
WorldFish Integrated Aquaculture‐Agriculture Dissemination (IAA) Malawi	Dey et al. (2010)*	No cost data identified
Mymensingh Aquaculture Extension Project (MAEP) Bangladesh	Hallman et al. (2003)*; Rand and Tarp (2009)*; Meizen‐Dick et al. (2003); DANIDA (2008), Annex 4*; Naved (2000)	DANIDA (2008), Annex 4
Sustainable Agriculture, Food Security and Linkages project (SAFAL) Bangladesh	Kessler et al. (2017)	Kuijpers (2020); Kessler et al. (2017)
SMART‐Fish Indonesia	UNIDO (2019)	UNIDO (2019)

*Note*: Papers denoted with *(*n* = 8) are included studies of our review, thus, not additional sources for these analyses.

### A.8. Critical appraisal of qualitative studies

Eight studies using qualitative or mixed methods techniques were included in the analysis of barriers and facilitating factors that may affect the implementation of included aquaculture programmes. One of these studies is an impact evaluation included in the review (Hallman et al., 2003), while the other seven studies were identified through the second search of documentation related to included programmes. Table A12 presents the appraisal tool used, including the topics (questions) assessed and their corresponding answer options reflecting three assessment levels. We conducted a double appraisal for each study, and then discussed and reconciled disagreements. Additionally, Table A13 presents the results of the appraisal for each of these eight studies.

**Table A12:** Critical appraisal tool for qualitative studies

Question	Answer options		
1. Is the research aim clearly stated?	Yes, a strong statement	Yes, but an unclear or weak statement	No
2. Is there a description of the context in which the study takes place?	Yes, a strong description	Yes, but a weak description	No
3. Is there a clear link to relevant literature?	Yes, a clear strong link	Yes, but it could be improved	No
4. Is there a clear link to theory?	Yes, a strong one	Yes, but a weak one	No
5. Is there a description of the sampling procedure?	Yes, a strong description	Yes, but a partial description	No
6. Is the sampling strategy appropriate for the aims of the research?	Yes, very appropriate	Yes, but not completely appropriate	No
7. Are sample characteristics sufficiently reported?	Yes, there's a sufficient description	There's some description, but not enough	No
8. Is it clear how data were collected?	Yes, data collection is clearly described	Yes, but more details are needed	No
9. Are the methods of data recording reported?	Yes, and reported well	Yes, but only briefly mentioned	No
10. Did the collection of the data address the research aims?	Yes, completely	Yes, but not entirely	No
11. Are the methods of analysis explicitly stated?	Yes, and stated well	Yes, but more detail is needed	No
12. Were there any inbuilt checks to assure the quality of the analysis?	Yes, there were clear, strong checks throughout	Yes, but they were weak checks	No
13. Was there reflection on bias and positionality?	Yes, a clear reflection with detail provided	Yes, but there's only a brief mention of this	No
14. Are there any details about the people who conducted the sampling, data collection, and analysis?	Yes, plenty	Yes, a little	No
15. Is there enough data to support the claims?	Yes, there's plenty of clear, contextualised, relevant data	Yes, there's some data, but it's weak	No
16. Are diverse viewpoints considered?	Yes, thoroughly	Yes, only occasionally	No
17. Is there evidence which addresses every research aim?	Yes, every aim has some related evidence	Yes, but some aims have distinctly less attending to them	No
18. Has the question been answered?	Yes, with a clear, logical, thorough answer	Yes, but not well	No
19. Are relevant literature, theory, or practice discussed in relation to the results?	Yes, and discussed well	Yes, but only briefly or generally discussed	No
20. Has there been any triangulation?	Yes, strong triangulation	Yes, weak triangulation	No
21. Are weaknesses considered?	Yes, major weaknesses considered and attended to	Yes, but weaknesses are mentioned without much discussion	No
22. Have ethical issues been taken into consideration?	Yes, beyond just the IRB and consent, if needed	Yes, but not in depth	No

**Table A13:** Results of the critical appraisal of studies using qualitative or mixed‐methods

	Hallman et al. (2003)	Hima et al. (2016)	Kessler et al. (2017)	Mandal et al. (2014)	Moumin (2016)	Naved (2000)	Omobowale and Akinola (2017)	UNIDO (2019)
Research aims	Yes, a strong statement	Yes, a strong statement	Yes, a strong statement	Yes, a strong statement	Yes, a strong statement	Yes, a strong statement	Yes, a strong statement	Yes, a strong statement
Description of the context	Yes, a strong description	Yes, a strong description	Yes, a strong description	Yes, a strong description	Yes, a strong description	Yes, a strong description	Yes, a strong description	Yes, a strong description
Link to literature	Yes, a clear strong link	Yes, but it could be improved	No	No	Yes, a clear strong link	Yes, a clear strong link	Yes, a clear strong link	Yes, but it could be improved
Link to theory	Yes, a strong one	No	Yes, a strong one	No	Yes, a strong one	No	Yes, a strong one	Yes, a strong one
Sampling procedure	Yes, but a partial description	No	Yes, but a partial description	Yes, but a partial description	Yes, a strong description	Yes, but a partial description	Yes, but a partial description	Yes, but a partial description
Sampling strategy	Yes, very appropriate	Yes, very appropriate	Yes, very appropriate	Yes, very appropriate	Yes, very appropriate	Yes, very appropriate	Yes, very appropriate	Yes, very appropriate
Sample characteristics	Yes, there's a sufficient description	No	There's some description, but not enough	No	Yes, there's a sufficient description	Yes, there's a sufficient description	There's some description, but not enough	Yes, there's a sufficient description
Data collection	Yes, but more details are needed	No	Yes, but more details are needed	Yes, but more details are needed	Yes, data collection is clearly described	Yes, but more details are needed	Yes, but more details are needed	Yes, but more details are needed
Data recording methods	No	No	Yes, but only briefly mentioned	Yes, but only briefly mentioned	Yes, and reported well	No	No	No
Data collected addressing aims	Yes, completely	Yes, completely	Yes, completely	Yes, completely	Yes, completely	Yes, completely	Yes, completely	Yes, completely
Methods of analysis	No	Yes, but more detail is needed	No	No	Yes, and stated well	No	Yes, but more detail is needed	Yes, and stated well
Quality checks	No	No	No	No	No	No	No	No
Bias and positionality	No	No	No	No	Yes, but there's only a brief mention of this	Yes, but there's only a brief mention of this	No	No
Who conducted the research	Yes, a little	No	No	No	Yes, plenty	Yes, a little	No	Yes, a little
Data supporting the claims	Yes, there's plenty of clear, contextualised, relevant data	Yes, there's some data, but it's weak	Yes, there's some data, but it's weak	Yes, there's some data, but it's weak	Yes, there's plenty of clear, contextualised, relevant data	Yes, there's some data, but it's weak	Yes, there's plenty of clear, contextualised, relevant data	Yes, there's some data, but it's weak
Diverse viewpoints	Yes, only occasionally	No	No	Yes, only occasionally	Yes, only occasionally	Yes, thoroughly	Yes, thoroughly	No
Evidence addressing aims	Yes, every aim has some related evidence	Yes, every aim has some related evidence	Yes, every aim has some related evidence	Yes, every aim has some related evidence	Yes, every aim has some related evidence	Yes, every aim has some related evidence	Yes, every aim has some related evidence	Yes, every aim has some related evidence
Research questions answered	Yes, with a clear, logical, thorough answer	Yes, with a clear, logical, thorough answer	Yes, with a clear, logical, thorough answer	Yes, with a clear, logical, thorough answer	Yes, with a clear, logical, thorough answer	Yes, with a clear, logical, thorough answer	Yes, with a clear, logical, thorough answer	Yes, with a clear, logical, thorough answer
Discussion of literature, theory, or practice with results	Yes, but only briefly or generally discussed	No	No	No	Yes, and discussed well	No	Yes, but only briefly or generally discussed	Yes, but only briefly or generally discussed
Triangulation	Yes, strong triangulation	Yes, weak triangulation	Yes, weak triangulation	Yes, strong triangulation	Yes, strong triangulation	Yes, strong triangulation	Yes, strong triangulation	Yes, strong triangulation
Weaknesses	Yes, but weaknesses are mentioned without much discussion	No	No	Yes, but weaknesses are mentioned without much discussion	Yes, but weaknesses are mentioned without much discussion	No	Yes, but weaknesses are mentioned without much discussion	No
Ethical issues	No	No	No	No	Yes, beyond just the IRB and consent, if needed	No	No	No

## APPENDIX B FULL RISK OF BIAS ASSESSMENT

The full risk of bias assessment for each included study used in the review synthesis is presented in Table B1. A total of 22 assessments were conducted, as 16 papers reported the impact of one programme and three papers covered two different aquaculture programmes. These are clearly identified in the table. Moreover, 19 of these studies were quasi‐experimental designs, and three were randomised controlled trials, which included a few more bias domains.

**Table B1:** Risk of Bias assessment for each included study

**Study author (year)** **Programme**	Selection bias	Confounding	Performance bias	Spill‐overs, cross‐overs and contamination bias	Outcome measurement bias	Reporting bias	Assignment mechanism (RCT only)	Unit of analysis (RCT only)	Overall RoB
Akinlade (2012) (Fadama II)	Probably no	No	Yes	Probably no	Probably yes	Probably no	‐	‐	High risk of bias
Alawode and Oluwatayo (2019) (Fadama III)	Unclear	Probably no	Probably yes	Unclear	Probably yes	Probably no	‐	‐	High risk of bias
Amankwah et al. (2018) (ESP)	Unclear	Unclear	Probably yes	Probably no	Probably yes	Unclear	‐	‐	High risk of bias
Cahyadi and Bahramalian (2019) (SMART‐Fish)	Probably yes	Probably no	Probably yes	Unclear	Probably yes	Unclear	‐	‐	High risk of bias
DANIDA (2008) (GNAEP)	Unclear	Probably no	Unclear	Probably no	No	Unclear	‐	‐	High risk of bias
DANIDA (2008) (MAEP)	Unclear	Unclear	Unclear	Probably yes	Unclear	Unclear	‐	‐	Some concerns
Dey et al. (2010) (IAA)	Probably yes	Probably no	Probably yes	Unclear	Probably yes	Probably no	‐	‐	High risk of bias
Hallman et al. (2003) (MAEP)	Unclear	Probably no	Probably yes	Unclear	Probably yes	Unclear	‐	‐	High risk of bias
Hallman et al. (2003) (NGO BS)	Unclear	Probably no	Probably yes	Unclear	Probably yes	Unclear	‐	‐	High risk of bias
Haque and Dey (2017) (CBFC)	Unclear	Unclear	Unclear	Probably yes	Probably yes	Unclear	‐	‐	Some concerns
Haque and Dey (2016) (CBFC)	Unclear	Unclear	Probably yes	Unclear	Probably yes	Unclear	‐	‐	Some concerns
Khondker and Pemsl (2011) (DSAP)	Probably yes	Probably no	Unclear	Unclear	Probably yes	Unclear	‐	‐	High risk of bias
Kuijpers (2008, 2017, 2020) (SAFAL)	Probably yes	Probably yes	Probably yes	Unclear	Probably yes	Probably yes	‐	‐	Some concerns
Kuijpers (2019 (SAFAL)	Probably yes	Probably yes	Probably yes	Unclear	Probably yes	Probably yes	‐	‐	Some concerns
Michaux et al. (2019) (FoF)	Yes	Probably yes	Probably yes	Probably yes	Yes	Yes	Probably yes	Yes	Low risk of bias
Quisumbing and Kumar (2011 (MAEP)	Probably yes	Probably yes	Probably yes	Unclear	Unclear	No	‐	‐	High risk of bias
Quisumbing and Kumar (2011) (NGO BS)	Probably yes	Probably yes	Probably yes	Unclear	Unclear	No	‐	‐	High risk of bias
Rand and Tarp (2009) (MAEP)	Unclear	Unclear	Probably no	Probably no	Probably yes	Unclear	‐	‐	High risk of bias
Saiful Islam et al. (2015) (AFP)	Probably yes	Unclear	Probably yes	Unclear	Probably yes	Probably yes	‐	‐	Some concerns
Saiful Islam (2015) (AFP)	Probably yes	Probably yes	Probably yes	Unclear	Probably yes	Probably yes	‐	‐	Some concerns
Talukder and Green (2014) (FoF)	Unclear	Unclear	Probably yes	Unclear	Probably yes	Unclear	Unclear	Unclear	Some concerns
Verbowski et al. (2018) (FoF)	Probably yes	Yes	Probably yes	Probably yes	Yes	Probably yes	Probably yes	Yes	Low risk of bias

## APPENDIX C ADDITIONAL DATA FROM THE QUANTITATIVE SYNTHESIS

### C.1. Average effects analysis

Table C1 summarises the results of the quantitative synthesis for outcomes with at least two comparable effects, following the order in which these outcomes were discussed in the main results section. This includes full heterogeneity indicators and model results. All meta‐analysis models use independent effects with the exception of a model for income measures using RVE, which is clearly identified in the table below.

**Table C1:** Summary of main meta‐analysis results per outcome

Outcomes	Heterogeneity					Model results					
	*Q*	df	pval	T2	I2	Estimate	Std. Error	zval/tval	pval	CI lb	CI ub
Production Value	4.47	3	0.22	0.00	32.82	0.192	0.056	3.422	0.001	0.082	0.302
Production Volume	7.55	2	0.02	0.10	73.51	0.256	0.208	1.228	0.219	−0.152	0.664
Income (RVE)				0.10	86.68	0.251	0.057	4.450	0.001	0.127	0.376
Income	28.41	9	0.00	0.03	68.33	0.239	0.065	3.685	0.000	0.112	0.366
Total expenditures	9.83	4	0.06	0.02	56.66	0.159	0.078	2.042	0.041	0.006	0.311
Food expenditures	0.75	1	0.39	0.00	0.00	0.156	0.082	1.906	0.057	−0.004	0.317
Farm profit	3.55	2	0.17	0.01	43.71	0.150	0.091	1.649	0.099	−0.028	0.328
Household assets	5.13	1	0.02	0.07	80.50	0.039	0.211	0.185	0.854	−0.375	0.452
Poverty incidence	8.64	1	0.00	0.09	88.43	0.225	0.220	1.021	0.307	−0.207	0.656
Fish consumption	0.58	1	0.44	0.00	0.00	0.303	0.082	3.683	0.000	0.142	0.464
Women BMI	2.76	2	0.25	0.01	27.50	0.066	0.078	0.848	0.396	−0.086	0.218
Men BMI	0.00	1	1.00	0.00	0.00	0.067	0.095	0.707	0.480	−0.119	0.253
Child HAZ	0.04	2	0.98	0.00	0.00	0.052	0.062	0.840	0.401	−0.069	0.173

### C.2. Publication bias analysis

Egger's tests for publication bias were conducted for each outcome whenever possible. None of these were statistically significant, so we do not include funnel plots in this section.

### C.3. Sensitivity analysis

Figures C1, C2, C3, C4, C5, C6, C7 present the sensitivity analysis using the leave‐each‐out approach to assess if the average effect changes statistically or substantively when excluding each of the studies included in the overall effect analysis. We conducted this analysis for every outcome informed by at least three outcomes.

### C.4. Moderator analysis

Moderator analyses tested 18 potential variables to account for heterogeneity. These analyses were conducted using mixed‐effects models only for outcomes with at least four independent effects, and for categorical variables with “cell count" of at least two effects (i.e., the four numeric variables were always tested as moderators: programme size, exposure to intervention, evaluation period, and year of publication). Table C2 presents a summary of these analyses for the moderators and outcomes that met the above criteria.

**Table C2:** Summary of moderator analysis

Moderators	Outcome: production value	Outcome: income	Outcome: total expenditures
Continent		−0.31 (0.13)*	
Country		−0.26 (0.13)*	
Exposure to climate shocks		−0.31 (0.13)*	
World Bank country income group		−0.06 (0.18)	
Scale of intervention			
Programme size	0.00 (0.00)	0.00 (0.00)	−0.00 (0.00)*
Comparison group		−0.05 (0.17)	
Intervention components			
Productivity			
Income			
Nutrition		−0.04 (0.13)	0.16 (0.15)
Women's empowerment		−0.16 (0.12)	−0.22 (0.14)
Nutrition or Women's empowerment		−0.10 (0.15)	
Nutrition and Women's empowerment		−0.13 (0.13)	
All four components		−0.13 (0.13)	
Value chain			
Before production			
Production		−0.12 (0.17)	
Processing		−0.06 (0.18)	
Trading		−0.19 (0.13)	
Marketing		−0.06 (0.18)	
Any after production activity (processing, trading, or marketing)	0.07 (0.13)	−0.19 (0.13)	
Additional component(s) besides aquaculture activities			
Community‐focused intervention	−0.10 (0.13)	−0.15 (0.13)	
Exposure to intervention (in months)	−0.00 (0.00)	−0.00 (0.00)*	−0.01 (0.01)
Evaluation period (in months)			−0.00 (0.00)**
Peer‐reviewed and published paper	0.13 (0.11)	0.17 (0.12)	0.16 (0.15)
Year of publication	0.01 (0.01)	0.00 (0.01)	0.02 (0.01)
Evaluation design			
Assumptions made when extracting effect sizes due to missing data		0.11 (0.14)	−0.16 (0.15)
Risk of Bias	−0.10 (0.13)	−0.03 (0.14)	

*Note*: Moderator analysis estimates are presented with standard errors in parenthesis. Statistically significant estimates are denoted as: **p* < .05, ***p* < .01.

## APPENDIX D ADDITIONAL DATA FROM THE COST ANALYSIS

Table D1 presents a summary of the sources used, the relevant cost data and analysis that could be identified, and a brief assessment on the quality of these data per included programme.

**Table D1:** Summary of cost analysis for included programmes

Programme name and cost data sources	Year/country	Type of programme	Spent amount, budget, expenditure	Benefit cost ratio (CBR), costs and benefits	Comment
AFP Pant et al. (2014) WorldFish (2009)	2007–2009 Bangladesh	Diversifying livelihood options. Mostly aquaculture and value chain associated with fishery activities	A small project from WorldFish, expenditure broken down by type of purchases, not linked to output. Total Euro in 2008, €296,227 or $421,000	3600 households were reached. One benefit cost reported is the cost per household with respect to asset development. The costs amount to $50. Project manages to reach households at a budget of $138 per household	A small project such as this could have calculated cost of different components of the programme. It is not clear what counts as asset development
CBFC Haque and Dey (2017)	2007–2009 Bangladesh	Six flood plains of Bangladesh in three distinct sites. All fishery‐related with enclosures built and fingerlings provided	No budget is provided; but average costs are provided and broken into fixed and variable costs. The costs per household was $206, fixed cost was at $153 and $51 for variable costs	The household income reported was at $206 attributed to the project. There is some spill over savings for other agricultural activities. We report the benefit cost ratio to be 1.85. The project affected 778 households	More detailed costing could have been done. It is not clear the definitions used for fixed or variable costs. The CBR in the study may have been miscalculated
DSAP Mandal et al. (2004)	2000–2005 Bangladesh	Aquaculture project to promote fishery in ponds and rice fields covering 57 districts in Bangladesh, funded through USAID	Original funding from 2003 amounts to $7.66 million budget. No expenditure reported	Aimed to reach 210,000 households, and had 35,000 demonstration households. The cost per contacted household is about $200	This is a report of the processes, not of impacts
ESP Amankwah et al. (2018)	2009 Kenya	Overall fiscal stimulation project, reports from benefits to those who took up aquaculture	Difficult to know whether the allocation was for expenditure on aquaculture	Not sure what the total impact was. About $17.87 million was spent in 2009 as stimulation package. The evaluation occurs in 2013	People participated in a project that was part of a stimulation package. It is not possible to ascertain any unit costs
Fadama III World Bank (2016)	2009–2013 Nigeria	This is a large project with multiple components. Fishery is only a small part. There are a series of programmes whose impact is seen together. Budgetary allocations are periodic	There is no comprehensive evaluation of the project for a fixed budget. There is an impact evaluation for a 15‐year period, which covers multiple projects. An approximate budget is $319 million	The target number of households is about 965,000. Thus, one can claim so far for a project period of 11 years $350 per household. There are 17 income‐generating activities, for which internal rate of return and BCR is offered for earnings over 15 years. BCR for fishery activity is 1.51	The authors offer no account of how the BCR were calculated. Perhaps some account of that would have revealed what the costs for the fishery project would be
MAEP GNAEP DANIDA (2008), Annex 4	MAEP: 1989–2003 GNAEP: 1998–2006 Bangladesh	Two different sites to affect the income of people in tribal regions. These were large programmes	Budgets for the two different regions in chronological order: $30.2 million (MAEP) and $7.8 million (GNAEP)	It is possible to calculate how many households were affected and the unit costs. A total of 206,000 households were affected. Per household costs per annum were $19.5 $24.4 from 3 periods	There are no benefit measures to report. The unit costs may be lower as a budget is usually not completely spent on actual services
SAFAL Kessler et al. (2017); Kuijpers (2020)	2012–2016 Bangladesh	The project has other components than aquaculture development. The two papers see the project in different lights. Kessler sees this as an aquaculture project; Kuijpers sees the project as smoothing out of supply chains for farming activities	Both report a budget of €12 million, which is taken to be the cost. There is no disaggregation of costs	Kessler reports the cost of reaching a household to range from $93 to $145. The number reached range from 110 to 170,000. Kuijpers reports household affected as 59,000. The cost of reaching a household for the entire project per year is $268 to be compared to earnings of $436.00	The two papers report different unit costs as the effectiveness is measured very differently. Kuijpers talks of sustainability by assuming nearly all expenditure is fixed cost for future usage, which most likely not to be accurate. A previous version of this paper does not consider this measure
SMART‐Fish UNIDO (2019)	2015–2019 Indonesia	Fishery project to motivate the overall economics of fishery in the country, trade and production	Reports $3.787 million as the expenditure. Spending accounted equals $3.26 million, and that not accounted $0.47 million	Project is deemed successful, raising more money for the purpose at hand, more scientific process in fishery	It would be difficult to talk about unit cost. Expenditure could have been more detailed, but the project does not concern replication issues
BS No data available					
Fadama II No data available					
FoF No data available					
IAA No data available					
